# Intermittent fasting for weight loss in night shift workers: a three-arm, superiority randomised clinical trial

**DOI:** 10.1016/j.ebiom.2025.105803

**Published:** 2025-06-18

**Authors:** Maxine P. Bonham, Gloria K.W. Leung, Michelle Rogers, Rochelle Davis, Angela B. Clark, Corinne Davis, Yan Yin Phoi, Robert S. Ware, Asmaa El-Heneidy, Nicole J. Kellow, Jillian Dorrian, Siobhan Banks, Tracey L. Sletten, Catherine E. Huggins, Alison M. Coates

**Affiliations:** a Department of Nutrition, Dietetics and Food, Monash University, Notting Hill, Victoria, Australia; b Behaviour-Brain-Body Research Centre, University of South Australia, Adelaide, South Australia, Australia; c Alliance for Research in Exercise, Nutrition and Activity (ARENA), University of South Australia, Adelaide, South Australia, Australia; d Allied Health and Human Performance, University of South Australia, Adelaide, South Australia, Australia; e Griffith Biostatistics Unit, Griffith University, Gold Coast, Queensland, Australia; f School of Psychological Sciences, Turner Institute for Brain and Mental Health, Monash University, Melbourne, Victoria, Australia; g Global Centre for Preventative Health and Nutrition, Institute for Health Transformation, Deakin University, Geelong, Victoria, Australia

**Keywords:** Intermittent fasting, Shift work, Obesity, Weight loss, Continuous energy restriction, Diet intervention

## Abstract

**Background:**

Weight gain and an increased risk of developing type 2 diabetes are recognised consequences of night shift work. We tested the effectiveness of two modified intermittent fasting (IF) strategies compared with continuous energy restriction (CER) on weight loss and improvements in insulin resistance in night shift workers.

**Methods:**

A parallel group, three-arm randomised superiority clinical trial (Melbourne and Adelaide) recruited night shift workers (aged ≥ 25 and ≤ 65 years) with overweight/obesity. Participants were randomised by minimisation (site, age, gender) 1:1:1 to CER or one of two modified IF strategies. For 24 weeks participants followed a diet of 2100 kJ/d for two days of the week (modified IF) either on days off/day shift (IF:2D) or whilst on night shift (IF:2N) and followed their usual diet on the remaining five days. Co-primary outcomes were body weight and insulin resistance measured by the Homeostatic Model Assessment for Insulin Resistance (HOMA-IR) at 24-weeks. Participants and those assessing weight outcomes were not blinded to group assignment. Researchers assessing biochemical outcomes and study statistician were blinded to group assignment. Between-group differences were calculated using mixed-effects regression models.

**Findings:**

From October 2019 to February 2022, 250 participants (CER = 85; IF:2D = 83; IF:2N = 82) were randomised (mean (SD) age 46.8 (9.8) years; 133 women (53.2%)), with 170 (68%) completing. No significant difference between either modified IF strategy and CER for body weight; IF:2D vs CER (mean difference (MD) = −0.2 kg [95% CI −6.4 to 5.9]) and IF:2N vs CER (MD = −0.6 kg [95% CI −6.8 to 5.6]) and insulin resistance; IF:2D vs CER (MD = −0.1 [95% CI −1.0 to 0.8]) and IF:2N vs CER (MD = −0.3 [95% CI −0.5 to 1.2]) were observed. For secondary outcomes, those randomised to IF:2D had lower total and LDL cholesterol compared to CER ((MD = −23.2 mg/dL [95% CI −34.8 to −11.6]) and (MD = −19.3 mg/dL [95% CI −30.9 to −7.7]) respectively). In a completer's analysis, favourable changes in body weight, insulin resistance, body composition, blood pressure, and cardiometabolic markers were observed within all groups. No serious trial related adverse events were reported.

**Interpretation:**

At 24 weeks, weight and HOMA-IR were not different between the modified IF groups compared with CER. Clinically significant improvements in weight and metabolic health were achieved for the majority of night shift workers who remained in the intervention at 24 weeks.

**Funding:**

10.13039/501100000925National Health and Medical Research Council (APP1159762). ANZCTR registry, ACTRN-12619001035112.

**Research in context**

**Evidence before this study**

Prior to this study, we conducted a series of prospectively registered systematic reviews which found that night shift workers experience greater rates of weight gain compared to their day working counterparts and that the physiological mechanisms underlying this weight gain do not appear to be related solely to overall energy intake, but are rather a function of meal timing. Unusual eating times can disrupt the circadian system and in prior experimental studies we have shown that eating late into the night can generate abnormal metabolic responses to food intake such as hyperlipidaemia and hyperglycaemia, which are independent risk factors for CVD and type 2 diabetes (T2D). As such, weight loss strategies for night shift working populations may need to consider both meal timing and energy restriction. In a previous pilot study in a shift-working population we observed that minimising energy intake in the overnight period was feasible and resulted in small improvements in body weight; but long term interventions targeting metabolic health are limited. Twice per week fasting, a form of intermittent fasting commonly known as the 5:2 diet, whereby energy intake is greatly reduced on two days per week provides a novel and flexible strategy for night-shift workers to restrict energy at the times of the day associated with marked adverse metabolic outcomes. In 2015 our group conducted a systematic review examining the effectiveness of intermittent energy restriction compared with daily energy restriction. We searched CINAHL, Embase, Medline, PsycINFO, Cochrane, and Scopus for studies published up to January 2015, in English language. Eight studies were identified, with results finding comparable effectiveness of intermittent energy restriction compared with daily energy restriction for weight loss. In the past 7 years, after starting our trial, numerous systematic reviews have been published, confirming these early findings. Despite the success of twice per week fasting, its efficacy in populations that have a high incidence of obesity and unusual working schedules has not been tested, with the majority of RCTs completed to date of small to medium sample size.

**Added value of this study**

The current study is the longest weight loss intervention in night shift workers and provides evidence of the effectiveness of a common intermittent fasting intervention: twice-per week fasting (modified intermittent fasting) on weight, insulin resistance (HOMA-IR), and related outcomes in night shift workers with overweight and obesity. At 24 weeks after enrolment to a weight loss intervention, participants randomly assigned to consume a very low energy diet on two days per week (either on days off/day shift (IF:2D) or whilst on night shift (IF:2N)) and follow their usual diet on the remaining five days, weighed on average significantly less (8.5 and 5.5 kg respectively) than they did at the start of the intervention. Participants who moderately restricted their energy intake continuously throughout the week (CER) weighed 5.4 kg less on average than they did at the start of the intervention, comparable to weight loss reported in non-shift working populations. There were no statistically significant differences in weight or HOMA-IR between either modified intermittent fasting group and the CER group at 24 weeks, the IF:2D group experienced reductions in total and LDL cholesterol.

**Implications of all the available evidence**

The working conditions of a typical shift worker are not conducive to good health. Just by turning up to work each night, a shift worker increases their odds of developing obesity and T2D. These risks remain even after controlling for lifestyle and socioeconomic status. The within group improvements in key metabolic risk markers such as blood pressure, waist circumference and cholesterol, seen in all groups are promising in this population. Night shift workers with overweight or obesity could consider restricting energy intake either intermittently across 2 days per week or continuously through the week to achieve clinically significant weight loss over 6 months. These findings are important to develop evidence-based treatments in this population to help mitigate their higher risk of developing non-communicable diseases.

## Introduction

Night shift workers have little choice but to eat at times of the day typically associated with sleeping. Compared with non-night shift working populations, night shift workers have a propensity to gain weight and a disproportionately greater risk of developing cardiovascular disease, type 2 diabetes, and potentially some cancers,^1^, ^2^, ^3^, ^4^, ^5^ even in the absence of increased energy intake.^6^ The physiological mechanisms underlying the increase in disease risk linked to night-time eating include impaired glucose tolerance, insulin resistance and reduced postprandial energy expenditure and are supported by observations from highly controlled simulated shift-work studies.^7^, ^8^, ^9^ These consequences may be further impacted by the barriers to eating healthily during night shift that are extensively reported.^10^^,^^11^

Experimental studies have highlighted beneficial outcomes on body weight, body composition, and metabolic markers when timing of eating is prioritised to occur during daytime hours.^12^ Under controlled laboratory conditions benefits on glycaemic control are observed after consuming a meal in the morning compared with later during the day^13^ and benefits to glycaemic control occur even in the presence of mis-timed sleep.^14^ Yet, there is a paucity of weight-loss interventions designed specifically for night shift working populations that acknowledge the physiological and behavioural challenges of their work schedule.^15^^,^^16^ In a pilot intervention in night shift workers, avoiding eating between 1am and 6 am was associated with a small reduction in weight^17^ and provided feasibility of minimising night-time eating in night shift workers as well providing some understanding of the associated barriers.^18^ Recognition of the circadian disruption experienced by night shift workers should help in the design of effective weight-loss interventions, but also provide opportunities to target improvements in metabolic outcomes such as glycaemic control and insulin resistance. For interventions in shift working populations to be effective, consideration of meal timing, fasting duration, and minimising energy intake overnight is important, as is provision of strategies that are simple to implement and maintain long-term.

Intermittent fasting (IF) protocols refer to repetitive periods of alternating fasting and *ad libitum* eating periods and includes fasting on alternate days (ADF), fasting on one to two (consecutive or non-consecutive) days per week e.g., twice-per-week-fasting and time-restricted eating. Modified fasting prescribes consumption of 20–25% of energy requirements (approximately 2100 kJ/day or 500 kcal/day) on ‘fast’ days (with no timing prescribed) and dietary intake without energy restriction i.e., *ad libitum* on the other days.^19^ A commercialised example of modified (twice-per-week) fasting is the 5:2 diet. In non-shift working populations, weight reduction with modified fasting is comparable to that achieved by sustained periods of moderate (approximately 20%) continuous energy restriction (CER)^20^^,^^21^ but may promote greater improvements in insulin resistance,^22^ although findings are not consistent.^21^ Compliance to modified fasting protocols appears to be at least comparable to CER.^23^ The National Institute for Health and Care Excellence suggests tailoring components of weight management programmes to take into consideration a person's lifestyle alongside a flexible approach to energy reduction.^24^ As such, modified fasting may be an attractive proposition for shift workers, because ‘fast’ days can be tailored around shift scheduling. Furthermore, designating ‘fast’ days to align with night shift work, may result in additional improvements in metabolic health outcomes over more traditional weight loss strategies, due to reduced energy intake and postprandial metabolic excursions at night. Despite the emerging success of modified twice-per-week-fasting as a weight loss strategy, its efficacy in populations with i) non-traditional working schedules and ii) a higher risk of developing overweight and obesity than those in non-shift work roles remains untested. We compared the effectiveness of two modified IF strategies: IF:2D (two fast days per week on a day shift/day off) and IF:2N (two fast days per week each incorporating a night shift) with CER, on weight loss. CER was chosen as the active control as we wanted to achieve similar weight loss, whilst testing the effects of different approaches to IF intervention. We hypothesised *a priori* that all three diets would be effective for weight loss, and that both IF:2D and IF:2N would improve insulin resistance beyond that of CER.

## Methods

### Study design and participants

The Shifting Weight using Intermittent Fasting in Night Shift Workers (SWIFt) study is an investigator-initiated, multicentre, parallel group, three-arm randomised superiority clinical trial. The trial was approved by Monash Health (HREC, RES 19-0000-462A), and the University of South Australia (HREC ID: 202379) Human Research Ethics Committees, and Ambulance Victoria Research Committee (R19-037). Participants provided written informed consent. The trial protocol was written in accordance with SPIRIT guidelines (Standard Protocol Items: Recommendations for Interventional Trials) is registered at ANZCTR.org.au (ACTRN-12619001035112), and has been published previously.^25^ The study protocol and statistical analysis plan are available in the Supplementary Materials.

The trial was conducted in Melbourne and Adelaide, Australia, in the clinical trials facilities of Monash University and the University of South Australia. Participants were recruited through advertisements on social media, radio, and newspapers, as well as through newsletters, emails, and fliers distributed through workplaces. Eligible participants were those with overweight/obesity, aged ≥ 25 and ≤ 65 years, working a minimum of two nights per week, and had been weight stable in the 3 months prior with a weight change of no more than 5 kg. Participants were excluded from the study if any of the following criteria applied; BMI < 28 kg/m^2^ for non-Asian, or BMI < 26 kg/m^2^ for Asian participants; diagnosed with diabetes or cardiovascular disease, or on drug therapy for diabetes; current or planned pregnancy or breastfeeding; diagnosed with PCOS or gastrointestinal disease e.g., Crohn's Disease; obesity due to secondary causes i.e., genetic disorders (e.g., Down's Syndrome, Prader Willi) or endocrinology related disorders (e.g., hypothyroidism or growth hormone deficiency); commencement of a new medication or dosing regimen that alters body composition or metabolism (assessed on a case-by-case basis); previous weight loss surgery; consumption of 4 or more standard drinks on one occasion at a daily or almost daily occurrence; inability to complete the 6-month weight loss intervention i.e., due to severe dietary allergies/intolerances, or planned absences from night shift work.

### Randomisation and masking

After baseline testing, participants were randomly allocated to one of three interventions (1:1:1 ratio) using minimisation on study site, age (25–<39, 39–<53 or 53–≤65 years) and sex. Allocation sequence generation and implementation occurred independently by the National Health and Medical Research Council Clinical Trials Centre (University of Sydney, Australia) interactive voice response system. Study personnel enrolled participants and assigned them to interventions. Study personnel undertaking body weight assessments could not be masked to treatment assignment as they provided dietetic consults. Personnel running biochemical measures and study statisticians were masked.

### Procedures

The interventions were continuous energy restriction (CER) or one of two twice per week fasting IF strategies. Each intervention aimed to reduce energy intake by approximately 20% of the estimated energy requirements. Over the 24-week intervention period, participants met individually with a research dietitian with a clinical background in weight management on nine occasions. Participants allocated to IF were asked to restrict their energy intake to 2100–2500 kJ for two days per week in conjunction with five days of habitual dietary intake. For the IF:2D group, the two fasting occasions were allocated on days that included a day shift or a rostered day off. For the IF:2N group, the two fasting occasions were allocated on days that included a night shift. Participants allocated to CER received individualised dietetic support to reduce their daily energy intake by approximately 20% of energy requirements, through shifting their food consumption to that recommended by the Australian Guide to Healthy Eating.^26^ Study foods were provided to all groups for the 24-week period, and consumed on fasting days (IF:2D and IF:2N) or incorporated into CER diets. These were commercially available lower energy density packaged food items (frozen meals and shelf-stable snacks). Options were provided to tailor to individual preferences but typically consisted of one main meal and two snacks. No restriction was placed on energy distribution on the fast days. The emergence of the COVID-19 pandemic during the study resulted in dietetic consults moving to a tele-health model.^25^

### Outcomes

Pre-specified co-primary outcomes were body weight and the Homeostatic Model Assessment for Insulin Resistance (HOMA-IR). Primary outcome data were collected at baseline, 12, and 24-weeks with the participants having undertaken a minimum 10 h overnight fast. Body weight was measured twice per visit using medical grade scales (Melbourne; SECA 515, Ecomed, Adelaide; SECA 703). Additional weight measures (non-primary data) were undertaken at each dietetic consult either collected in the clinics using medical grade scales or at home using standardised Bluetooth weighing scales provided to all participants (Withings, Issy-les-Moulineaux, France). Once the COVID-19 pandemic began, most dietetic consult weights (excluding the primary outcome time-points of baseline, 12, and 24-weeks) were collected on the provided Bluetooth home-scales. HOMA-IR was calculated using the formula [fasting glucose (mmol/L) × fasting insulin (μmol/L)/22.5]^27^ from fasted blood samples run in duplicate.

Pre-specified secondary outcomes were also collected at baseline, 12, and 24-weeks in clinic. Secondary outcomes were body composition (Body Mass Index (BMI), waist circumference, fat, and fat free mass), cardiometabolic risk markers (glucose, insulin, lipids, HbA1c, and blood pressure), physical activity, sleep, and quality of life. Body composition was determined using dual-energy X-ray absorptiometry (Lunar, GE Healthcare, Madison, Wisconsin, USA), waist circumference was measured in duplicate using standardised procedures. Total fat mass (kg) and total fat free mass (kg) were analysed using enCORE software, version 18 (GE Healthcare, Madison, Wisconsin, USA). A fasting venous blood sample was collected, after a minimum 10 h fast, for measurement of glucose, insulin, plasma lipids, and HbA1c. Participants did not attend the study site directly after completing a night shift. Fasting blood pressure was taken in triplicate. Sleep episode duration during successive night shifts and days off were calculated as the time between self-reported usual sleep onset and wake time. Self-reported physical activity (total MET-minutes/week) was measured using the long-form version of the International Physical Activity Questionnaire (IPAQ) processing according to the 2005 guidelines. Gender, ethnicity, and occupation were self-reported. The Australian Bureau of Statistics (ABS) categorisations were used for occupation^28^ and ethnicity^29^ participant characteristic variables. Gender was self-reported based on the following options: 1 = Male, 2 = Female, 3 = Trans male/trans man, 4 = Trans female/trans woman, 5 = Non-binary/gender fluid, 6 = My gender is not listed. Quality of life was measured using the Assessment of Quality of Life-8D.^30^ Dietary intake was self-reported by each participant before the baseline and 24-week visits. Details on how to record dietary intake which included the provision of information relating to timings, brands, and cooking methods were provided by the research dietitians to all participants. Dietary intake was collected using 7-day food diaries either as a paper record or recorded electronically using the ‘Research Diary App’ (Xyris Software, Australia). Research dietitians worked through the completed 7-day food diaries with each participant at each time point to minimise reporting errors and increase accuracy of the data collected. Intervention adherence was measured by comparing daily energy intake from 7-day food diaries (kJ) at baseline with daily energy intake at 24-weeks (Xyris Software Pty Ltd, Australia).

### Biochemical analyses

On collection, blood samples were centrifuged, with aliquots stored at −80 °C until analysis. Serum lipid and plasma glucose concentrations were measured using the Indiko Clinical and Specialty Chemistry System (Thermo Fisher Scientific, Vantaa, Finland). Plasma insulin concentration was measured using the Human Insulin Specific RIA kit (HI-14K, Merck Millipore, Massachusetts, USA) according to manufacturer's instructions. All samples were run in duplicate, samples with a CV of >10% between duplicates were rerun. Haemoglobin A1c (HbA1c) was measured from whole blood using the capillary electrophoresis method (CAPILLARYS 3 OCTA, Sebia, Lisses, France).

### Adverse events

Adverse events (AE) and serious adverse events (SAE) were assessed at each dietetic visit. All adverse events were reported at the quarterly scientific advisory group meeting; events that were deemed possibly related to the interventions were reported to an independent medical expert.

### Statistical analysis

We aimed to enrol 423 participants, expecting to collect 24-week data on 351 (assuming 27% dropout). These recruitment numbers allow identification of differences between CER and each of the modified IF groups of 0.5SDs or greater with 80% power and alpha = 0.0125 (using Bonferroni correction, alpha = 0.05).

Each primary outcome (weight and HOMA-IR) was compared between groups using mixed-effects linear regression models with intervention and time-point included as main effects, with an intervention-by-time interaction term. Participant was included as a random intercept, and an autoregressive correlation structure with lag 1 was used. Effect estimates are presented as mean difference (MD) and 95% confidence interval (95% CI). For secondary outcomes, between- and within-group differences were analysed with similar mixed-effects linear regression models. The mixed-effects models use all available data, and provide correct inferences using the missing at random (MAR) assumption.^31^ The outcome physical activity, was analysed using median regression as data were skewed. The association between intervention and time-to-drop-out was analysed using the logrank test. Assumptions underlying all models, including residual diagnostics, were examined and confirmed to hold.

Statistical analyses were performed as prespecified in the Statistical Analysis Plan (available in the Supplementary Materials) which was finalised before unmasking, and the analysts were masked to intervention group until analyses were complete. Analyses were conducted according to the intention-to-treat principle. For primary outcomes, P < 0.0125 was considered statistically significant. Missing data were not imputed for the main analyses, however the sensitivity of outcomes to missingness was investigated using multiple imputation by chained equations. Additionally, a series of sensitivity analyses for the co-primary outcomes were conducted and are described below. For secondary outcomes and subgroup analyses, formal adjustment of confidence intervals for multiplicity was not performed, and findings should be interpreted as exploratory and hypothesis-generating. Analyses were performed with Stata statistical software, version 17.0 (StataCorp, College Station, TX, USA).

### Sensitivity analysis for primary outcomes

A series of sensitivity analyses were performed to investigate the robustness of the results for the co-primary outcomes. First, results from the mixed-effects linear regression with intervention group and time included as covariables, as well as a group-by-time interaction term, and participant included as a random effect are displayed. Second, results from first model with the addition of stratification factors as covariables are displayed. Third, results from a mixed-effects model with baseline values included as a separate covariable are displayed. Fourth, for body weight only, results from the same mixed-effects linear regression as the first model, but including all ‘home scales’ body weights are displayed. Fifth, results from the same mixed-effects linear regression as the first model, but only analysing data from individuals who provided complete data (i.e., each participant provides data at baseline, 12-weeks, and 24-weeks) are displayed.

The sensitivity of findings to missing data was investigated using multiple imputation with chained equations. Analyses were conducted using the ‘mi impute chained’ command in Stata v14.1. A linear regression imputation model was used. Covariables included in models were the baseline value for the outcome under investigation, age category, gender, site, and previous values of the outcome variable. The imputation process was iterated 100 times to capture the uncertainty associated with imputing missing values. The imputation was carried out through aggregating the data across groups rather than imputing data separately within each group, resulting in a conservative approach.

### Role of the funding source

The funders had no role in study design, data collection, analyses, interpretation of data, writing of the report, or decision to submit the article for publication.

## Results

**Fig. 1:**
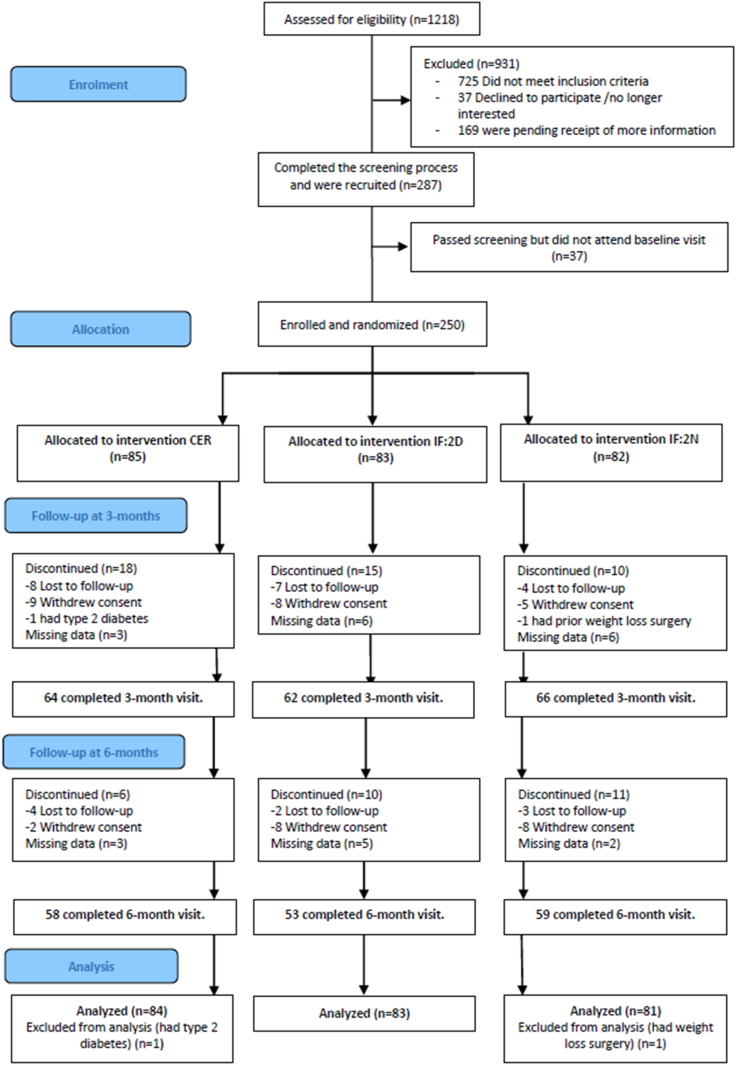
**Participant flow diagram**.

**Fig. 2:**
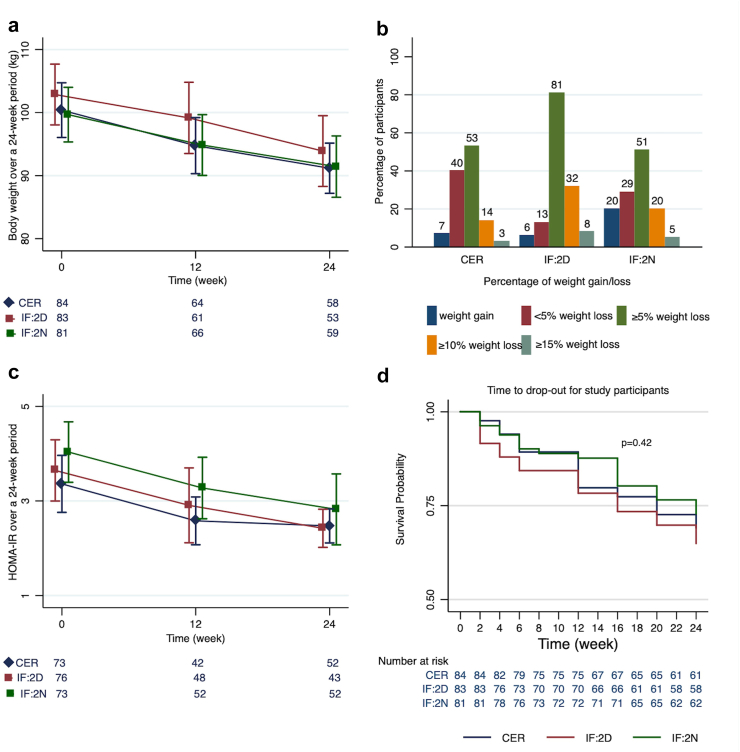
Panels a and c show the effect of dietary interventions on body weight and HOMA-IR at 12 and 24 weeks follow up, panel b shows the weight gain/loss at 24 weeks as a percentage of baseline body weight for completers. Panel d shows time to drop-out for participants who did not complete the study. Note the differing scales and minimum values on the Y-axes. Point estimates and error bars are offset for clarity. Error bars represent 2SE. P < 0.05 indicates a statistically significant result.

From September 2019 to February 2022, a total of 1218 participants were assessed for eligibility; 250 were enrolled, 85 to CER, 83 to IF:2D, and 82 to IF:2N (Fig. 1). Two participants were found to be ineligible after enrolment. A decision to cease recruitment prior to meeting our sample size was an outcome agreed with the scientific advisory group and reflected budgetary and timeline constraints due to the impact of COVID-19 on recruitment. A total of 170/248 (68%) and 149/248 (60%) participants provided primary outcome data for body weight and HOMA-IR at 24-weeks. Reasons for withdrawal from the SWIFt study were time constraints/scheduling (n = 10), personal reasons (n = 8), adverse events (unrelated to study) (n = 5), no reason provided (n = 4), difficulty meeting study protocol (n = 3), lack of improvement (n = 3), difficulty following diet (n = 2), adverse events (mood changes) (n = 1), commenced on a different (weight loss) treatment (n = 1), difficulty getting to clinic (n = 1), no longer interested (n = 1), and pregnancy (n = 1). For those lost to follow up (n = 28) no reason was provided. The lower participant numbers for HOMA-IR were due to the participants not providing a sample or being unable to withdraw blood. Our study found no significant association between study group and time to drop out (P = 0.42) with 24 (29%), 25 (33%), and 21 (27%) dropping out of the CER, IF:2D, and IF:2N groups respectively (Fig. 2). Demographic and clinical characteristics of participants at baseline are presented in Table 1. Participants (53% female, 47% male) had a mean (standard deviation; SD) age of 46.8 (9.8) years and BMI of 34.8 (5.9) kg/m^2^. Participants who did, and did not, provide 24-week data are compared in Supplementary Table S1. Participants with higher weight at baseline were less likely to complete the study, as were those with lower quality of life.

**Table 1:** Baseline characteristics by intervention group for the SWIFt study (n = 250).

Variable	CER (n = 85)	IF:2D (n = 83)	IF:2N (n = 82)
**Demographic characteristics**			
Age, mean (SD), y	46.4 (8.9)	46.9 (10.4)	47.1 (10.2)
Female, No. (%)	45 (52.9)	43 (51.8)	45 (54.9)
Shift work, mean (SD), y	15.5 (10.1)	15.7 (11.3)	16.3 (10.6)
Smoking, n (%)	10 (11.8)	10 (12.1)	4 (4.9)
Diabetes^a^, n (%)	1 (1.2)	0 (0.0)	0 (0.0)
Wight-loss surgery^a^, No. (%)	0 (0.0)	0 (0.0)	1 (1.2)
Self-reported ethnicity, No. (%)			
European	60 (70.6)	55 (67.1)	53 (64.3)
Asian/Oceania	10 (11.8)	9 (8.7)	6 (11.0)
Other	15 (17.6)	19 (23.2)	20 (24.7)
Self-reported shift schedule, No. (%)			
Permanent/fixed night shifts	45 (52.9)	47 (56.6)	41 (50.0)
Rotating night shift	30 (35.3)	22 (26.5)	24 (29.3)
Other	10 (11.8)	14 (16.9)	17 (20.7)
**Body composition**			
Body weight, mean (SD), kg	100.5 (19.8)	102.8 (21.9)	99.7 (19.3)
BMI, mean (SD), kg/m^2^	34.8 (5.7)	35.0 (6.8)	34.7 (5.2)
Fat mass, mean (SD), kg	41.3 (12.8)	39.9 (10.8)	39.8 (8.9)
Fat-free mass, mean (SD), kg	56.5 (11.8)	58.1 (12.6)	55.4 (12.6)
Waist circumference, mean (SD), cm	105.0 (14.9)	105.1 (16.8)	104.5 (15.7)
Energy intake, mean (SD), kJ	8732.6 (2912.1)	8866.4 (2891.8)	8384.1 (2560.1)
**Cardiometabolic risk factors**			
Systolic blood pressure, mean (SD), mmHg	132.5 (16.8)	131.5 (17.7)	127.8 (14.7)
Diastolic blood pressure, mean (SD), mmHg	85.1 (8.5)	83.0 (10.1)	82.7 (8.7)
Fasting glucose, mean (SD), mg/dL	97.3 (14.4)	99.1 (28.8)	99.1 (19.8)
Fasting insulin, mean (SD), mIU/L	13.4 (8.3)	14.2 (8.2)	15.9 (8.5)
HOMA-IR^b^, median (IQR)	2.8 (1.8–4.0)	2.6 (1.9–4.9)	3.2 (2.2–4.9)
HbA1c (%)	5.4 (0.5)	5.5 (0.9)	5.5 (0.7)
Triglycerides, mean (SD), mg/dL	132.7 (79.6)	130.2 (81.6)	153.4 (123.9)
Total cholesterol, mean (SD), mg/dL	225.1 (42.5)	219.0 (41.5)	220.1 (42.5)
LDL-cholesterol, mean (SD), mg/dL	143.2 (33.1)	137.3 (34.8)	137.7 (34.8)
HDL-cholesterol, mean (SD), mg/dL	53.5 (14.2)	54.5 (15.4)	54.5 (15.4)
Quality of life (AQOL-8D)^c^, mean (SD)	0.68 (0.16)	0.70 (0.16)	0.72 (0.16)
Sleep episode duration on night shifts, mean (SD), hrs	6.2 (1.9)	5.9 (1.8)	6.2 (1.6)
Sleep episode duration on days off, mean (SD), hrs	8.8 (1.3)	8.9 (1.5)	8.9 (1.3)
Physical activity^,^ total MET^d^, median (IQR), min/w	6413 (2919–11,346)	5052 (2228–9443)	7540 (3108–12,207)

Abbreviations: AQOL-8D, Assessment of Quality of Life-8 Dimension; BMI, body mass index; CER, continuous energy restriction; HbA1c, haemoglobin A1C; HDL, high-density lipoprotein; HOMA-IR, homeostatic model assessment for insulin resistance; IF:2D, modified fasting day shift/days off; IF:2N, modified fasting night shift; IQR, interquartile range; LDL, low-density lipoprotein; MET, metabolic equivalent of task; n (%), number (percentage); SD, standard deviation.

To convert values for glucose to mmol/L, multiply by 0.0555, cholesterol to mmol/L multiply by 0.0259, insulin to pmol/L, multiply by 6.945, triglycerides to mmol/L, multiply by 0.0113.

Missing items: CER, fat mass and fat-free mass n = 3, waist circumference n = 2, energy intake n = 3, blood pressure n = 1, glucose/insulin/HOMA/HbA1c n = 12, triglycerides and cholesterol n = 8, sleep on night shift n = 13, sleep on days off n = 18, physical activity n = 3; IF:2D, fat mass and fat-free mass n = 2, waist circumference n = 1, energy intake n = 2, blood pressure n = 3, glucose/HOMA/HbA1c n = 7, insulin n = 6; triglycerides and cholesterol n = 7, sleep on night shift n = 8, sleep on days off n = 12, physical activity n = 3; IF:2N, fat mass and fat-free mass n = 3, waist circumference n = 3, energy intake n = 2, blood pressure n = 1, glucose/HOMA n = 9, insulin/HbA1c n = 8, triglycerides and cholesterol n = 7, sleep on night shift n = 12, sleep on days off n = 16.

a Two participants who had diabetes and weight loss surgery respectively were excluded from data analysis.

b HOMA-IR = fasting insulin (mIU/L) × fasting glucose (mmol/L)/22.5.

c AQOL-8D; Weighted utility score (Dimension Worst Health State = 0.00–Dimension Best Health State = 1.00).

d Data from International Physical Activity Questionnaire (IPAQ)-Long Form which measures self-reported time spent being physically active across multiple domains: transportation, recreation (including sport and leisure time), housework, job-related and time spent sitting.

### Primary outcomes

In an intention to treat analysis there were no significant between-group differences in body weight at 24-weeks (Table 2); IF:2D vs CER (MD = −0.2 kg [95% CI −6.4 to 5.9]; P = 0.94) and IF:2N vs CER (−0.6 kg [95% CI −6.8 to 5.6]; P = 0.85), however each of the three interventions were effective for weight loss (Supplementary Table S2). In those who provided outcome data at 24-weeks, clinically relevant weight loss (≥5%) was observed in 61% of participants (40% of total randomised). The mean (95% CI) weight loss was 5.3 kg (95% CI 3.2–6.3), 7.9 kg (95% CI 6.8–9.2), and 5.2 kg (95% CI 3.9–6.4) for the CER, IF:2D, and IF:2N groups respectively (n = 170, Table 3, Fig. 2a). HOMA-IR significantly decreased in those who provided outcome data at 24-weeks (n = 149), with mean (95% CI) decrease of 0.4 (95% CI 0.1–0.7), 0.9 (95% CI 0.5–1.2), and 0.9 (95% CI 0.4–1.4) units in the CER, IF:2D, and IF:2N groups respectively (Table 3, Fig. 2c), with no significant difference between interventions (Table 2); (IF:2D vs CER (−0.1 [95% CI −1.0 to 0.8]; P = 0.82) and IF:2N vs CER (0.3 [95% CI −0.5 to 1.2]; P = 0.59)). When sensitivity analyses were conducted, findings for both primary outcomes were similar after adjusting for stratification factors, (study site and age of participants, Supplementary Table S3), however, when body weight at 24-weeks was adjusted for baseline body weight, the IF:2D group had significantly lower body weight at 24 weeks than the CER group (MD = −2.7 kg; 95% CI −4.2 to −1.2). This difference was driven by participants providing outcome data from the IF:2D group being heavier at baseline compared with the CER group (mean (SD) = 102.4 kg (21.1) vs 96.6 kg (19.1)), as groups were relatively similar at 24-weeks (93.9 kg (20.4) vs 91.2 kg (15.2)) (Supplementary Table S3).

**Table 2:** Effect of group allocation on body composition and cardiometabolic risk factors at 24 weeks.^a^

At baseline	CER (n = 84)	IF:2D (n = 83)	IF:2N (n = 81)	IF:2D vs CER	IF:2N vs CER
Variable	Mean (SD)	Mean (SD)	Mean (SD)	Mean difference (95% CI)	Mean difference (95% CI)
**Primary outcomes**					
Body weight, kg	91.2 (15.2)	93.9 (20.4)	91.4 (18.7)	−0.2 (−6.4 to 5.9)	−0.6 (−6.8 to 5.6)
HOMA-IR^b^	2.5 (1.3)	2.4 (1.3)	2.8 (2.7)	−0.1 (−1.0 to 0.8)	0.3 (−0.5 to 1.2)
**Secondary outcomes**					
BMI, kg/m^2^	31.4 (4.5)	31.0 (5.2)	31.9 (4.6)	−0.6 (−1.5 to 2.1)	−0.2 (−2.0 to 1.6)
Fat mass, kg	34.2 (10.1)	31.6 (9.1)	36.0 (9.5)	−3.0 (−4.2 to 2.9)	−0.6 (−4.2 to 2.9)
Fat-free mass, kg	55.1 (11.2)	58.3 (12.9)	54.2 (13.1)	1.5 (−2.4 to 5.4)	−0.5 (−4.4 to 3.4)
Waist circumference, cm	97.8 (12.8)	95.8 (14.9)	95.2 (15.3)	−2.9 (−7.9 to 2.1)	−2.5 (−7.4 to 2.5)
Energy intake, kJ	6794.5 (1946.0)	6773.8 (1667.5)	6260.6 (1780.6)	−221.1 (−1151.5 to 709.5)	−532.7 (−1431.5 to 366.0)
Systolic blood pressure, mmHg	125.8 (13.3)	123.9 (15.1)	123.2 (15.1)	−1.6 (−7.5 to 4.6)	−2.1 (−7.4 to 3.2)
Diastolic blood pressure, mmHg	81.1 (6.8)	80.4 (7.9)	80.2 (8.0)	−0.4 (−3.9 to 2.7)	−0.7 (−3.6 to 2.2)
Fasting glucose, mg/dL	93.7 (7.2)	90.1 (7.2)	95.5 (12.6)	0.0 (−7.4 to 5.4)	1.8 (−5.4 to 7.4)
Fasting insulin, mIU/L	10.5 (5.2)	10.5 (5.7)	11.4 (7.8)	−0.1 (−0.4 to 0.3)	0.1 (−0.2 to 0.5)
HbA1c (%)	5.4 (0.3)	5.2 (0.4)	5.4 (0.5)	−0.02 (−0.25 to 0.21)	0.04 (−0.18 to 0.27)
Triglycerides, mg/dL	150.4 (88.5)	130.7 (79.7)	135.4 (106.2)	−17.7 (−53.1 to 17.7)	−26.6 (−61.9 to 17.7)
Total cholesterol, mg/dL	223.0 (46.3)	204.6 (34.8)	218.0 (42.5)	**−23.9 (−38.8 to −10.6)**	−8.1 (−12.3 to 7.9)
LDL-cholesterol, mg/dL	142.7 (34.8)	126.1 (34.8)	136.6 (34.8)	**−20.2 (−31.9 to −8.1)**	−6.5 (−18.3 to 5.1)
HDL-cholesterol, mg/dL	53.6 (14.1)	55.6 (12.1)	58.8 (17.4)	3.5 (−3.2 to 7.2)	3.9 (−0.9 to 11.6)
Quality of life (AQOL-8D)^c^	0.73 (0.14)	0.74 (0.17)	0.76 (0.16)	0.04 (−0.01 to 0.09)	0.04 (−0.01 to 0.09)
Sleep episode duration on night shifts, hrs	6.6 (1.6)	6.0 (1.6)	6.5 (1.9)	−0.4 (−0.9 to 0.3)	−0.1 (−0.7 to 0.5)
Sleep episode duration on days off, hrs	8.9 (0.9)	9.1 (1.3)	8.8 (1.3)	0.2 (−0.3 to 0.7)	−0.1 (−0.6 to 0.4)
Physical activity^,^ total MET^d^, ^e^, median (IQR), min/w	6798 (3060–11,862)	5016 (2484–8088)	7204 (3252–12,207)	−1782 (−3521 to 1317)	441 (−1272 to 2484)

Abbreviations: AQOL-8D, Assessment of Quality of Life-8 Dimension; BMI, body mass index; CER, continuous energy restriction; CI, confidence interval; HbA1c, haemoglobin A1C; HDL, high-density lipoprotein; HOMA-IR, homeostatic model assessment for insulin resistance; IF:2D, modified fasting day shift/days off; IF:2N, modified fasting night shift; IQR, interquartile range, LDL, low-density lipoprotein; MET, metabolic equivalent of task; SD, standard deviation.

The value in **bold** indicates a statistically significant result at a threshold of P < 0.05.

At 24 weeks 58, 53, and 59 people provided data for weight from the CER, IF:2D, and IF:2N groups respectively. Missing items for data collected at baseline, 12- and 24-weeks: CER: weight and BMI, 0, 19, 26, waist circumference n = 2, 40, 27, blood pressure n = 1, 13, 27, glucose/insulin/HOMA/HbA1c n = 11, 22, 32, triglycerides and cholesterol n = 8, 20, 30; IF:2D: weight and BMI, 0, 22, 30, waist circumference n = 1, 38, 35, blood pressure n = 3, 15, 35, glucose/HOMA n = 7, 15, 39, insulin/HbA1c n = 6, 14, 38, triglycerides and cholesterol n = 7, 14, 39; IF:2N: weight and BMI, 0, 16, 22, waist circumference n = 3, 41, 25, blood pressure n = 1, 17, 25, glucose/HOMA/HbA1c n = 9, 13, 28, insulin n = 8, 14, 29, triglycerides and cholesterol n = 6, 14, 28.

Missing items for data collected at baseline and 24-weeks: CER: fat mass and fat-free mass n = 3, 29, energy intake n = 3, 29, Quality of life, 0, 26, sleep on night shift n = 12, 33, sleep on days off n = 17, 35, physical activity n = 3, 26; IF:2D: fat mass and fat-free mass n = 2, 35, energy intake n = 2, 40, Quality of life, 0, 33, sleep on night shift n = 8, 38, sleep on days off n = 12, 44, physical activity n = 3, 33; IF:2N: fat mass and fat-free mass n = 3, 30, energy intake n = 1, 30, Quality of life, 0, 23, sleep on night shift n = 11, 33, sleep on days off n = 15, 31, physical activity n = 0, 23.

a Analyses were conducted with the use of a linear mixed-effects model, according to the intention-to-treat principal by original assigned groups.

b HOMA-IR = fasting insulin (mIU/L) × fasting glucose (mmol/L)/22.5.

c AQOL-8D; Weighted utility score (Dimension Worst Health State = 0.00–Dimension Best Health State = 1.00).

d Data from International Physical Activity Questionnaire (IPAQ)-Long Form which measures self-reported time spent being physically active across multiple domains: transportation, recreation (including sport and leisure time), housework, job-related and time spent sitting.

e Median regression analysis was used.

**Table 3:** Within-group changes in body composition and cardiometabolic risk factors over 24-week study period for completers.^a^

Number at baseline	CER (n = 58)		IF:2D (n = 53)		IF:2N (n = 59)	
Variable	Number at 24-weeks	Mean difference (95% CI)	Number at 24-weeks	Mean difference (95% CI)	Number at 24-weeks	Mean difference (95% CI)
**Primary outcomes**						
Weight, kg	58	**−5.3 (−6.3 to −3.2)**	53	**−7.9 (−9.2 to −6.8)**	59	**−5.2 (−6.4 to −3.9)**
HOMA-IR^b^	53	**−0.4 (−0.7 to −0.1)**	44	**−0.9 (−1.2 to −0.5)**	52	**−0.9 (−1.4 to −0.4)**
**Secondary outcomes**						
BMI, kg/m^2^	58	**−1.8 (−3.2 to −0.2)**	53	**−2.6 (−4.2 to −0.9)**	59	**−1.9 (−2.2 to −1.5)**
Fat mass, kg	55	**−4.3 (−5.7 to −3.1)**	48	**−5.8 (−5.7 to −2.5)**	51	**−4.0 (−5.4 to −2.5)**
Fat-free mass, kg	55	**−1.1 (−1.7 to −0.7)**	48	**−1.6 (−1.8 to −0.6)**	51	**−1.2 (−1.2 to −0.1)**
Waist circumference, cm	57	**−4.3 (−5.8 to −3.3)**	48	**−7.4 (−9.1 to −5.6)**	56	**−6.4 (−8.2 to −4.7)**
Energy intake, kJ	54	**−1934.5 (−2517.3 to −1351.8)**	42	**−2181.6 (−2718.3 to −1385.5)**	51	**−2120.4 (−2597.6 to −1643.9)**
Fasting glucose, mg/dL	53	−1.8 (−3.6 to 0.0)	44	**−3.6 (−5.4 to −1.8)**	53	−1.8 (−3.6 to 1.8)
Fasting insulin, mIU/L	54	**−1.8 (−2.9 to −0.7)**	45	**−3.2 (−4.5 to −1.9)**	52	**−3.8 (−5.2 to −2.4)**
HbA1c (%)	54	0.01 (−0.05 to 0.07)	45	−0.01 (−0.07 to 0.05)	53	0.0 (−0.07 to 0.07)
Systolic blood pressure, mmHg	57	**−6.7 (−9.1 to −4.2)**	48	**−7.4 (−11.8 to −2.9)**	56	**−4.0 (-6.7 to −1.3)**
Diastolic blood pressure, mmHg	57	**−4.1 (−5.6 to −2.6)**	48	**−2.3 (−4.5 to −0.1)**	56	**−2.3 (−4.0 to −0.7)**
Triglycerides, mg/dL	54	18.7 (−8.7 to 44.3)	44	−15.7 (−52.1 to 24.6)	53	−18.4 (−53.1 to 17.3)
Total cholesterol, mg/dL	54	−0.3 (−7.6 to 7.9)	44	**−15.4 (−23.1 to −7.7)**	53	−3.9 (−11.3 to 3.9)
LDL-cholesterol, mg/dL	54	0.2 (−7.6 to 7.8)	44	−**12.3 (−19.3 to −5.8)**	53	1.1 (−5.8 to 7.7)
HDL-cholesterol, mg/dL	54	0.8 (−0.9 to 2.6)	44	1.0 (−1.1 to 3.7)	53	3.9 (0.0–3.9)
Quality of life (AQOL-8D)^c^	58	0.03 (0.00–0.05)	50	**0.05 (0.01–0.08)**	59	0.03 (0.00–0.07)
Sleep on night shifts, mean (SD), hr/d	51	0.3 (−0.1 to 0.8)	45	**0.3 (0.0–0.7)**	48	0.3 (−0.2 to 0.7)
Sleep on days off, mean (SD), hr/d	49	0.1 (−0.2 to 0.5)	39	0.0 (−0.4 to 0.4)	50	−0.2 (−0.4 to 0.1)
Physical activity^,^ total MET^d^, ^e^, median (IQR), min/w	58	271.5 (−1151.2 to 1694.2)	45	−42.0 (−1288.1 to 1204.4)	54	−122.5 (−1896.2 to 1651.7)

Abbreviations: BMI, body mass index; CER, continuous energy restriction; CI, confidence interval; HbA1c, haemoglobin A1C; HDL, high-density lipoprotein; HOMA-IR, homeostatic model assessment for insulin resistance; IF:2D, modified fasting day shift/days off; IF:2N, modified fasting night shift; IQR, interquartile range; LDL, low-density lipoprotein; SD, standard deviation.

The value in **bold** indicates a statistically significant result at a threshold of P < 0.05.

a Analyses were conducted with the use of a linear mixed-effects model.

b HOMA-IR = fasting insulin (mIU/L) × fasting glucose (mmol/L)/22.5.

c AQOL-8D; Weighted utility score (Dimension Worst Health State = 0.00–Dimension Best Health State = 1.00).

d Data from International Physical Activity Questionnaire (IPAQ)-Long Form which measures self-reported time spent being physically active across multiple domains: transportation, recreation (including sport and leisure time), housework, job-related and time spent sitting.

e Median regression analysis was used.

### Secondary outcomes

IF:2D, compared to CER, was associated with significantly lower total cholesterol (MD = −23.9 mg/dL [95% CI −38.8 to −10.6]) and LDL cholesterol (MD = −20.2 mg/dL [95% CI −31.9 to −8.1]), but not other cardiometabolic outcomes (Table 2). Body composition, energy intake, physical activity, sleep outcomes, and quality of life were not significantly different between groups at 24-weeks (Table 2). Secondary outcomes were similar after multiple imputation (Supplementary Table S4). Significant within group changes were observed for all groups for fasting insulin, blood pressure, waist circumference, BMI, fat mass, and fat free mass. Fasting glucose and quality of life improved within the IF:2D group only. There were no changes in HbA1c, triglycerides or HDL cholesterol within any groups (Table 3, Supplementary Figures S1 and S2). Findings of sensitivity analysis using multiple imputation are displayed in Supplementary Table S4, and are consistent with the main analyses.

### Adherence

All groups reported a significant reduction in daily energy intake at 24-weeks compared with baseline (Table 3), we observed no significant difference between groups (Table 2).

### Adverse events

Two SAEs were reported during the 24 weeks of intervention and deemed unrelated to the study (one knee and hip replacement, and one appendectomy). During the weight loss phase, participants in the IF groups reported the common physical symptoms of headache (IF:2D 19%, IF:2N 22%, CER 0%, P < 0.001 for both IF vs CER comparisons) and gastrointestinal symptoms (IF:2D 7%, IF:2N 9%, CER 0%, IF:2D vs CER, P = 0.03 and IF:2N vs CER, P = 0.01) significantly more frequently than the CER group. A full list is provided in Supplementary Table S5.

## Discussion

Night shift workers are at a greater risk of weight gain than the general day-working population.^2^ Strategies to support weight loss are needed. This study did not find statistically significant or clinically important differences in weight and HOMA between the modified IF groups and the CER group. Although the pre-specified sample size targets were not met due to the COVID-19 pandemic, the magnitude of the observed between-group differences (maximum between-group MD for weight = 0.6 kg and for HOMA-IR 0.3 units) suggests the dietary strategies led to similar weight loss and insulin resistance outcomes. The IF:2D group experienced a greater decrease in total and LDL cholesterol over the 24-weeks compared to the CER group. Our study found an overall reduction in energy intake at 24-weeks compared to baseline, and there were no significant differences in energy intake or physical activity between groups. Improvements in cardiometabolic risk factors including blood pressure, waist circumference, BMI, fat mass, and fasting insulin occurred in all intervention groups. Retention rates were similar across all three interventions and only mild adverse effects, previously associated with modified fasting regimens, were reported.

Even with the physiological challenges associated with circadian disruption, the majority of night shift workers who completed the 24-week intervention achieved significant weight loss following CER or either of the two modified fasting strategies (IF:2D and IF:2N) where energy intake was restricted for two days a week. This finding is similar to published meta-analyses in non-night shift workers, whereby twice-per-week-modified fasting is similarly effective as CER in eliciting weight loss.^20^^,^^32^ We observed clinically relevant weight loss (≥5%) in 61% of night shift workers who completed the 24-week interventions (40% of those who commenced the study). This is an important finding as there is a dearth of studies exploring the feasibility of energy restriction in night shift workers. Several reviews have reported on workplace interventions in shift workers aimed at improving health outcomes,^33^ promoting healthier food intake^34^ and group-based interventions targeting a healthy lifestyle^35^ with one review focussing explicitly on dietary interventions for night shift workers.^36^ These reviews overall found few RCTs examining the effect of dietary interventions targeted night shift workers specifically, and these studies were mostly not effective for weight loss.

For those who completed the intervention to the 24-week time point (170/250 randomised, 68%), subtle but important differences in weight loss were observed between the groups, with participants allocated to IF:2D showing greater weight loss, compared with CER and IF:2N, after adjusting for baseline weight. A comparison of completers vs non-completers indicated that participants who stayed in the IF:2D group had a greater body weight at baseline. This may explain the difference observed in the sensitivity analysis; however, this will be more robustly interrogated in a future exploratory analysis of the relationship between interindividual differences and engagement with the study.

HOMA-IR was chosen as a co-primary outcome due to published data indicating the potential for modified IF strategies to improve HOMA-IR compared with CER.^19^^,^^20^ We hypothesised that an approach to modified fasting that minimised energy intake whilst on night shift (IF:2N) may provide additional benefit. In our study we observed that reductions in HOMA-IR were not significantly different between the two modified fasting groups and CER. A possible explanation could have been the differences in adherence to the three interventions, which in turn would have impacted caloric intake and/or timing of food consumption in the IF groups. However, a similar calorie deficit was observed across all groups (our nominated measure of adherence), drop out across groups was consistent (our measure of compliance) and checklists of consumed food collected at each dietetic consult indicated that participants consumed the food provided. Our data agrees with published data that typically report good adherence to modified IF protocols^23^ and also data that indicate that weight change is comparable when comparing CER with modified IF interventions.^20^^,^^32^ As we observed no difference in weight change nor changes in fat and fat free mass, we do not think differential metabolic adaptation occurred as a result of energy restriction between diet groups. Whilst our findings contrast with earlier studies that suggested greater improvements in HOMA-IR after a modified IF regimen compared with CER,^37^ they align with more recent data from larger, and longer trials and a meta-analysis, which found no difference in HOMA-IR between 5:2 and CER.^38^ Regardless, the mean change in HOMA-IR at the end of the 24-week intervention was favourable in all groups. Any improvement in markers of glycaemic control is of relevance for night shift working populations, due to their increased risk of developing type 2 diabetes.^39^ Beneficial changes to glycaemic control have been observed in a number of well-controlled studies utilising simulated shift-work protocols. In a small, controlled study, not eating at night prevented glucose intolerance following four nights of simulated night-shift work.^40^ In a second study by the same research group, the authors showed that simulated night-shift work impaired insulin sensitivity.^41^ Addition of a meal or snack to the simulated night shift impaired glucose tolerance compared with not eating at night. Avoidance of night time eating was associated with increased insulin secretion but glucose tolerance was not impaired by circadian misalignment.^41^ In one other study, using a highly controlled forced desynchrony design, Chellappa et al. were able to show that night-time eating led to misalignment between central and peripheral (glucose) endogenous circadian rhythms and impaired glucose tolerance.^14^ Consuming meals only during the day time was preventative. In rotating night shift workers completing 12 weeks of time-restricted eating (9am–7pm) in addition to standard of care (nutritional counselling and advice to follow a Mediterranean diet), those at elevated cardiometabolic risk reported significant reduction in diastolic blood pressure and HbA1c compared with standard of care alone.^15^ These data collectively show an influence of timing of food intake on glucose metabolism during simulated night-shift and a metabolic benefit to prioritising meal intake to the biological day.

Total cholesterol and LDL cholesterol were significantly lower in the IF:2D group compared to CER at 24-weeks and the decrease in total and LDL cholesterol of 23.2 mg/dL and 19.3 mg/dL respectively reflect clinically relevant reductions.^42^ Modified fasting interventions have shown superiority over CER in relation to cholesterol reduction in some trials, but not others,^43^ however these studies have involved alternate fast days (AFD) rather than two fast days a week. The metabolic switch from using glucose to ketones as the primary energy source, known as metabolic switching, has been purported as one mechanism for improvement in cholesterol status after IF interventions,^44^ however improvements in cholesterol (total and LDL) were observed in only one of the two IF groups. In a meta-analysis of lifestyle interventions targeting weight-loss (n = 73 RCTs) for every additional one kg of weight loss, LDL-C was reduced by −1.28 mg/dL (95% CI −2.19 to −0.37 mg/dL).^45^ We observed a larger change in weight loss within the IF:2D group which could have contributed to the reduction in total- and LDL-cholesterol observed in the IF:2D group compared to CER. However, it would be important to validate this finding in further studies. The SWIFt study found no other significant differences between groups for other secondary outcomes including measure of body composition and cardiometabolic health.

All strategies were associated with within-group improvements in fasting insulin, BMI, fat mass, and waist circumference on average, reflecting expected improvements in metabolic health after weight loss.^46^ These results should be interpreted in the context of accelerated worsening of health markers over time compared to those who do not work night shifts, which has been observed in as little as 12 months after night shift commences.^47^ Among 359,387 participants in the European Prospective Investigation into Cancer and Nutrition (EPIC) study, for every 5 cm decrease in waist circumference, the risk of death during the follow-up period decreased by 17% for men and 13% for women, regardless of their BMI.^48^ As such, the reductions in waist circumference we observed are clinically meaningful. Blood pressure was lowered across all groups on average. A meta-analysis of randomised trials of blood pressure lowering interventions found that those without cardiovascular disease reduced their risk of having a major cardiovascular event by 9% with a 5 mmHg reduction of systolic blood pressure, even with normal or high–normal blood pressure at baseline.^49^ This reduction is at the lower end of the average decrease in systolic blood pressure observed in the three SWIFt interventions and an important outcome, as shift work is associated with an increased relative risk of developing CVD.^3^ Similar to the findings from the secondary analyses of weight and HOMA-IR, the IF:2D intervention also showed significant mean reductions in blood glucose, total cholesterol, and LDL cholesterol at 24 weeks; possibly a consequence of the greater absolute weight loss observed in the IF:2D group.

### Strengths and limitations

Our study provides evidence of three dietary strategies that can be adopted into the lifestyle of night shift working populations with the aim of weight loss. Each of the three interventions resulted in, on average, a similar reduction in energy intake and we saw no evidence that either modified IF intervention negatively impacted quality of life. This is the largest study to date examining weight loss strategies for night shift workers. Strengths of the study include enrolment of a representative sample of shift workers, a sufficiently long duration of intervention, and collection of a wide range of secondary outcomes that provides an opportunity to inform new research questions. The focus on night shift workers with obesity/overweight is undoubtedly another strength based on the high proportion, 13%–27% of the workforce, in night shift work roles and their increased risk of developing non-communicable diseases.^50^ Limitations include a smaller than proposed sample size and missing outcome data for unsuccessful blood draws. Furthermore, some participants in the IF:2D group may have had a prolonged period of fasting prior to their fasting blood draw. The small, and clinically unimportant, between-group differences observed for the primary outcomes suggest that it is far more likely that there are no between-group differences for the co-primary outcomes, rather than there being any clinically important between-group differences that the study did not have sufficient statistical power to detect. Yet it is important to note that, across all interventions, clinically relevant improvements were consistently observed in a range of metabolic health markers, suggesting the study had sufficient statistical power to identify within-group change. Adherence to the study was assessed using seven-day food diaries, which may be subject to reporting bias such as under-reporting. However, dietary data was verified with each participant by the research dietitians and the reduction in energy intake observed at 24 weeks is consistent with the proposed energy reduction of 20% the study set out to achieve. The use of biomarkers to assess adherence to dietary prescription offer a more objective measure than self-reported dietary intake,^51^ but have not been validated in IF protocols, where dietary (nutrient) intake is less prescribed, relying more on periods of intermittent fasting and feeding to achieve health benefits. Regardless, the improvements in weight and metabolic health outcomes observed are consistent with energy reduction. The decision was made to compare IF groups to CER, an established strategy for weight loss, and as such a ‘non-intervention’ control arm was not included. Whilst a range of shift-working occupations were included in the SWIFt study to maximise diversity of participants, our results may not be generalisable to all demographic groups or to other shift working schedules i.e., schedules excluding overnight. The provision of food may have encouraged adherence in this tightly controlled study, but this would not be common in the workplace. Furthermore, limited variety of food choice may not be reflective of usual dietary patterns.

### Conclusion

This large, randomised trial shows that moderate calorie restriction led to weight loss in night shift workers. IF did not lead to additional improvements in insulin resistance compared with CER. Exploratory analysis of secondary outcomes suggests future research to determine potential cardiometabolic health benefits. The differing approaches to weight loss had similar retention, were effective, safe and provide options for this at-risk population, whilst supporting the growing body of evidence indicating IF as a viable alternative to CER.

## Contributors

MPB: conceptualisation, funding acquisition, methodology, supervision, project administration, accessed and verified the underlying data, writing – original draft, writing – review and editing. GKWL: investigation, methodology, project administration, writing – review and editing. MR: investigation, methodology, project administration, writing – review and editing. RD: investigation, methodology, project administration, data curation, visualisation, accessed and verified underlying data, writing – review and editing. ABC: investigation, methodology, project administration, writing – review and editing. CD: investigation, methodology, project administration, writing – review and editing. YYP: investigation, methodology, project administration, writing – review and editing. RSW: data curation, validation, visualisation, formal analysis, accessed and verified underlying data, writing – review and editing. AE-H: data curation, validation, visualisation, formal analysis, accessed and verified underlying data, writing – review and editing. NJK: conceptualisation, funding acquisition, methodology, writing – review and editing. JD: conceptualisation, funding acquisition, methodology, writing – review and editing. SB: conceptualisation, funding acquisition, methodology, writing – review and editing. TLS: conceptualisation, funding acquisition, methodology, writing – review and editing. CEH: conceptualisation, funding acquisition, methodology, writing – review and editing. AMC: conceptualisation, funding acquisition, methodology, project administration, supervision, writing – review and editing. All authors read and approved the final version of the manuscript.

## Data sharing statement

De-anonymised patient level data with low risk of re-identification are available upon reasonable request from the corresponding author, after receipt of a methodologically sound proposal and approval on a case-by-case basis at the discretion of the primary sponsor.

## Declaration of interests

All authors have completed the ICMJE uniform disclosure form at http://www.icmje.org/disclosure-of-interest/ and declare: no support from any organisation for the submitted work; TLS is a consultant for Vanda Pharmaceuticals, unrelated to this work; no other relationships or activities that could appear to have influenced the submitted work.
