# Supplementary material for: Intermittent fasting for weight loss in night shift workers: a three-arm, superiority randomised clinical trial
Source: eBioMedicine. 2025 Jun 18;117:105803. doi: 10.1016/j.ebiom.2025.105803 (PMC12219368; doi:10.1016/j.ebiom.2025.105803)
Supplement: Supplementary Materials [file mmc1.pdf]

**Intermittent fasting for weight loss in night shift workers: A three-arm, randomised clinical trial**

**Supplementary Material**

# Table of Contents

**Supplementary methods** .......... **2**

COVID-19 infection history .......... 2

**Supplementary Tables** .......... **3**

Table S1: Comparison between completers and non-completers (N=248) .......... 3

Table S2: Within-group changes in body composition and cardiometabolic risk factors over 24-week intervention period .......... 4

Table S3. The effect of group allocation on primary outcome measures during the 24-week intervention: sensitivity analyses .......... 5

Table S4: Comparison of outcomes between groups at each time point and use of a multiple imputation approach for missing data (N=248) .......... 6

Table S5: Number of individuals reporting common adverse events believed associated with the interventions .......... 7

Table S6: Summary statistics for primary and secondary outcomes at baseline,12, and 24 weeks .......... 8

**Supplementary Figures** .......... **10**

Supplementary Figure S1: Effect of dietary interventions on body mass index and waist circumference for completers .......... 10

Supplementary Figure S2: Effect of dietary interventions on blood pressure and lipids for completers. .......... 11

**Protocol** .......... **12**

**Statistical Analysis Plan** .......... **42**

**COVID-19 infection history**

After the start of the COVID-19 pandemic, self-reported information relating to recent COVID-19 infection was collected at each in-person clinic visit. It was a requirement that participants not attend the in-person clinic visit if they had tested positive to COVID-19. A total of 11 people reported at their 24 week in-person clinic visit that they had tested positive to COVID-19 since they last attended (one person reported this at their 12 week visit). No one had required medical attention for their infection. Any participants recruited pre- and post the start of the COVID-19 pandemic would have to have met the study inclusion criteria of a minimum of 4 night shifts per fortnight and as such were not as impacted by stay at home mandates.

## Supplementary tables

**Table S1: Comparison between completers and non-completers (N=248).**

| Variable | Completers (n=170) | Non-completers (n=78) | p-value |
|---|---|---|---|
| Gender (female), No (%) | 84 (49.4) | 49 (61.3) | 0.08 |
| Age, mean (SD), y | 47.4 (9.3) | 45.4 (10.6) | 0.15 |
| Body weight, mean (SD), kg | 98.5 (18.8) | 106.3 (22.4) | 0.004 |
| BMI, mean (SD), kg/m$^2$ | 33.7 (4.8) | 37.3 (7.3) | <0.001 |
| Fat mass, mean (SD), kg | 38.4 (9.5) | 44.9 (1.6) | <0.001 |
| Fat-free mass, mean (SD), kg | 56.9 (12.8) | 56.2 (11.4) | 0.72 |
| Energy intake, mean (SD), kJ | 8754.2 (2611.9) | 8448.9 (3175.5) | 0.44 |
| Waist circumference, mean (SD), cm | 103.2 (14.7) | 108.9 (17.3) | 0.01 |
| Systolic blood pressure, mean (SD), mm Hg | 130.8 (16.4) | 130.4 (16.5) | 0.83 |
| Diastolic blood pressure, mean (SD), mm Hg | 83 (9.0) | 84 (9.6) | 0.42 |
| Fasting insulin, mean (SD), mIU/L | 11.6 (6.8) | 13.5 (7.7) | 0.06 |
| Fasting glucose, mean (SD), mg/dL | 97.3 (21.6) | 99.1 (21.6) | 0.60 |
| HOMA-IR[a], mean (SD) | 3.5 (2.7) | 4.0 (2.9) | 0.07 |
| HbA1c (%) | 5.4 (0.8) | 5.5 (0.7) | 0.38 |
| Triglycerides, mean (SD), mg/dL | 141.6 (97.4) | 123.9 (79.7) | 0.12 |
| Total Cholesterol, mean (SD), mg/dL | 223.9 (42.5) | 220.1 (42.5) | 0.51 |
| LDL-Cholesterol, mean (SD), mg/dL | 139.0 (34.8) | 139.3 (34.8) | 0.73 |
| HDL-Cholesterol, mean (SD), mg/dL | 54.1 (15.6) | 54.1 (11.6) | 0.79 |
| Sleep episode duration on night shifts, mean (SD), hr | 6.3 (1.6) | 6.1 (1.9) | 0.54 |
| Sleep episode duration on days off, mean (SD), hr | 8.8 (1.2) | 8.8 (1.3) | 0.81 |
| Quality of life (AQOL-8D)[b], mean (SD) | 0.72 (0.18) | 0.66 (0.15) | 0.03 |

Abbreviations: BMI, body mass index; CER, continuous energy restriction; CI, confidence interval; HDL, high-density lipoprotein; HOMA-IR, homeostatic model assessment for insulin resistance; IQR, interquartile range, LDL, low-density lipoprotein; SD, standard deviation.

Missing items: Completers, fat mass and fat-free mass n=3, glucose/insulin/HOMA/HbA1c n=8; triglycerides and cholesterol n=9, sleep on night shift n=21 , sleep on days off n=26, Non-completers, fat mass and fat-free mass n=5, waist circumference n=6, energy intake n=5, blood pressure n=5, glucose/insulin/HOMA/HbA1c n=14, triglycerides, and cholesterol n=12, sleep on night shift n=10, sleep on days off n=18.

[a] HOMA-IR= fasting insulin (mIU/L) x fasting glucose (mmol/L)/22.5.

[b] AQOL-8D; Weighted utility score (Dimension Worst Health State = 0.00 - Dimension Best Health State = 1.00)

**Table S2: Within-group changes in body composition and cardiometabolic risk factors over 24-week study period[a]**

| At baseline:<br>At 24-weeks | CER<br>(N=84)<br>(N=58) | IF:2D<br>(N=83)<br>(N=53) | IF:2N<br>(N=81)<br>(N=59) | CER | IF:2D | IF:2N |
|---|---|---|---|---|---|---|
| **Variable** | **Mean (SD)** | **Mean (SD)** | **Mean (SD)** | **Mean difference (95% CI)** | **Mean difference (95% CI)** | **Mean difference (95% CI)** |
| **Primary outcomes** | | | | | | |
| Body weight, kg | 91.2 (15.2) | 93.9 (20.4) | 91.4 (18.7) | **-5.4 (-6.4 to -4.4)** | **-8.5 (-9.8 to -7.2)** | **-5.5 (-6.7 to -4.2)** |
| HOMA_IR[b] | 2.5 (1.3) | 2.4 (1.3) | 2.8 (2.7) | **-0.4 (-0.7 to -0.1)** | **-0.9 (-1.3 to -0.58)** | **-0.9 (-1.4 to -0.4)** |
| **Secondary outcomes** | | | | | | |
| BMI, kg/m$^2$ | 31.4 (4.5) | 31.0 (5.2) | 31.9 (4.6) | **-1.8 (-2.2 to -1.4)** | **-2.8 (-3.2 to -2.4)** | **-1.9 (-2.3 to -1.5)** |
| Fat mass, kg | 34.2 (10.1) | 31.6 (9.1) | 36.0 (9.5) | **-4.1 (-5.5 to -2.8)** | **-5.7 (-6.9 to -4.3)** | **-3.9 (-5.5 to -2.4)** |
| Fat-free mass, kg | 55.1 (11.2) | 58.3 (12.9) | 54.2 (13.1) | **-1.2 (-1.6 to -0.7)** | **-1.6 (-2.1 to 1.1)** | **-1.2 (-1.7 to -0.7)** |
| Waist circumference, cm | 97.8 (12.8) | 95.8 (14.9) | 95.2 (15.3) | **-4.2 (-5.5 to -3.3)** | **-7.4 (-9.1 to -5.6)** | **-6.2 (-8.2 to -4.3)** |
| Energy intake, Kj | 6794.5 (1946.0) | 6773.8 (1667.5) | 6260.6 (1780.6) | **-1846.7 (-2434.5 to -1259.0)** | **-2454.3 (-3180.1 to -1728.3)** | **-2018.9 (-2577.9 to -1459.7)** |
| Systolic blood pressure, mm Hg | 125.8 (13.3) | 123.9 (15.1) | 123.2 (15.1) | **-4.1 (-6.7 to -1.5)** | **-7.4 (-11.9 to -2.8)** | **-4.1 (-6.9 to -1.2)** |
| Diastolic blood pressure, mm Hg | 81.1 (6.8) | 80.4 (7.9) | 80.2 (8.0) | **-4.2 (-5.7 to -2.6)** | **-2.3 (-4.6 to -0.1)** | **-2.4 (-4.2 to -0.7)** |
| Fasting glucose, mg/dL | 93.7 (7.2) | 90.1 (7.2) | 95.5 (12.6) | -1.8 (-3.6 to 0.0) | **-3.6 (-5.4 to -1.8)** | -1.8 (-3.6 to 1.8) |
| Fasting insulin, mIU/L | 10.5 (5.2) | 10.5 (5.7) | 11.4 (7.8) | **-1.6 (-2.7 to -0.2)** | **-3.1 (-4.3 to -1.9)** | **-3.7 (-5.1 to -2.3)** |
| HbA1c (%) | 5.4 (0.3) | 5.2 (0.4) | 5.4 (0.5) | 0.01 (-0.05 to 0.07) | -0.02 (-0.07 to 0.04) | 0.0 (-0.06 to 0.07) |
| Triglycerides, mg/dL | 150.4 (88.5) | 130.7 (79.7) | 135.4 (106.2) | 25.8 (0.0 to 49.1) | 1.0 (-35.1 to 23.6) | -27.6 (-70.8 to 8.9) |
| Total Cholesterol, mg/dL | 223.0 (46.3) | 204.6 (34.8) | 218.0 (42.5) | 0.9 (-6.7 to 8.0) | **-18.8 (-22.0 to -11.6)** | -1.0 (-11.6 to 3.1) |
| LDL-Cholesterol, mg/dL | 142.7 (34.8) | 126.1 (34.8) | 136.6 (34.8) | 3.4 (-5.7 to 9.0) | **-14.9 (-20.2 to -8.0)** | -0.4 (-7.0 to 6.7) |
| HDL-Cholesterol, (mg/dL) | 53.6 (14.1) | 55.6 (12.1) | 58.8 (17.4) | 0.9 (-0.4 to 3.9) | -1.0 (-3.0 to 1.9) | 1.5 (-0.1 to 3.7) |
| Quality of life (AQOL-8D)[c] | 0.73 (0.14) | 0.74 (0.17) | 0.76 (0.16) | 0.02 (-0.01 to 0.04) | **0.05 (0.02 to 0.07)** | 0.01 (-.02 to 0.05) |
| Sleep episode duration (night shifts), hr | 6.6 (1.6) | 6.0 (1.6) | 6.5 (1.9) | 0.4 (0.0 to 0.8) | 0.3 (0.0 to 0.6) | -0.3 (-0.1 to 0.7) |
| Sleep episode duration (days off), hr | 8.9 (0.9) | 9.1 (1.3) | 8.8 (1.3) | 0.0 (-0.3 to 0.3) | 0.1 (-0.3 to 0.4) | -0.2 (-0.4 to 0.1) |
| Physical activity[d,e] , total MET, Median (IQR) , min/w | 6798 (3060 to 11862) | 5016 (2484 to 8088) | 7204 (3252 to 15270) | 232.5 (-1121.2 to 1564.2) | 12 (-2433.5 to 2457.5) | 27 (-3961.0 to 4015.0) |

Abbreviations: BMI, body mass index; CER, continuous energy restriction; CI, confidence interval; HDL, high-density lipoprotein; HOMA-IR, homeostatic model assessment for insulin resistance; IQR, interquartile range, LDL, low-density lipoprotein; SD, standard deviation. The value in **bold** indicates a statistically significant result at a threshold of $p<0.05$.

[a] Analyses were conducted with the use of a linear mixed-effects model,

[b] HOMA-IR= fasting insulin (mIU/L) x fasting glucose (mmol/L)/22.5.

[c] AQOL-8D; Weighted utility score (Dimension Worst Health State = 0.00 - Dimension Best Health State = 1.00)

[d] Data from International Physical Activity Questionnaire (IPAQ)-Long Form which measures self-reported time spent being physically active across multiple domains: transportation, recreation (including sport and leisure time), housework, job-related and time spent sitting

[e] Median regression analysis was used

**Table S3. The effect of group allocation on primary outcome measures during the 24-week intervention: sensitivity analyses[a].**

| Variable | Number | CER | IF:2D | IF:2N | IF:2D vs CER | IF:2N vs CER |
|---|---|---|---|---|---|---|
| | Participants (Observations) | Mean (SD) | Mean (SD) | Mean (SD) | Mean difference (95% CI) | Mean difference (95% CI) |
| **Body Weight** | | | | | | |
| Baseline values | 248 (248) | 100.5 (19.8) | 102.8 (21.9) | 99.7 (19.3) | | |
| 24-week values | 170 (170) | 91.2 (15.2) | 93.9 (20.4) | 91.4 (18.7) | | |
| Standard model | 248 (609) | | | | -0.2 (-6.4 to 5.9) | -0.6 (-6.8 to 5.6) |
| Model adjusted for stratification factors[b] | 248 (609) | | | | -0.2 (-6.3 to 6.0) | -0.5 (-6.7 to 5.7) |
| Model adjusted for baseline value | 170 (332) | | | | **-2.7 (-4.2 to -1.2)** | 0.1 (-1.4 to 1.5) |
| Model adjusted for factors associated with attrition[c] | 170 (332) | | | | **-1.8 (-3.2 to -4.1)** | 0.3 (-1.1 to 1.6) |
| Model with home scales[d] | 248 (1,763) | | | | -0.1 (-6.3 to 6.1) | -0.6 (-6.8 to 5.6) |
| *Completers model* | | | | | | |
| Baseline values | 170 (170) | 96.6 (19.1) | 102.4 (21.1) | 96.9 (19.1) | | |
| 24-week values | 170 (170) | 91.2 (15.2) | 93.9 (20.4) | 91.4 (18.7) | 2.7 (-4.0 to 9.5) | 0.3 (-6.4 to 6.9) |
| **HOMA-IR**[e] | | | | | | |
| Baseline values | 221 (221) | 3.2 (2.5) | 3.6 (2.8) | 4.0 (2.7) | | |
| 24-week values | 147 (147) | 2.5 (1.3) | 2.4 (1.3) | 2.8 (2.7) | | |
| Standard model | 234 (511) | | | | -0.1 (-1.0 to 0.8) | 0.3 (-0.5 to 1.2) |
| Model adjusted for stratification factors[b] | 234 (511) | | | | -0.1 (-1.0 to 0.8) | 0.3 (-0.6 to 1.2) |
| Model adjusted for baseline value | 147 (272) | | | | -0.4 (-0.9 to 0.1) | -0.4 (-0.9 to 0.1) |
| Model adjusted for factors associated with attrition[c] | 147 (272) | | | | 0.1 (-0.6 to 0.8) | 0.5 (-0.1 to 1.3) |
| *Completers model* | | | | | | |
| Baseline values | 159 (159) | 3.1 (2.3) | 3.5 (2.8) | 3.9 (2.9) | | |
| 24-week values | 147 (147) | 2.5 (1.3) | 2.4 (1.3) | 2.8 (2.7) | 0.0 (-0.9 to 0.9) | 0.3 (-0.6 to 1.3) |

Abbreviations: CER, continuous energy restriction; CI, confidence interval; HOMA-IR, homeostatic model assessment for insulin resistance; SD, standard deviation. The value in **bold** indicates a statistically significant result at a threshold of p<0.05.

[a] Analyses were conducted with the use of a linear mixed-effects model,

[b] Adjusted for study site and age of participants,

[c] Adjusted for baseline values of weight, Fat mass, BMI, Waist circumference and Quality of life.

[d] Model included additional weight measures collected at home using standardised Bluetooth weighing scales provided to all participants.

[e] HOMA-IR= fasting insulin (mIU/L) x fasting glucose (mmol/L)/22.5

**Table S4: Comparison of outcomes between groups at each time point and use of a multiple imputation approach for missing data (N=248)**

| Characteristics | CER | IF:2D | IF:2N | IF:2D vs CER | IF:2N vs CER |
|---|---|---|---|---|---|
| **At 24 weeks** | **Mean (SD)** | **Mean (SD)** | **Mean (SD)** | **Mean difference (95% CI)** | **Mean difference (95% CI)** |
| **Primary outcome** | | | | | |
| Body weight, Kg | 93.4 (19.8) | 91.6 (19.7) | 93.6 (18.9) | 1.4 (-5.3 to 8.1) | 0.3 (-6.5 to 7.0) |
| HOMA-IR[a] | 3.2 (2.3) | 2.9 (2.5) | 3.5 (2.4) | -0.1 (-0.8 to 0.7) | 0.4 (-0.4 to 1.1) |
| **Secondary outcome** | | | | | |
| Waist circumference, cm | 102.4 (15.1) | 100.4 (17.4) | 99.0 (15.6) | -1.9 (-6.9 to 2.9) | -2.5 (-7.5 to 2.4) |
| BMI, kg/m2 | 32.0 (7.5) | 32.1 (9.2) | 32.4 (7.6) | -0.3 (-2.7 to 2.1) | -0.1 (-2.5 to 2.3) |
| Fat mass, kg | 35.8 (10.4) | 35.2 (11.0) | 34.7 (10.7) | -0.7 (-4.5 to 3.1) | -0.9 (-4.7 to 4.2) |
| Fat-free mass, kg | 54.6 (13.3) | 55.2 (14.3) | 56.1 (11.2) | 1.9 (-2.1 to 6.0) | 0.3 (-3.8 to 4.3) |
| Fasting glucose, mg/dL | 97.3 (14.4) | 97.3 (23.4) | 97.3 (19.8) | -0.7 -6.8 to 5.4) | 1.4 (-4.6 to 7.2) |
| Fasting insulin, mIU/L | 12.6 (8.1) | 11.5 (7.1) | 14.1 (7.8) | -0.3 (-2.8 to 2.2) | 1.0 (-1.4 to 3.5) |
| HbA1c (%) | 5.4 (0.4) | 5.3 (0.4) | 5.4 (0.3) | -0.06 (-0.25 to 0.13) | 0.0 (-0.19 to 0.20) |
| Triglycerides, mg/dL | 141.6 (97.4) | 125.7 (97.4) | 140.8 (97.4) | -17.2 (-52.7 to 25.6) | -8.2 (-46.3 to 27.3) |
| Total Cholesterol, mg/dL | 220.1 (42.5) | 208.5 (46.3) | 212.4 (46.3) | **-15.9 (-30.7 to -3.9)** | -3.9 (-19.3 to 7.7) |
| LDL-Cholesterol, mg/dL | 142.9 (34.8) | 127.4 (34.8) | 139.0 (38.6) | **-14.3 (-26.0 to -1.5)** | -2.8 (-14.4 to 5.7) |
| HDL-Cholesterol, mg/dL | 54.8 (15.4) | 55.5 (15.4) | 50.2 (11.6) | 1.4 (-3.9 to 7.7) | 1.9 (-3.9 to 7.7) |
| Systolic Blood pressure, mm Hg | 125 (14) | 125 (16) | 124 (2) | -1.2 (-6.4 to 4.0) | -1.7 (-6.9 to 3.4) |
| Diastolic Blood pressure, mm Hg | 81 (8) | 80 (9) | 81 (7) | -0.8 (-3.6 to 2.0) | -0.8 (-3.6 to 1.9) |
| Quality of life (AQOL-8D)[b] | 0.67 (0.17) | 0.74 (0.16) | 0.69 (0.16) | 0.03 (-0.02 to 0.08) | 0.03 (-0.02 to 0.09) |
| Sleep episode duration on night shifts, hr | 6.3 (1.9) | 6.4 (1.8) | 6.5 (1.9) | -0.3 (-0.9 to 0.3) | -0.1 (-0.7 to 0.5) |
| Sleep episode duration on days off, hr | 8.7 (1.3) | 8.8 (1.4) | 8.7 (1.3) | 0.1 (-0.4 to 0.5) | -0.1 (-0.6 to 0.4) |
| Physical activity, total MET min / w[c,d] | 7709.9 (4018.0 to 12700.9) | 7560.0 (3584 to 12488.4) | 8742.2 (4730.5 to 13861.5) | -1602.0 (-3521.0 to 317.0) | 606.0 (-1272.9 to 2484.9) |

Abbreviations: AQOL-8D, Assessment of Quality of Life-8 Dimension; BMI, body mass index; CER, continuous energy restriction; CI, confidence interval; HDL, high-density lipoprotein; HOMA-IR, homeostatic model assessment for insulin resistance; IQR, interquartile range, LDL, low-density lipoprotein; MET, metabolic equivalent of task; SD, standard deviation. The value in **bold** indicates a statistically significant result.

[a] HOMA-IR= fasting insulin (mIU/L) x fasting glucose (mmol/L)/22.5.

[b] AQOL-8D; Weighted utility score (Dimension Worst Health State = 0.00 - Dimension Best Health State = 1.00)

[c] Data from International Physical Activity Questionnaire (IPAQ)-Long Form.

[d] Median regression analysis was used

**Table S5: Number of individuals reporting common adverse events (AE) believed associated with the interventions.**

| | **IF:2D (n = 83)** | **IF:2N (n=81)** | **CER (n = 84)** | IF:2D vs CER | IF:2N vs CER |
|---|---|---|---|---|---|
| | No. (%) | No. (%) | No. (%) | OR (95% CI); P-value | OR (95% CI); P-value |
| Headache | 16 (19) | 18 (22) | 0 (0) | 27.7 (4.6 – Inf); <0.001 | 33.1 (5.5 – Inf); <0.001 |
| Gastrointestinal symptoms[a] | 6 (7) | 7 (9) | 0 (0) | 8.7 (1.2 – Inf); 0.03 | 11.0 (1.6 – Inf); 0.01 |
| Light-headed | 4 (5) | 4 (5) | 2 (2) | 2.1 (0.3 – 23.5); 0.67 | 2.1 (0.3 – 24.1); 0.65 |
| Fatigue | 3 (4) | 6 (7) | 1 (1) | 3.1 (0.2 – 165.3); 0.61 | 6.6 (0.8 – 309.0); 0.11 |
| Irritable/emotional | 2 (2) | 2 (2) | 3 (4) | 0.7 (0.1 – 6.0); 1.00 | 0.7 (0.1 – 6.2); 1.00 |

Number of participants who experienced AE (participant is to be counted only once for each AE).
[a] including nausea, abdominal pain/cramps, change in bowel habits diarrhea/constipation, flatus
Association between group and adverse event investigated using exact logistic regression

**Table S6: Summary statistics for primary and secondary outcomes at baseline, 12, and 24 weeks.**

| **At baseline:**<br>**At 12-weeks:**<br>**At 24-weeks:** | **CER**<br>**(n=84)**<br>**(n=64)**<br>**(n=58)** | **IF:2D**<br>**(n=83)**<br>**(n=62)**<br>**(n=53)** | **IF:2N**<br>**(n=81)**<br>**(n=66)**<br>**(n=59)** |
|---|---|---|---|
| **Variable** | **Mean (SD)** | **Mean (SD)** | **Mean (SD)** |
| **Primary outcomes** | | | |
| *Body weight, kg* | | | |
| At baseline | 100.4 (19.8) | 102.8 (21.9) | 99.7 (19.3) |
| At 12-weeks | 94.8 (17.8) | 99.2 (22.1) | 94.8 (19.5) |
| At 24-weeks | 91.2 (15.2) | 93.9 (20.4) | 91.4 (18.7) |
| *HOMA_IR* | | | |
| At baseline | 3.4 (2.6) | 3.6 (2.8) | 4.0 (2.7) |
| At 12-weeks | 2.6 (1.6) | 2.9 (2.7) | 3.2 (2.3) |
| At 24-weeks | 2.5 (1.3) | 2.4 (1.3) | 2.8 (2.7) |
| **Secondary outcomes** | | | |
| *BMI, kg/m2* | | | |
| At baseline | 34.8 (5.7) | 35.0 (6.8) | 34.7 (5.2) |
| At 12-weeks | 32.7 (5.3) | 32.8 (6.4) | 32.9 (4.9) |
| At 24-weeks | 31.4 (4.5) | 31.0 (5.2) | 31.9 (4.6) |
| *Fat mass, kg* | | | |
| At baseline | 41.3 (12.8) | 39.9 (10.8) | 39.8 (8.9) |
| At 12-weeks | | | |
| At 24-weeks | 34.2 (10.1) | 31.6 (9.1) | 36.0 (9.5) |
| *Fat-free mass, kg* | | | |
| At baseline | 56.5 (11.8) | 58.1 (12.6) | 55.4 (12.6) |
| At 12-weeks | - | - | - |
| At 24-weeks | 55.1 (11.2) | 58.3 (12.9) | 54.2 (13.1) |
| *HbA1c (%)* | | | |
| At baseline | 5.4 (0.5) | 5.5 (0.9) | 5.5 (0.7) |
| At 12-weeks | 5.3 (0.3) | 5.3 (0.9) | 5.5 (0.6) |
| At 24-weeks | 5.4 (0.3) | 5.2 (0.4) | 5.4 (0.5) |
| *Waist circumference, cm* | | | |
| At baseline | 105.0 (14.9) | 105.1 (16.8) | 104.5 (15.7) |
| At 12-weeks | 98.7 (9.8) | 100.9 (14.5) | 100.5 (13.6) |
| At 24-weeks | 97.8 (12.8) | 95.8 (14.9) | 95.2 (15.3) |
| *Energy intake, kJ* | | | |
| At baseline | 8732.6 (2912.1) | 8866.4 (2891.8) | 8384.1 (2560.1) |
| At 12-weeks | - | - | - |
| At 24-weeks | 6794.5 (1946.0) | 6773.8 (1667.5) | 6260.6 (1780.6) |
| *Systolic blood pressure, mm Hg* | | | |
| At baseline | 132.5 (16.8) | 131.5 (17.7) | 127.8 (14.7) |
| At 12-weeks | 126.9 (15.6) | 127.5 (16.4) | 123.5 (13.5) |
| At 24-weeks | 125.8 (13.3) | 123.9 (15.1) | 123.2 (15.1) |
| *Diastolic blood pressure, mm Hg* | | | |
| At baseline | 85.1 (8.5) | 83.0 (10.1) | 82.7 (8.7) |
| At 12-weeks | 82.3 (8.9) | 81.1 (9.2) | 80.3 (8.4) |
| At 24-weeks | 81.1 (6.8) | 80.4 (7.9) | 80.2 (8.0) |
| *Fasting glucose, mg/dL* | | | |
| At baseline | 97.3 (14.4) | 99.1 (28.8) | 99.1 (19.8) |
| At 12-weeks | 93.7 (9.9) | 93.9 (26.9) | 96.3 (20.1) |
| At 24-weeks | 93.7 (7.2) | 90.1 (7.2) | 95.5 (12.6) |
| *Fasting insulin, mIU/L* | | | |
| At baseline | 13.4 (8.3) | 14.2 (8.2) | 15.9 (8.5) |
| At 12-weeks | 11.4 (7.2) | 11.8 (7.5) | 13.8 (8.6) |
| At 24-weeks | 10.5 (5.12) | 10.4 (5.6) | 11.3 (7.8) |
| *Triglycerides, mg/dL* | | | |
| At baseline | 132.7 (79.6) | 130.2 (81.6) | 153.4 (123.9) |
| At 12-weeks | 119.1 (51.9) | 122.3 (99.2) | 150.2 (99.7) |
| At 24-weeks | 150.4 (88.5) | 130.7 (79.7) | 135.4 (106.2) |
| *Total Cholesterol, mg/dL* | | | |
| At baseline | 225.1 (42.5) | 219.0 (41.5) | 220.1 (42.5) |

| | | | |
|---|---|---|---|
| At 12-weeks | 215.2 (46.3) | 208.0 (36.4) | 220.1 (41.7) |
| At 24-weeks | 223.0 (46.3) | 204.6 (34.8) | 218.0 (42.5) |
| *LDL-Cholesterol, mg/dL* | | | |
| At baseline | 143.2 (33.1) | 137.3 (34.8) | 137.7 (34.8) |
| At 12-weeks | 134.7 (33.5) | 127.6 (31.3) | 138.0 (37.6) |
| At 24-weeks | 142.7 (34.8) | 126.1 (34.8) | 136.6 (34.8) |
| *HDL-Cholesterol, mg/dL* | | | |
| At baseline | 53.5 (14.2) | 54.5 (15.4) | 54.5 (15.4) |
| At 12-weeks | 53.1 (14.2) | 53.3 (12.3) | 57.3 (15.7) |
| At 24-weeks | 53.6 (14.1) | 55.6 (12.1) | 58.8 (17.4) |
| *Quality of life (AQOL-8D)* | | | |
| At baseline | 0.68 (0.16) | 0.70 (0.16) | 0.72 (0.16) |
| At 12-weeks | - | - | - |
| At 24-weeks | 0.73 (0.14) | 0.74 (0.17) | 0.76 (0.16) |
| *Sleep episode duration on night shifts, hrs* | | | |
| At baseline | 6.2 (1.9) | 5.9 (1.8) | 6.2 (1.6) |
| At 12-weeks | - | - | - |
| At 24-weeks | 6.6 (1.6) | 6.0 (1.6) | 6.5 (1.9) |
| *Sleep episode duration on days off, hrs* | | | |
| At baseline | 8.8 (1.3) | 8.9 (1.5) | 8.9 (1.3) |
| At 12-weeks | - | - | - |
| At 24-weeks | 8.9 (0.9) | 9.1 (1.3) | 8.8 (1.3) |
| *Physical activity total MET, Median (IQR), min/w* | | | |
| At baseline | 6413 (2919 to 11346) | 5052 (2228 to 9443) | 7540 (3108 to 12207) |
| At 12-weeks | - | - | - |
| At 24-weeks | 6798 (3060 to 11862) | 5016 (2484 to 8088) | 7204 (3252 to 12207) |

Abbreviations: AQOL-8D, Assessment of Quality of Life-8 Dimension; BMI, body mass index; CER, continuous energy restriction; CI, confidence interval; HDL, high-density lipoprotein; HOMA-IR, homeostatic model assessment for insulin resistance; IQR, interquartile range, LDL, low-density lipoprotein; MET, metabolic equivalent of task; SD, standard deviation.

Missing items for data collected at baseline, 12- and 24-weeks: CER: weight and BMI, 0,19,26, waist circumference n=2,40,27, blood pressure n=1,13,27, glucose/insulin/HOMA/HbA1c n=11,22,32 triglycerides, and cholesterol n=8,20,30, IF:2D weight and BMI, 0,22,30, waist circumference n=1,38,35, HbA1c n=7,15,8, blood pressure n=3,15,35, glucose/HOMA n=7,15,39; insulin/HbA1c n=6,14,38; triglycerides and cholesterol n=7,14,39; IF:2N: weight and BMI, 0,16,22, waist circumference n=3,41,25, HbA1c n=5,13,6, blood pressure n=1,17,25, glucose/HOMA/HbA1c n=9,13,28; insulin n=8,14,29; triglycerides and cholesterol n=6,14,28.
Missing items for data collected at baseline and 24-weeks: CER: fat mass and fat-free mass n=3,29, energy intake n=3,29, Quality of life, 0,26, sleep on night shift n= 12,33, sleep on days off n=17,35 , physical activity n=3,26; 5:2 Day: fat mass and fat-free mass n=2,35, energy intake n=2,40, Quality of life, 0,33, sleep on night shift n= 8,38, sleep on days off n=12,44, physical activity n=3,33; 5:2 Night: fat mass and fat-free mass n=3,30, energy intake n=1,30, Quality of life, 0,23, sleep on night shift n= 11,33, sleep on days off n=15,31, physical activity n=0,23

## Supplementary figures

**Supplementary Figure S1: Effect of dietary interventions on body mass index and waist circumference for completers (graph)**

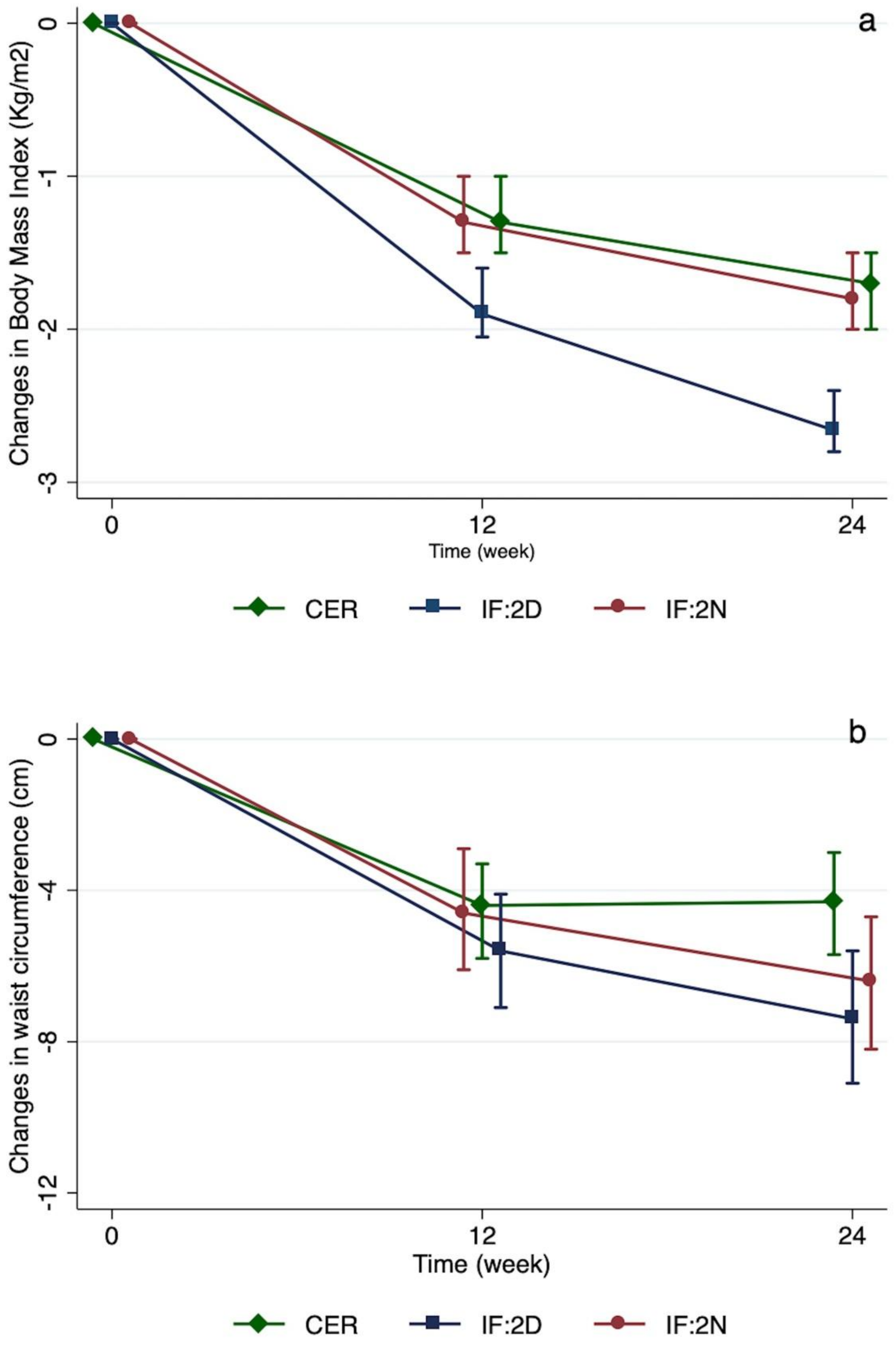


**a) Body mass index.; b) Waist circumference.** Note the differing scales and minimum values on the Y-axes. Point estimates and error bars are offset for clarity.

**Supplementary Figure S2: Effect of dietary interventions on blood pressure and lipids for completers.**

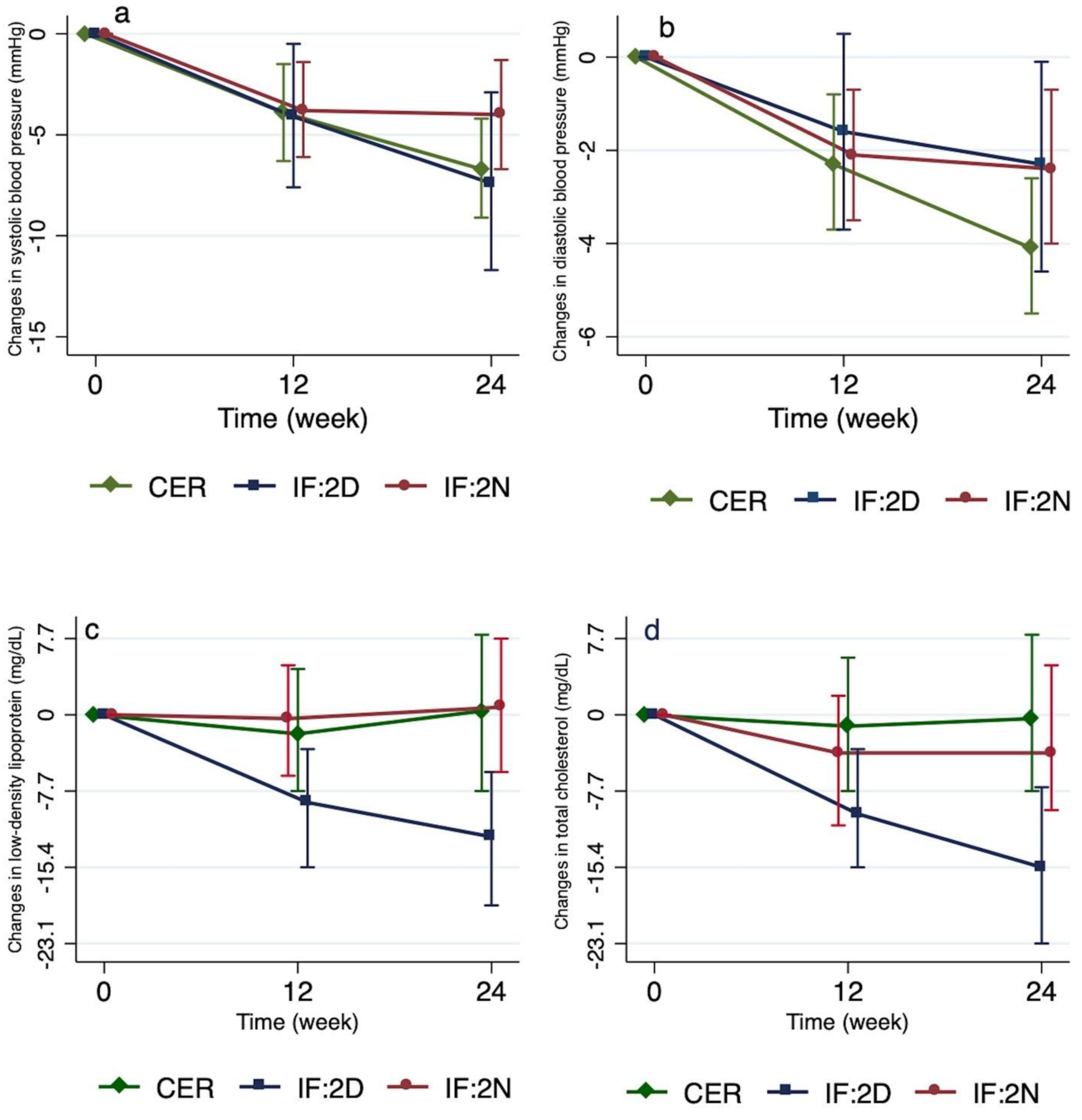


**a) Systolic blood pressure; b) Diastolic blood pressure; c) Low-density lipoprotein; d) Total cholesterol.** Note the differing scales and minimum values on the Y-axes. Point estimates and error bars are offset for clarity.

## Protocol

Version 10, December 13, 2022 (protocol with final amendments, approval of main protocol was dated August 8, 2019)

**Protocol Title: Shifting Weight using Intermittent Fasting in night shift workers (SWIFt) study: a three-arm randomised controlled trial comparing three weight loss strategies in night shift workers with obesity**

**Trial Registration:** ANZCTR: Identifier: ACTRN-12619001035112, https://www.anzctr.org.au

**Funding:** This project is funded by the National Health Medical Research Council (APP1159762).

**Primary Investigator:**

**Associate Professor Maxine Bonham**
Department of Nutrition, Dietetics and Food,
Be Active Sleep Eat Facility,
Monash University, Level 1, 264 Ferntree Gully Road
Notting Hill, VIC, 3168, Australia, Ph: +61 3 9902 4272
Email: maxine.bonham@monash.edu

**Co-Investigators:**

**Dr Catherine Huggins**
Department of Nutrition, Dietetics and Food,
Monash University

**Dr Nicole Kellow**
Department of Nutrition, Dietetics and Food,
Monash University

**Dr Tracey Sletten**
Turner Institute for Brain and Mental Health,
Monash University

**Professor Siobhan Banks**
Behaviour-Brain-Body Research Centre (BBB),
University of South Australia

**Professor Alison Coates**
Alliance for Research in Exercise, Nutrition and Activity (ARENA),
University of South Australia

**Professor Jillian Dorrian**
Behaviour-Brain-Body Research Centre (BBB),
University of South Australia

**Dr Michelle Rogers**
Alliance for Research in Exercise, Nutrition and Activity (ARENA),
University of South Australia

**Dr Gloria Leung,**
Department of Nutrition, Dietetics and Food,
Monash University

**Dr Rochelle Davis**
Department of Nutrition, Dietetics and Food,
Monash University

**Angela Clark**
Department of Nutrition, Dietetics and Food,
Monash University

**Corinne Davis**
Department of Nutrition, Dietetics and Food,
Monash University

**Yan Yin Phoi**
Alliance for Research in Exercise, Nutrition and Activity (ARENA),
University of South Australia

**Abbreviations**

| | |
|---|---|
| IF:2D | twice-per-week modified intermittent fasting on day shifts |
| IF:2N | twice-per-week modified intermittent fasting on night shifts |
| AGEs | Advanced glycation end-products |
| BASE | Be Active Sleep Eat |
| BIA | Bio-electrical impedance |
| BMI | Body mass index |
| BNSQ | Basic nordic sleep questionnaire |
| BP | Blood pressure |
| CER | Continuous energy restriction |
| CI | Chief investigator |
| CRF | Case report form |
| CVD | Cardiovascular disease |
| DASS-21 | Depression, anxiety and stress scale 21 |
| DOI | Digital object identifier |
| DR | Dietary restriction |
| DXA | Dual x-ray absorptiometry |
| ER | Energy restriction |
| HOMA-IR | Homeostatic model assessment for insulin resistance |
| HREC | Human Research Ethics Committee |
| ICH | International conference on harmonization |
| IF | Intermittent fasting |
| IPAQ | International physical activity questionnaires |
| MUHREC | Monash University Human Research Ethics Committee |
| MVPA | Moderate to vigorous physical activity |
| QOL | Quality of life |
| SAE | Serious adverse event |
| SOS | Survey of shift workers |
| SVM | Signal vector magnitude |
| SWIFt | Shifting Weight using Intermittent Fasting in night shift workers |
| T2D | Type 2 diabetes |
| WC | Waist circumference |

# Table of Contents

1. Background/Significance ........ 16
2. Aims and Hypothesis ........ 16
  2.1 Aims ........ 16
  2.2 Hypotheses ........ 16
3. Trial Design ........ 17
  3.1 Treatment ........ 17
    3.1.1 Diet 1: 5:2 Day fasting diet ........ 18
    3.1.2 Diet 2: 5:2 Night fasting diet ........ 18
    3.1.3 Diet 3: 20% Continuous daily Energy Restriction (20% ER) ........ 18
  3.2 Randomisation ........ 18
  3.3 Blinding ........ 18
  3.4 Definition of a Completed Subject ........ 18
  3.5 Source Data ........ 18
  3.6 Selection and Withdrawal of Subjects ........ 19
    3.6.1 Inclusion Criteria ........ 19
    3.6.2 Exclusion Criteria ........ 19
    3.6.3 Withdrawal Criteria ........ 20
4. Study Assessments and Procedures ........ 21
  4.1 Recruitment ........ 21
    4.1.1 Video recruitment using current participants ........ 21
    4.1.2 Recruitment using an external recruitment agency: Trialfacts ........ 22
    4.1.3 Online Screening Questionnaire ........ 22
  4.2 Study assessments and clinic visits ........ 22
  4.3 Outcome Measures ........ 26
    1.1.1 Data Collection methods ........ 27
  4.4 Potential Benefits ........ 30
5. Assessment of Safety ........ 30
  5.1 Adverse Events ........ 30
  5.2 Serious Adverse Events ........ 30
  5.3 Reporting ........ 31
    5.3.1 Advisory Committee ........ 31
6. Statistics ........ 32
  6.1 Original statistical analysis plan and Sample size calculations ........ 32
7. Ethics ........ 33

7.1 Clinical Trials Registration ........................................ 33
7.2 Informed Consent ........................................ 33
7.3 Confidentiality ........................................ 33
7.4 Storage of data ........................................ 34
7.5 Storage of biological samples ........................................ 34
8. Data and Record Keeping ........................................ 34
8.1 Study documentation ........................................ 34
8.2 Data management ........................................ 35
8.3 Authorship ........................................ 35
8.4 Competing interests ........................................ 35

# 1. Background/Significance

For shift workers there is a 23% increased risk of developing obesity and 44% increased risk of developing type 2 diabetes (1, 2). These risks remain even after controlling for lifestyle and socioeconomic status (3, 4). There are currently 1.5 million Australians (~16% of workforce population) who are at risk (5). The risk of obesity and type 2 diabetes in this population group do not appear to be related solely to overall energy intake (6). Previous research has shown that meal timing i.e. eating late into the night, as is common among shift worker, is associated with metabolic complications such as impaired glucose tolerance and insulin resistance, potentially contributing to weight gain (7, 8). However, there is currently a lack of evidence to identify effective weight loss strategies in shift workers with obesity.

Intermittent fasting (IF) is an umbrella term for dietary strategies that considers both meal timing and energy restriction. For regimens that allow consumption (typically 20-25% of usual daily energy intake) the term modified fasting is used. An example of modified fasting is twice-per-week-fasting. This diet involves alternating between ad libitum 'feeding' days (5 per week) and 'fasting' days (2 per week). This particular approach to weight loss, more commonly known as 5:2, has been shown in the general population to improve dietary compliance (9) and is associated with consistent improvements in insulin resistance (quantified by Homeostatic Model Assessment for Insulin Resistance (HOMA-IR)) (10).

Despite the success of modified fasting, its efficacy in populations that have a high incidence of obesity and unusual working schedules has not been tested. The flexibility in choosing 'fasting' days should enable shift workers to tailor their dietary intakes around shift schedules. Furthermore, designating 'fasting' days to correlate with night shift work, to avoid night time eating, may further improve metabolic health outcomes over other weight loss strategies. We will use a three-arm parallel intervention study to examine three dietary strategies on weight loss in night shift workers: 20% continuous energy restriction (CER), 5:2D (day fast), and 5:2N (night fast).

**Please note that in our published protocol (https://pubmed.ncbi.nlm.nih.gov/35473743/) we used the terms 5:2D and 5:2N. From herein the terms IF:2D and IF:2N will be used instead of 5:2D and 5:2N respectively.**

# 2. Aims and Hypothesis

## 2.1 Aims

The aim is to identify a weight loss strategy for night shift workers that is feasible and flexible to their needs The effectiveness of three weight loss approaches will be examined: continuous energy restriction (CER); a 5:2 protocol with fasting during the day (IF:2D); and during the night shift (IF:2N). The primary outcome measure is weight loss. The study is also powered (co-primary outcome) to detect a change in insulin resistance (HOMA-IR). Feasibility will be assessed using a mixed-methods approach combining retention with post-intervention interviews and longitudinal audio diaries to understand the enablers and barriers to compliance.

## 2.2 Hypotheses

In our sample of shift workers, we hypothesise that:

1. Diet interventions will all result in significant weight loss across time (IF:2D=IF:2N=ER)

2. Insulin resistance (HOMA-IR) will be lower in the IF:2D compared to CER, and the IF:2N compared to the IF:2D (IF:2N<IF:2D<ER)

3. Study retention will be higher in the IF:2D and IF:2N compared to the CER (IF:2D,IF:2N>ER)

# 3. Trial Design

A multi-site 18-month 3-arm parallel randomised trial (24-week intervention with 12-month follow-up). The study will be conducted by Monash University in collaboration with the University of South Australia (UniSA).

This study will be undertaken with overweight/obese shift workers in a free-living environment. Participants will be recruited from the general community either by word-of-mouth, promotion via media announcements, social media (i.e., Twitter, Facebook, Instagram), flyers and through contact with organisations that employ shift workers, including but not limited to those in the industries of; healthcare and/or clinical facilities, emergency services, manufacturing, facility maintenance and security, and transport. Participants will not be recruited whilst attending a healthcare and/or clinical facility as a patient or client.

## 3.1 Treatment

The three interventions are all targeted weight loss strategies aiming to reduce energy intake by approximately 20% of energy requirements overall, with the behaviour change component centred around individualised dietary counselling by the study dietitians located at both sites. No recommendations are provided on changing physical activity. Over the course of the 24-week weight-loss intervention participants will meet individually with a study dietitian on nine occasions (baseline, 2, 4, 6, 8, 12, 16, 20 and 24 weeks). Each visit is approximately 30 minutes except for the baseline visit where 60 minutes is allocated. Study foods for the fasting days (IF:2D and IF:2N) are provided in the weight loss phase of the study. Foods for the fasting days typically consist of one main meal and two small snacks. Participants randomised to the CER intervention are also provided with the same foods, and will work with the study dietitians to incorporate the foods into their diet plan, to ensure that the support across groups is consistent. Participants randomised to the 5:2 groups are asked to restrict their energy intake to 2100-2500kJ for two days a week in conjunction with five *ad libitum* eating days. For the IF:2N group the two fasting occasions are allocated on days that include a night shift. For the IF:2D group the two fasting occasions are allocated on days that included a day shift or a rostered day off. On non-fasting days, participants are asked not to overcompensate for fasting days, or over-restrict their energy intake to mimic fasting days. The CER participants will work with the study dietitians to achieve a daily 20% energy restriction based on the information provided in the 7-day food diary collected prior to baseline, they will be provided with a brochure of the Australian Guide to Healthy Eating (Appendix 1). All study participants will receive a printed list of low-calorie vegetable-based foods and condiments/calorie free fluids they are permitted to consume ad libitum (Appendix 2).

**COVID update to protocol**

In March 2021, all visits were moved to tele-health because of the pandemic and no new participants were recruited for a number of months (this varied across sites). Once the study was deemed essential, recruitment recommenced, and visits returned to face to face but in a much-reduced capacity. The following changes have been formalised as part of an updated protocol. The consults at 2, 4, 6 and 8 weeks were moved to tele-health and remain online (see consent form version 4 and explanatory statement version 9). The 12-week visit remains face to face. Visits at 16 and 20 weeks also moved to tele-health. As such six face to face visits were moved to telehealth as

part of the active weight loss period in total. As recent research has suggested that infection with Covid-19 may affect some of the blood markers measured in the study, participants will be asked about Covid-19 infection history when they attend the research facility for study measurements (Appendix 34_Covid-19 questionnaire). An email will be sent to participants who have already completed the final visit of the study asking them about their Covid-19 infection history (Appendix 35_Covid-19 questionnaire email).

#### *3.1.1 Diet 1: Day fasting diet (IF:2D)*
For five days of the week participants will eat their usual diet, and on two days of the week (the two 'fast' days) they will be required to restrict their energy intake to 2100 kJ/day for females and 2500 kJ/day for males during the day shift. Participants will be provided with study food on each of the energy restriction days. For this study day shift is considered the 24-hour period is counted from 12am (midnight) to 12am the next day.

#### *3.1.2 Diet 2: Night fasting diet (IF:2N)*
For five days of the week participants will eat their usual diet, and on two days of the week (the two 'fast' days) they will be required to restrict their energy intake to approximately 2100 kJ/day for females and 2500 kJ/day for males during the night shift. Participants will be provided with study food on each of the energy restriction days. For this study night shift is considered the 24-hour period from 12pm (noon) to 12pm of the next day.

#### *3.1.3 Diet 3: 20% Continuous daily Energy Restriction (20% CER)*
Based on a participant's age, height and weight, the Mifflin ST Jeor equation (11) is used to calculate basal metabolic rate (BMR). Taking into consideration a participant's physical activity levels (PAL), these calculated energy requirements will be reduced by 20%. Strategies devised for participants will be based on habitual diet (recorded in the run in to Baseline period). Participants will be provided with study meals on two days of the week so that the same level of support is provided to each of the three intervention groups.

### 3.2 Randomisation
Following completion of baseline measurements, participants will be randomised to one of three dietary strategies (coded as 0, 1 or 2) by an independent body, the National Medical Health and Research Council Clinical Trials Centre (University of Sydney, Australia) interactive voice response system, to maintain allocation concealment. Treatment allocation is based on minimisation, balancing study site, age and sex between treatment groups. The research dietitians providing the dietary consultation cannot be blinded to the randomisation process as they provide the appropriate intervention guidance. Participants, once randomised, are no longer blinded to the intervention, but will be asked not to disclose their dietary allocation to other work colleagues participating in the trial. Analyses of primary outcomes will be conducted by a statistician who is not involved with the randomisation process and as such is blinded to the dietary intervention allocation

### 3.3 Blinding
As the participants are consuming diets which are easily identified, the participants cannot be blinded. Researchers analysing data will remain blinded until the completion of statistical analysis. Decoding procedures will not be necessary during the study because the participants will know which foods they are consuming.

### 3.4 Definition of a Completed Subject
A subject will be considered completed when they have completed the 12-month maintenance phase.

### 3.5 Source Data
Measures of height, weight, body mass index (BMI), waist circumference (WC), and blood pressure (BP) will be recorded directly into the Case Report Form (CRF) on REDCap and this will be considered

the source data. Hardcopy the Case Report Forms (CRF) will be stored in a secure cabinet on each site. Electronic copies of results of blood and faecal analysis will be stored on labArchives. Questionnaires will be completed directly in REDCap and will be source data. The source data collected from DXA scans will be stored as electronic file on the DXA machine and on a secure electronic server and also in hard copy in a secure cabinet on each site. Hardcopy food diaries will be stored in a secure cabinet on each site. Dietary information will be stored with the CRF in labArchives. Sleeping patterns will be collected by an activity monitor GENEActiv Original and recorded using hardcopy sleep diaries. Sleep-diary data will be stored in the CRF in labArchives, Geneactiv data will be exported from the watch in to excel which will become the source document and stored in labArchive.

## 3.6 Selection and Withdrawal of Subjects

Only participants who fulfil all the inclusion criteria and are not excluded by any exclusion criteria will proceed to the Intervention Period of the study.

### *3.6.1 Inclusion Criteria*

Participants are eligible to be included in the study only if they meet all the following criteria:

- Current night or rotating shift worker
- Currently having 2-night shifts per week. A night shift is counted as 6 hours between 10pm and 6am, inclusive of travel time.
- Aged ≥ 25 and ≤ 65 years
- BMI ≥ 28 kg/m$^2$ for non-asian, BMI ≥ 26 kg/m$^2$ for Asian participants
- Weight stable in the past 3 months with weight change of no more than 5kg
- Have no genetic conditions (e.g., Prada Willi or Down’s syndrome)
- Not diagnosed with a medical condition (i.e. Type 1/Type 2 Diabetes, cardiovascular disease (stroke, heart failure, heart attack) inflammatory bowel disease (e.g. ulcerative colitis and Chrohns)
- Do not require drug-therapy (e.g., Insulin, Levothyroxine, anti-depressants, statins)
- Do not require drug therapy for diabetes.
- Any condition that will affect your participation in the study
- Have not had previous weight loss surgery
- Not pregnant, planning a pregnancy or breastfeeding, and prepared to do a pregnancy test
- Able to complete a 6-month weight loss intervention and a 12-month maintenance follow-up
- Have no dietary allergies or dietary restrictions that prevent consumption of provided study-meals
- Not taking extended leave from work in the next 6 months

### *3.6.2 Exclusion Criteria*

Participants will be excluded from the study if they meet any of the following criteria:

- BMI kg/m$^2$ for Caucasian less than 28, and for Asian, less than 26*
- Day time workers or those who work what is perceived to be normal working hours (i.e., those who do not work between 1am and 6am)
- Working <4 night shifts worked per fortnight

- Aged less than 25 years and aged greater than 65 years
- Those who consume 4 or more standard drinks on one occasion, at a "daily or almost daily" occurrence, as they are at risk of alcohol related harm more frequently.
- Diagnosed with diabetes or cardiovascular disease
- On drug therapy for diabetes
- Taking medications known to alter body composition or metabolism e.g. thyroxin, insulin sensitisers, glucocorticoids, anti-hypertensive, statins, or anti-depressants. This will be assessed on a case by case basis. We will only exclude participants based on this criterion if they express that the medication has affected their weight or body composition and if they have indicated that is has not been dose stable in the past 3-6 months.
- Obesity due to secondary causes / genetic disorders (e.g. Down's Syndrome, Prader Willi) or endocrinology related disorders (e.g. hypothyroidism or growth hormone deficiency)
- Women who are pregnant, planning a pregnancy or breastfeeding.
- Women with diagnosed Polycystic Ovarian Syndrome
- Anyone with diagnosed gastrointestinal disease e.g. Crohn's Disease
- Previous weight loss surgery
- Fitted with an implanted cardiac defibrillator (i.e. pacemaker) or artificial limb
- Unable to complete a 6-month weight loss intervention (e.g., severe dietary allergies/intolerance)

<u>The following exclusions are to reduce confounding factors:</u>

- Waist circumference is not to be used in isolation as an exclusion criterion but should be taken into consideration if a borderline BMI is presented. <94 cm (Non-Asian men), <90 cm (Asian men) < 80 cm (women). Participants with borderline BMI (27.5 – 28.0 kg/m$^2$ for non-Asian and 25.5 – 26 kg/m$^2$ for Asian participants) and low waist circumference will be excluded. Borderline BMI and waist circumference above pre-determined cutoffs (as above) can be discussed as a team and considered eligible.

<u>The following exclusions are to reduce attrition rates:</u>

- Those who are expecting to change shift schedule in the next 6 months following commencement of study
- Those who are expecting to take extended leave from work in the next 6 months following commencement of study (> 2 weeks).

#### *3.6.3 Withdrawal Criteria*

Every reasonable effort will be made to retain study participants.

The following may constitute reasons for withdrawal of a participant from the study:

- The need to take a medication or treatment, which in the opinion of the investigator, may interfere with study measurements
- Withdrawal of consent by the participant

If a participant withdraws from the study, they will not be replaced. If a participant is removed from the study or declines further participation prior to completion, an early withdrawal visit will be performed if possible. An early withdrawal visit will be conducted if the participant has completed the weight loss phase plus one month of maintenance.

The participants are informed that if they withdraw, personal and health information, and measurements collected during their participation, may still be used. Participants can choose to opt-out of this. The participant will be asked to sign and date a withdrawal form, if they are unable or unwilling to do this the form may be filled out on their behalf by a senior researcher. Bluetooth scales will need to be returned if participants withdraw from the study prior to completing the weight loss phase. The date of withdrawal will be logged in the participants record in REDCap, under the "withdrawal/lost to follow up" button.

# 4. Study Assessments and Procedures

## 4.1 Recruitment

Participants will be recruited using the following techniques:

- Direct contact with shift working organisations. We will send them a brief outline of the study, including research team's contact details, rationale of the study, what it requires from the participants and possible benefits for the workplace and the participants. Upon review, we will ask them to consider advertising this study in their workplace by distributing flyers and information sheets to their employees and displaying them around the workplace. The flyer and information sheet will contain the research team's contact details and an URL to the Online Screening Questionnaire. Investigators may also visit workplaces and speak to the employees in order to explain the study details and answer any questions. This will only be conducted if the responsible personnel of the workplace grant investigators permission to do so.
- Placing advertisements on www.gumtree.com.au and on various social media platforms including Facebook, Twitter and either the Monash University or UniSA websites
- Placing advertisements in newspapers such as The Leader (local newspaper)
- Radio advertisements
- Additional details and information will be placed on a Monash owned website. This will include our screening questionnaire and participant explanatory statement
- Participants from previous studies have ticked a box on the consent form indicating their willingness to be contacted for future studies and/or to have their name included on a database of participants held at Be Active Sleep Eat (BASE) Facility (ethics approvals: CF16/340 - 2016000156), and other research studies in shift workers (2018-10898-19341 and 2018-8619-22517)

### *4.1.1 Video recruitment using current participants*

Participants who are currently engaged in the active weight loss phase of SWIFt will be consented to be filmed describing their experiences of the study. This will be a one-off strategy as once the videos are filmed; we will be able to use them multiple times as part of our recruitment campaigns. An addendum to consent will be used to gain permission from the participants (Appendix 32). Participants will either

i) Self-record a video using a mobile device and/or

ii) Be filmed in person by a company specialising in the development of short videos for distribution. (approved Monash supplier). The videos may be filmed at home and/or in the workplace

Information on how to record video footage, a list of proposed topics to discuss (Appendix 8) and a consent form (addendum to their current consent Appendix 32) will be provided to participants who are interested in being filmed. The sub-contractor (film company) will edit the footage so that it is suitable for use on our study website, social media campaigns and can be distributed to shift working companies etc for display on their own websites.

#### *4.1.2 Recruitment using an external recruitment agency: Trialfacts*

The use of recruitment companies to help identify eligible participants will help with the recruitment process. Companies, such as Trialfacts, offer an alternate strategy to recruitment that benefit from years of experience setting up and administering recruitment campaigns. They tend to use social media strategies including Facebook and Instagram. The research team provide ethically approved documents such as screening questionnaires, consent forms, eligibility criteria and participant information to enable Trialfacts to develop a recruitment campaign. Trailfacts remit is to provide a designated number of eligible participants as agreed with the research team. The transition from eligible to enrolment is the role of the research team, not the recruitment company.

1. Trialfacts advertise the study
2. Potential applicants are screened for eligibility by Trialfacts using the SWIFt screening questionnaire
3. Applicants deemed to be eligible book a time to call with the SWIFt research team
4. If eligibility is confirmed the SWIFT team move forward with enrolment in the study

Trialfacts have their own privacy policy and data security policy.

#### *4.1.3 Online Screening Questionnaire*

The Online Screening Questionnaire can be accessed from the SWIFt website or via scanning a QR code or entering a URL. The questionnaire data will be housed on REDCap, a secure online platform, supported by Monash University. Participant contact details are included in this questionnaire, so that we can inform them of their eligibility on conclusion of the questionnaire.

Interested participants will progress to the online questionnaire via two avenues.

> Interested participants will contact the study team for further information. The study team will then provide participants with detailed information about the study and send consent form/s via postal mail or email. Participants will be directed to the online screening questionnaire to assess eligibility. In the event that interested participants cannot access the online screening questionnaire, this may be conducted over the phone or as a hard copy.
>
> **OR** Interested participants will visit the URL of the online screening questionnaire. The explanatory statement and consent form are embedded in the online screening questionnaire. Potential participants must read the explanatory statement and sign the consent form before commencing the questionnaire.

The research team will review responses and if deemed eligible, participants will be contacted and scheduled to attend a face to face meeting with a researcher. Non-eligible participants will be advised either by phone or electronically.

### 4.2 Study assessments and clinic visits

In total the SWIFt study involves 13 consultations with the researcher. Seven of these visits are face to face, the remainder are over the phone or via video-conference (e.g. Zoom).

***Screening for eligibility and questionnaire completion (~60 minutes)***

This face to face screening appointment will take place at the BASE facility in Melbourne or UniSA Clinical Trial Facility in Adelaide.

During this visit participants will:

- Have the project explained by one of the study investigators, have the opportunity to ask questions and then sign a consent form
- Confirm medical history and any current medications.
- Have height and weight measured to confirm eligibility
- Complete a series of online questionnaires relating to work schedule, sleep habits, mental well-being, quality of life, and physical activity
- Be given a seven-day weighed food diary to be completed prior to the next visit. The diary does not need to be consecutive but needs to include at least 2 night shifts.
- If in Melbourne, be given a stool collection kit to collect one sample for gut-bacteria analysis prior to the next visit.
- Be lent an activity monitor called GENEActiv (similar to a fitbit) to wear on the *non-dominant* wrist to measure sleeping and physical activity patterns for 2 weeks.
- Be asked to complete a daily sleep diary and work diary for 14 consecutive days prior to the next visit (sleep and work diaries to be collected at the same time as GENEActiv watch is worn).
- At screening (or any time prior to the end of the Baseline visit), be asked their meal preferences e.g., pasta, poultry or meat based, fish and/or vegetarian. After the baseline session a follow up email will be sent with the participants next booking details, reminders for diet/sleep/work diaries and GeneActiv watch and an invitation to get in touch with the researcher directly if they have any queries or difficulties.

***Weight loss phase***

The participants will be randomised to one of the three dietary strategies. This randomisation will be undertaken by an organisation separate to the university to ensure the researchers are not involved in this process. This process is important, as it reduces the risk of research bias.

***Baseline visit (~1-2 hours)***

Baseline visit is at a minimum 2 weeks after the screening visit (first visit). The participants will be fasted for 10 hours prior to this appointment. This visit should **NOT** be straight after the participant finishes night shift.

At this visit researchers will collect the GENEActiv watch, stool sample (Melbourne only) and the completed food, sleep and work diaries.

During this visit the participant will undergo a number of tests.

Details of the procedures are outlined below:

- Measure blood pressure, height, weight, waist circumference, body composition and bone mineral density measured (using a DXA scanner)*. For females only, a pregnancy test for dual x-ray absorptiometry (DXA) will be offered.
- Collect a fasting blood sample (approximately 40 mL)
- Measure AGEs (advanced glycation end-products) using The AGE Reader (Melbourne only)
- Be given Bluetooth scales to weigh themselves weekly to fortnightly at home. On completion of the study, participants will be able to keep the scales valued $100 as long as they have completed the weight loss phase up to 6-months.

- Undertake a short test of functional mobility called the ‘timed up and go’
- Be provided with meals to consume over the next 4- 6 weeks as part of the weight loss intervention. Approximately six weeks of meals are supplied, and participants are scheduled to collect an additional six weeks’ worth of food after six weeks on intervention.
- Have a consultation with a dietitian who will inform the participant which dietary weight loss strategy they have been randomised to and guide the participant on how to comply with the diet
- Optional: by invitation from a member of the SWIFt research team, take part in semi-structured interviews and/or keep a longitudinal audio diary detailing their experience on the SWIFt study. Semi-structured interviews with SWIFt participants will be recorded in person, via face to face video-conference (e.g. Zoom) or a telephone call. Interviews will take approximately 45 minutes. The longitudinal audio diaries (LADs) will be collected fortnightly during the 6-month weight loss intervention. Each recording will be approximately 5 minutes in length. The interviews and audio diaries with SWIFt study participants are aimed at eliciting participant experiences during the study. Participants can request a copy of the transcript and upon request make edits to responses. All responses from participants will be confidential. Data from the interviews and LADs will be saved on secure Monash supported servers (REDCap/Lab Archives).

***** the DXA scanner is diagnostic of osteoporosis. If the results meet the criteria for osteoporosis and were unaware prior to participation in the study, participants will be given a copy of the scans, including the diagnostic (t-score) data and recommended to approach their general practitioner (GP) with their results for further testing. This may impact inclusion in the study. (Diagnostic capability is Melbourne only). All (Melbourne) participants on completion of the study (18 months after the baseline session) will be sent a copy of their DXA report for their reference.

***Dietetic consults – Weight loss phase***

During the weight loss phase there will be up to eight video-conference (e.g. Zoom) appointments. These will be held <u>every 2 weeks</u> for the first 8 weeks (4 sessions), and then <u>every 4 weeks</u> (4 sessions, weeks 12, 16, 20 and 24).

These face to face video-conference (e.g. Zoom) appointments are for diet review by a dietitian, weight measurement and collecting food checklists, where required. In Adelaide, researchers will also be collecting 24-hour food recalls at these sessions.

***At 12 weeks during weight loss -*** participants will be required to visit the facility. The participants will be fasted for 10 hours prior to this appointment. This visit should **NOT** be straight after the participant finishes night shift. At this session researchers will measure blood pressure and collect a fasting blood sample. Participants will also have their body composition measured, a 24-hour dietary recall taken and have a dietetic consult face to face.

***End of weight-loss phase (24 weeks) -*** the <u>final visit of the weight loss phase</u> will take approximately 60 minutes. Please note, participants will be required to visit the facility 2 weeks prior to pick up the activity monitor, diaries and stool collection kit (Melbourne) or will be sent a pack containing these items (Adelaide).

- During this visit participants will be required to repeat the assessments performed at screening and baseline visits. In summary, these include:
- Measures of blood pressure, height, weight and waist circumference

- DXA scan
- Fasting blood sample
- 1 x stool sample (Melbourne only)
- AGE reading (Melbourne only)
- GENEActiv activity monitor – 2 weeks of data
- Sleep and work diary – 2 weeks (concurrent to GENEActiv activity monitor being worn)
- Dietary diary – 7 days
- Questionnaires (link sent 3 days prior to the appointment via email)
- By invitation – semi-structured in-depth interview – recorded in person via video-conference (e.g. Zoom) or a telephone call

Via a face to face consult (in person or via video-conference (e.g. Zoom)) the participant will then be given individualised dietary instruction, dependent on the intervention they are following, for the maintenance phase of the project, which will be followed for 12 months. This will be very similar to the weight loss phase except that the energy intake will be reassessed if weight loss has exceeded 10 kg.

If the participant is participating in the semi-structured interviews either in person, via video-conference (e.g. Zoom), or via telephone call this will be conducted over the 18 months (before they start, at the end of weight loss phase, and during the maintenance phase).

**Participation Outline – 12-month maintenance phase**

The maintenance phase is for 12 months.

***One-month phone call***

Participants will receive a surprise (not pre-booked) *telephone call (approximately 15-20 minutes) in one month* to review progress. Researchers will attempt to call three times and log each attempt in REDCap. During the call a date and time will be scheduled for a face to face video-conference (e.g. Zoom) 2 months into the maintenance phase (one month after the phone call).

***Dietetic consult - 2 months and 6 months (maintenance)***

These two sessions will be held at 2 months and 6 months post the start of the maintenance phase via face to face video-conference (e.g. Zoom) and will take 30 minutes. No tests or samples will be collected at these visits.

***Data collection - Final visit - end of maintenance***

After 12 months of maintenance (18 months post baseline and 12 months post maintenance phase) participants will be required to attend the facility for the **final visit** (~60 minutes) to repeat the assessments performed at the end of the weight loss phase (as outlined in section 4.2). Prior to this visit participants will record dietary intake for seven days, wear an activity monitor (GENEActiv) on their non-dominant wrist to measure sleeping patterns, provide a stool sample, and to complete a daily sleep and work diary for 2 weeks (concurrent to GENEActiv activity monitor being worn). Please note: participants will be required to visit the facility 2 weeks prior to pick up the activity monitor, diaries and stool collection kit (Melbourne) or will be sent a pack containing these items (Adelaide).

**Participants: Semi-structured interviews (visits 2, 10 and between 12 and 13) and longitudinal audio diaries (LAD) (every 2 weeks for the 24-week weight loss phase).**

Participants can opt in to be interviewed and/or keep a longitudinal audio diary detailing their experience on the SWIFt study. Semi-structured interviews with SWIFt participants will be recorded

in person, via face to face video-conference (e.g. Zoom) or a telephone call. Interviews will take approximately 45 minutes. The longitudinal audio diaries (LADs) will be collected fortnightly during the 6-month weight loss intervention. Each recording will be approximately 5 minutes in length. The interviews and audio diaries with SWIFt study participants are aimed at eliciting participant experiences during the study. Participants can request a copy of the transcript and upon request make edits to responses. All responses from participants will be confidential. Data from the interviews and LADs will be saved on secure Monash supported servers (REDCap/Lab Archives).

**Researchers: Semi-structured interviews with researchers**

Semi-structured interviews with study researchers involved with the dietetic consults will be recorded in person, via face to face video-conference (e.g. Zoom) or a telephone call. Interviews will take approximately 45 minutes. Interviews with SWIFt study researchers are aimed at exploring the perceptions of the factors that mediate delivery of the intervention as per the study protocol, the effective features Participants are able to request a copy of the transcript and upon request make edits to responses. All responses from participants will remain confidential. These interviews will take place after a number of participants have completed the intervention. A separate consent form and explanatory statement have been developed for the researchers (Appendix 29 and 30).

**Participation Outline – 24 follow-up phase (OPTIONAL)**

At 24-months post completion of the SWIFt intervention we will repeat some measures made at 18 months. This will help us to understand the long-term success of the three dietary interventions that form part of the SWIFt project. This part of the project is a new addition to the SWIFt project and Optional. We will ask participants to answer a short questionnaire that includes information on their current employment status and weight.

### 4.3 Outcome Measures

Assessments include the following:

| | | **Assessment timepoint** | | |
|---|---|---|---|---|
| **Measure** | **Instrument** | **Baseline (week 0)** | **Week 24** | **Month 18** |
| Primary outcomes | | - | - | - |
| Weight (kg) | Measured weight (kg) | ✓ | ✓ | ✓ |
| HOMA-IR | (Glucose × insulin)/22.5 | ✓ | ✓ | ✓ |
| Secondary outcomes | | | | |
| Body mass index | Measured weight (kg) and height (m) | ✓ | ✓ | ✓ |
| Body composition | DXA and/or BIA* | ✓ | ✓ | ✓ |
| Waist circumference | Measured waist (cm) | ✓ | ✓ | ✓ |
| Physical activity | International Physical Activity Questionnaire - Long Form | ✓ | ✓ | ✓ |
| Activity and sleep monitoring | GENEActiv accelerometer, sleep and work diaries | ✓ | ✓ | ✓ |
| Blood pressure | Sphygmomanometer (mm Hg) | ✓ | ✓ | ✓ |
| Physical functioning | Timed up and go | ✓ | ✓ | ✓ |
| Fasting insulin | ELISA (mU/mL) | ✓ | ✓ | ✓ |
| Fasting glucose | Indiko (mmol/L) | ✓ | ✓ | ✓ |
| Fasting TAG | Indiko (mmol/L) | ✓ | ✓ | ✓ |
| Fasting cholesterol (total and subsets) | Indiko (mmol/L) | ✓ | ✓ | ✓ |
| HbA1C | Indiko (mmol/mol) | ✓ | ✓ | ✓ |
| Gut microbiome* | 16SrRNA sequencing | ✓ | ✓ | ✓ |
| Tissue AGEs* | Skin autofluorescence | ✓ | ✓ | ✓ |
| Bone mineral density* | DXA | ✓ | ✓ | ✓ |

| | | | | |
|---|---|---|---|---|
| Quality of life | AQoL-8D | ✓ | ✓ | ✓ |
| Emotional state | Depression, Anxiety and Stress Scale | ✓ | ✓ | ✓ |
| Habitual dietary intake | 7-day food diary | ✓ | ✓ | ✓ |
| Behaviours within shift system | Survey of shiftworkers | ✓ | ✓ | ✓ |
| Osteoarthritis symptoms | Womac osteoarthritis index | ✓ | ✓ | ✓ |
| Compliance and retention | Time to dropout | | | |
| Motivations for losing weight and the barriers and enablers to weight loss | Interview | | | ✓ |
| Weight and employment status at 24 months follow-up | 24-month follow-up questionnaire | | | |
| Exploratory analyses (effect modifiers/confounder) | | | | |
| Chronotype | Composite Scale of Morningness | ✓ | | |
| Genotype | Polymorphisms in clock genes | ✓ | | |
| Sleep quality | Basic Nordic Sleep Questionnaire | ✓ | ✓ | ✓ |
| Sleep timing | Survey of Shift Workers | ✓ | ✓ | ✓ |
| Shift work disorder | Shift Work Disorder Questionnaire | ✓ | ✓ | ✓ |
| Provided foods | Food checklists | | ✓ | ✓ |
| Demographics | Age, sex | ✓ | | |
| Socioeconomic factors | Occupation and working hours | ✓ | ✓ | ✓ |
| Covid-19 infection history | Questionnaire | | | |

*Monash site only.

### *1.1.1 Data Collection methods*

***Anthropometric measures***

Height will be measured to the nearest 1 mm with a stadiometer at baseline. BMI will be calculated as weight/height squared (kg/m2). Body weight (primary outcome) will be measured two times at each visit (with the average value calculated) using calibrated electronic scales (Melbourne; SECA 515, Ecomed, Adelaide; SECA 703). Waist circumference, body composition and bone density will be measured at three timepoints: baseline (week 0), end of weight loss (24 weeks) and end of weight maintenance phase (18 months). Waist circumference will be measured to the nearest mm, according to the protocol of the International Society for Anthropometry, using a thin non-flexible metal measuring tape at the narrowest point of the abdomen or, if there is no obvious narrowing, at the midpoint between the lower costal (10th rib) border and the iliac crest (37). Body composition will be determined using dual-energy X-ray absorptiometry (Lunar [Melbourne ; iDXA Model, Adelaide; Prodigy Model], GE Healthcare, Madison, Wisconsin, USA). Total body fat mass (%, kg), total body fat free mass (%, kg) and truncal fat and fat free mass (kg) will be recorded. At the Melborune site only, body composition is also measured using bioelectrical impedance analysis (SECA, 515, Ecomed). Bone mineral density (spine and hip) is also being measured (Melbourne only).

***Biochemical measures***

Blood parameters

Participants will have a fasting venous blood sample collected at baseline, 12 weeks, 24 weeks and 18 months.
Plasma collected will be used to assess glucose and insulin (to calculate primary outcome HOMA-IR). Serum collected will be used to assess blood lipids, including TAG, total cholesterol, LDL cholesterol and high-density lipoprotein (HDL) cholesterol. Whole blood will be collected for assessment of HbA1C and possible future assessment of genotype. All samples collected will be centrifuged (except whole blood samples) and stored at −80°C until analysis. Whole blood, HbA1C, serum lipid and

plasma glucose analysis will be conducted on a Thermo Fisher Indiko (Thermo Fisher Scientific, Vantaa, Finland), using commercially available kits as per the manufacturer's instructions. Plasma insulin will be analysed using the Millipore Human Insulin ELISA kit (EZHI-14K, Merck Millipore, Massachusetts, USA) according to the manufacturer's instructions and read on an absorbance plate reader. HOMA-IR will be calculated using the following formula: (fasting plasma insulin (µU/mL)×fasting plasma glucose (mmol/L))÷22.5.

Faecal samples

Participants will collect faecal samples at baseline, 24 weeks and 18 months for gut microbiome analysis using 16SrRNA sequencing. Samples will be frozen in the participants' domestic freezer on collection until being returned to the Monash University BASE Facility at Notting Hill, Melbourne, where they will be stored at −80°C. Analyses will be subject to additional funding.

Skin autofluorescence (SAF)

SAF will be measured (Melbourne site only) in triplicate on the ventral site of each participant's forearm using an Advanced Glycation Endproduct (AGE) Reader (Diagnoptics Technologies, Groningen, The Netherlands). The AGE Reader estimates the level of long-term AGE accumulation in the skin, which are a marker of cumulative glycative and oxidative stress within body tissues. The intraindividual per cent error is <5.0% on a single day and 5.9% for seasonal changes (39). SAF measured by the AGE Reader has been validated against AGE accumulation in skin biopsies, and is recognised as a non-invasive method to predict future risk of T2DM and CVD, independent of traditional risk factors such as glucose, HbA1c and the metabolic syndrome (12).

***Physical measures (measures at baseline, 24 weeks and 18 months)***

Blood pressure

Measurements will be taken using an automated sphygmomanometer (Melbourne Site: SureSigns VS3; Philips, North Ryde, Australia, Adelaide Site: Omron HEM-7320; Omron Health Care, Port Melbourne, Victoria), after participants have been seated at rest for at least 2 min and are in a fasted state. Three consistent measurements (systolic within range of 10 mm Hg and diastolic within range of 5 mm Hg) will be recorded and averaged (13).

Movement Behaviours

Physical activity and sleep will be measured using triaxial accelerometers (GENEActiv; Activinsights, Cambridgeshire, UK), which will be worn on the non-dominant wrist. Participants will be asked to wear the monitor 24 hours/day for 14 consecutive days, only removing it for activities where it may be submerged in water. Activity data will be processed by GENEActiv software (V.3.3), and periods of sleep and non-wear time will be calculated using custom filters (14). Data will be recorded continuously at 30 Hz. The signal vector magnitude of the acceleration, minus gravity, will be computed and summed over 1 min epochs (15). Non-wear will be identified using the method of Choi *et al* (16). A day will be valid if it includes at least 10 hours of waking wear time. Participants with fewer than four valid days will be excluded (17). The algorithm developed by van Hees et al. will be used to analyse and calculate both physical activity and sleep patterns (18).

Timed up and go

The 'timed up and go' has been shown to be a reliable and valid assessment of dynamic balance during functional tasks (19,20). Participants will be timed in seconds as they rise from a seated position, walk 3 m, turn around, walk back and sit back down (20). Three measurements will be recorded and averaged, to provide a measure of functional mobility that correlates to balance and fall risk (21).

***Questionnaires***

Diet diary
A food diary recorded via paper record or the 'Research Food Diary App' (Xyris Software, Australia) will be completed for 7 days (in order to capture work and non-workdays) leading into their baseline, 24 weeks and 18 month visit. The data collected will be used to assess participants' habitual food intake as well as changes in dietary patterns and/or choices (22). Data will be imported into Foodworks V.10, using the Australia Diet and Recipes Analysis (AUSFOODS 2019) database. Average total EI of macronutrients and micronutrients will be determined.

Work diary
Participants will be asked to use a paper diary to self-report the start and finish times of each of their shifts for 14 days leading into their baseline, 24-week and 18-month visit. This will be used to identify participants' usual shift work patterns.

Sleep diary
Participants will be asked to record in a sleep diary the time they went to bed, time they woke, approximately how long they took to fall asleep and any extended periods of time they were awake during the sleep episode. This should take approximately 1 or 2 min per day to complete. The entries from the sleep diaries will be used to confirm sleep data obtained from accelerometer data, or in the case of participants not wearing the accelerometer, the data from the sleep diaries will be used to directly analyse sleeping patterns (23).

***Standardised Questionnaires***

Depression Anxiety Stress Scale-21 (DASS-21)
The DASS-21 is a set of three self-report scales designed to measure the emotional states of depression, anxiety and stress (24), with cut-off scores calculated for conventional severity labels (normal, mild, moderate, severe and extremely severe) for the three emotional states.

Assessment of Quality of Life-8D
A multi attribute utility instrument used to identify the overall well-being of the individual and physical pain subscore. Included items (n=35) relate to happiness, pain, self-worth, coping and relationships (25). Participants are asked to tick the box that best describes their situation as it has been over the past week with options being never, rarely, sometimes, often or always.

International Physical Activity Questionnaires
A 27-question self-report questionnaire will assess physical activity levels. The duration (minutes) and frequency (days) of physical activity in the last 7 days are measured across multiple domains: transportation, recreation (including sport and leisure time), housework, job-related and time spent sitting (26). An overall score is calculated using responses to all questions to classify participants into one of three categories: (1) low/inactive (do not meet criteria for categories 2 or 3); (2) moderate or (3) high.

Survey of Shift Workers
This validated shortened version of the Standard Shift work Index consists of standardised self-report measures of participants' shift details (sequencing, timing, duration, frequency and regularity of shifts) and behavioural patterns, as well as ratings of factors associated with tolerance to shift work patterns (27). While responses to the included questions provide qualitative data, responses to each of the scales are calculated from 1 to 4 or 5.

Basic Nordic Sleep Questionnaire

This standardised 21-item questionnaire uses a combination of five-point scales and open-ended questions which measure the frequency of symptoms related to sleep apnoea and snoring, by quantifying how many nights/days per week the item happened over the past 3 months (28).

Shift Work Disorder Questionnaire

A four-item questionnaire used to assess the risk of shift work disorder, including excessive sleepiness and insomnia (29). Questions, including, 'in the past month, how likely were you to doze off at work during a shift?' are scored on a 4-point scale, ranging from 1 (not a problem/not at all) to 4 (serious problem/highly likely).

Chronotype

Chronotype, an individual's natural inclination regarding the times of day when they prefer to sleep or when they are most alert or energetic, will be assessed by the Composite Scale of Morningness Questionnaire, a 13-item numerical multiple choice questionnaire on a 4–5-point scale (30). These data will be collected once only at baseline.

Osteoarthritis Index

Western Ontario and McMaster Universities Osteoarthritis Index (WOMAC). This 24-item, 3-subscale questionnaire measures pain (5 items), stiffness (2 items) and physical function (17 items) experienced during everyday tasks, that is, walking, using stairs, light domestic duties (31).

### 4.4 Potential Benefits

An individualised dietary consult will be provided at the conclusion of the maintenance phase of the study to participants who would like additional nutritional advice. On request, participants will be provided a final report with summary data and major findings. Participants will be provided with weighing scales to monitor their weight ($100) which they will keep on completion of the study. Participants will be provided with $100 petrol voucher to contribute towards their visits to the clinical facilities. Participants will be provided with 2 meals a week for 24-weeks to improve their compliance to the dietary intervention (equivalent to $240). Participants will receive dietary counselling by a dietitian for weight loss via face to face visits or video-conference (e.g. Zoom), and it is therefore expected that participants will lose weight, which is associated with improvements in health.

## 5. Assessment of Safety

### 5.1 Adverse Events

An adverse event is defined in the International Conference on Harmonization (ICH) Guideline for Good Clinical Practice as "any untoward medical occurrence in a patient or clinical investigation subject administered a pharmaceutical product and that does not necessarily have a causal relationship with this treatment." (ICH E6:1.2).

The participant should be followed until the event is resolved or explained. Frequency of follow-up evaluation is left to the discretion of the investigator.

### 5.2 Serious Adverse Events

A serious adverse event (SAE) is defined as an adverse event that:

- is fatal.
- is life threatening (places the participant at immediate risk of death).

- requires in-patient hospitalization or prolongation of existing hospitalization.
- results in persistent or significant disability/incapacity.
- is a congenital anomaly/birth defect.
- another significant medical hazard.

A hospitalization meeting the regulatory definition for “serious” is any inpatient hospital admission that includes a minimum of an overnight stay in a health care facility. Any adverse event that does not meet one of the definitions of serious (e.g., emergency room visit, outpatient surgery, or requires urgent investigation) may be considered by the investigator to meet the “other significant medical hazard” criterion for classification as a serious adverse event.

## 5.3 Reporting

The investigator is responsible for ensuring that all adverse events observed by the investigator or reported by participants are collected and recorded in the participants’ source documents, in the CRF. Adverse events and serious adverse events will be reported to the Human Research Ethics Committee of Monash Health and Monash University, and UniSA within the appropriate time frame. Adverse events are recorded from the time the participant commences data collection.

The following adverse event attributes must be assigned by the investigator: adverse event diagnosis or syndrome(s) (if known, signs or symptoms if not known); event description (with detail appropriate to the event); dates of onset and resolution; severity; assessment of relatedness to the dietary intervention and action taken. The investigator may be asked to provide follow-up information, discharge summaries, and extracts from medical records. The relationship of the adverse event to the study intervention will be assessed by means of the question: “Is there a reasonable possibility that the event may have been caused by the study intervention?” The investigator should respond to this question with either Yes or No.

Medically significant adverse events considered related to study intervention by the investigator will be followed until resolved or considered stable. It will be left to the investigator’s clinical judgment to determine whether an adverse event is related and of sufficient severity to require the participant’s removal from treatment or from the study. A participant may also voluntarily withdraw from treatment due to what the participant perceives as an intolerable adverse event. If either of these situations arises, the participant should be strongly encouraged to undergo an end-of-study assessment and be under medical supervision until symptoms cease or the condition becomes stable.

The severity grading scale for adverse events is listed below:

Severity assessment definitions:

- Mild – No or mild symptoms that do not interfere with participants daily activities
- Moderate – Symptoms that interference with participants daily activities
- Severe – Symptoms prevent participant from performing daily activities

The investigator should notify the appropriate ethics committee of serious adverse events occurring at the site.

### *5.3.1 Advisory Committee*

This project has an advisory committee comprised of researchers affiliated with the project and external to the project as well as industry representatives, a medical practitioner and consumer representation. The committee meets every 3 months to oversee progress and adherence to the protocol and monitor adverse events. Any major protocol deviations and serious adverse events that are identified during the study will be reported to the committee by the chief investigator (MPB).

Purpose of committee:
Responsible for reviewing milestone attainment. Including (but not limited to):

- Milestones to be in line with budget/funding
- Monitoring recruitment numbers and strategies
- Provide advice pertaining to the progress of the study
- Review the intervention, including the consumer view in regards to implementation

Ethics requirements:

- Reports of any adverse events will be discussed at these meetings
- Ensuring approvals have been obtained across all sites
- Review of data monitoring processes

# 6. Statistics

## 6.1 Original statistical analysis plan and Sample size calculations

Our primary hypothesis is that there will be weight loss in all three groups during the treatment phase. To test this hypothesis, mixed effects models will specify a dependent variable of weight (kg) with a predictor variable of Time (baseline, 24-weeks), controlling for site (Melbourne, Adelaide), and a random effect of subject on the intercept, allowing participants to vary according to individual baseline levels, as well as tracking progress over time, appropriately accounting for serial correlation. We will undertake intention-to-treat and per protocol analyses. The primary effects of interest are the hypothesised change over time in each group. Power calculations are based on previous research, which found a small-medium within-subjects effect for a 14-week workplace weight loss program. Using this as a minimum expected amount of weight loss in a given study group over a six-month time period, (accounting for serial correlation between baseline and 24-weeks) at the end of the treatment phase we would require 93 participants ($\alpha$=0.05, 1-ß=0.8), accounting for an expected 27% drop-out (32), we need to randomise 120 participants into each diet group. Therefore, we need a total of 360 participants.

Descriptive analyses will quantify the effect size of the weight loss in each diet group. We will calculate point estimates and their variability (bootstrapping for 95% Confidence Intervals). These will be calculated for the active phase, and for the maintenance phase (18 months).

A secondary hypothesis is that there will be a differential effect of the diets across time on HOMA-IR (insulin resistance). Based on previous research suggesting the potential metabolic benefits of avoiding food consumption during night time hours, we hypothesise that the 5:2 Night time fasting group will display an improvement in metabolism (as indicated by HOMA-IR) relative to the other diet groups. To test this hypothesis, mixed effects models will specify a dependent variable of HOMA-IR. Models will specify predictor variables of Group (20% ER, 5:2 Day time fasting, and 5:2 Night time fasting), Time (baseline, 24-weeks), and Group*Time, with a random effect of subject on the intercept. The primary effect of interest is the Group*Time interaction effect. Power calculations are based on previous research, which found effect sizes for differences in night and day shift workers in HOMA-IR that ranged from small-medium, to medium-large (f=0.33). Conservatively powering for a small-medium interaction effect, we require 111 participants per group ($\alpha$=0.05, 1-ß=0.8), and accounting for an expected 27% drop-out (32), we need to recruit a total of 423 participants.

Additional analyses will examine changes in HOMA-IR in each of the groups during the maintenance phase (18 months). Mixed effects models will specify a dependent variable of HOMA-IR and predictor variables of Group (20% ER, 5:2 Day time fasting, and 5:2 Night time fasting), Time (24-weeks, 12 months, 18 months), and Group*Time, with a random effect of subject on the intercept.

Additional analyses will also examine compliance rates in the diet groups. Time to drop-out will be measured. A survival analysis (Cox's proportional hazards) model will specify time to drop-out as the dependent, and Group as the independent variable (testing for differences between survival functions).

To make sure that there is sufficient power to adequately test all of study hypotheses, we require the largest sample size estimate from the above calculations. Therefore, to detect all the effects of interest, 141 participants per group (Total = 423) will be recruited over the two sites.

Due to the unanticipated circumstances of follow-up periods in the study coinciding with a global pandemic, and the unknown effect this may have on outcome measures, data on participants' Covid-19 infection history and severity of infection will be collected to include in the statistical analysis as a covariate, if found to have a significant effect on outcome variables.

# 7. Ethics

This study will be conducted according to the guidelines laid down in the Declaration of Helsinki, and all procedures involving human subjects must be approved by the Monash Health Human Research Ethics Committee and the University of South Australia Human Research Ethics Committee (HREC) prior to any research being started.

A copy of the protocol, informed consent form, other written participant information, and any proposed advertising material has been submitted to the HREC for written approval. The investigator will submit and, where necessary, obtain approval from the HREC for all subsequent protocol amendments and changes to the informed consent document. The investigator will notify the HREC of deviations from the protocol or serious adverse events. The investigator will be responsible for obtaining annual HREC approval/renewal throughout the duration of the study.

## 7.1 Clinical Trials Registration

The study is registered on the Australian and New Zealand Clinical Trials Registry. Identifier: ACTRN-12619001035112, https://www.anzctr.org.au

## 7.2 Informed Consent

Consent will be obtained in two parts:

1) Interested participants will be directed to the Online Screening Questionnaire. The potential participant will need to read the Explanatory Statement and sign the Consent Form for the screening questionnaire before being able to attempt the questionnaire. This Consent Form will allow participants to give consent on providing personal information such as age, health status and work schedule, so that their eligibility can be assessed.

Participants will also be asked to provide consent in the same form for the research team to contact and invite them to the BASE research facility for a screening session.

2) When participants arrive at the BASE facility or UniSA Clinical trial Facility for the screening session, they will be given the Explanatory Statement and the Consent Form for the Overall Study. The researcher will explain the study to the participant and answer any queries. The participant will then be asked to sign the Consent Form Overall Study before the screening session begins. If information becomes available that may be relevant to the participant's willingness to continue participating in the trial, the Investigator must inform the participant and a revised informed consent must be obtained.

## 7.3 Confidentiality

Unique participant numbers will be assigned to all participants.

All data and information generated as part of the study will be kept confidential by the Investigator and other site staff and will not be released to any party unless required by law. The Investigator or other site personnel will not use this information and data for any purpose other than conducting the study.

These restrictions do not apply to information which it is necessary for disclose in confidence to HREC solely for the evaluation of the study and information which it is necessary to disclose in order to provide appropriate medical care to a study participant.

De-identified data files collected and created during the project will be stored on ISO 9001 accredited secure Monash University managed storage that requires Monash login to access and automatically logs all activities. All data sets will be preserved in accordance with PROV retention requirements and in compliance with Monash University retention requirements.

A data repository will be selected and used to make the research data sets accessible and reusable. The data repository will publish a persistent digital object identifier (DOI) and re-use license conditions.

### 7.4 Storage of data

Data collected will be stored in accordance with Monash University and UniSA regulations. The collected and coded data will be stored either on a password protected computer file or in a locked filing cabinet on university premises. Information about participants will be stored in a lockable filing cabinet for seven years from date of publication, after which they will be destroyed.

### 7.5 Storage of biological samples

Biological samples will be stored in a -80°C freezer located at the BASE facility, Monash University in Melbourne or City East Campus, University of South Australia. Samples will be non-identifiable. Adelaide samples will be transferred to Monash at the completion of the trial. Samples will be non-identifiable and destroyed at a minimum of 7 years or at the completion of the study and once results have been published They will be destroyed using standard Monash University laboratory practices, and all samples will be labelled with a study participant code only. All participant details with their respective codes will be kept securely by the researcher and separate from actual samples.

The participant consent form gives the option for participants to consent to blood samples being retained for future research projects related to this study.

## 8. Data and Record Keeping

### 8.1 Study documentation

The investigator will maintain a list of appropriately qualified persons to whom he/she has delegated study duties. All persons authorized to make entries and/or corrections on case report forms/database will be included on the Delegation of Authority Form.

The investigator and study staff are responsible for maintaining a comprehensive and centralized filing system of all study-related (essential) documentation, suitable for inspection at any time by representatives from applicable regulatory authorities.

Elements will include:

- Participant files containing completed informed consent forms, and participant identification list
- Study files containing the protocol with all amendments, copies of pre-study documentation and all correspondence to and from the HREC.

In addition, all original source documents supporting entries in the case report forms/ database will be maintained and be readily available.

## 8.2 Data management

Study data will be collected and managed using REDCap electronic data capture tools hosted and managed by Helix (Monash University). REDCap is a secure, web-based software platform designed to support data capture for research studies, providing (i) an intuitive interface for validated data capture; (ii) audit trails for tracking data manipulation and export procedures; (iii) automated export procedures for seamless data downloads to common statistical packages; and (iv) procedures for data integration and interoperability with external sources. All data forms will be deidentified and paper copies will be double entered and stored in the username-protected and password-protected electronic database. Results from this trial will be disseminated through national and international presentations and peer-reviewed journals.

## 8.3 Authorship

Authorship of publications originating from the SWIFt study is to align with authorship guidelines. Authors will follow the approval process outlined in the publication protocol for the development of manuscripts for publication of the SWIFt study.

***Criteria for Authorship***

Criteria for authorship credit set out below is as stated by the National Health and Medical Research Council, Australian Research Council and Universities Australia. While authorship conventions vary across disciplines, below is the minimum threshold for authorship, noting that some journals, disciplines and institutions may require a higher threshold. For authorship on a SWIFt publication, the individual must have made a significant intellectual or scholarly contribution to the particular publication. This means they must have contributed two (the minimum) or more of the following:

- conception and design of the project or output. By project we refer to the body of work being presented in the paper rather than the actual grant per se.
- acquisition of research data where the acquisition has required significant intellectual judgement, planning, design, or input
- contribution of knowledge, where justified
- analysis or interpretation of research data
- drafting significant parts of the research output or critically revising it so as to contribute to its interpretation.

## 8.4 Competing interests

None declared

**References**


1. Sun M, Feng W, Wang F, et al. Meta-analysis on shift work and risks of specific obesity types. Obes Rev. 2018;19(1):28-40.

2. Vetter C, Dashti HS, Lane JM, et al. Night Shift Work, Genetic Risk, and Type 2 Diabetes in the UK Biobank. Diabetes Care. 2018.

3. Banks S. Circadian Misalignment and Metabolic Consequences. In: PR W, editor. Modulation of Sleep by Obesity, Diabetes, Age, and Diet. London: Academic Press; 2014. p. 155-62.

4. Zimberg IZ, Fernandes Junior SA, Crispim CA, et al. Metabolic impact of shift work. Work. 2012;41:4376-83.

5. Australian Bureau of Statistics. Working Time Arrangements, Australia 2009: Canberra, Australia: Australian Bureau of Statistics; 2010 [updated 21 May 2010. Available from: http://www.abs.gov.au/Ausstats/abs@.nsf/0/BBAA98CCB2658F61CA257B5F0021DCB0?OpenDocument.

6. Bonham MP, Bonnell EK, Huggins CE. Energy intake of shift workers compared to fixed day workers: A systematic review and meta-analysis. Chronobiol Int. 2016;33(8):1086-100.

7. Gupta CC, Dorrian J, Grant CL, et al. It's not just what you eat but when: The impact of eating a meal during simulated shift work on driving performance. Chronobiol Int. 2017;34(1):66-77.

8. Leung GKW, Huggins CE, Bonham MP. Effect of meal timing on postprandial glucose responses to a low glycemic index meal: A crossover trial in healthy volunteers. Clin Nutr. 2017.

9. Heilbronn LK, Smith SR, Martin CK, et al. Alternate-day fasting in nonobese subjects: effects on body weight, body composition, and energy metabolism. Am J Clin Nutr. 2005;81(1):69-73.

10. St-Onge MP, Ard J, Baskin ML, et al. Meal Timing and Frequency: Implications for Cardiovascular Disease Prevention: A Scientific Statement From the American Heart Association. Circulation. 2017;135(9):e96-e121.

11. Mifflin MD, St Jeor ST, Hill LA, Scott BJ, *et al*. A new predictive equation for resting energy expenditure in healthy individuals. Am J Clin Nutr 1990;51:241-7.doi:10.1093/ajcn/51.2.241. pmid:http://www.ncbi.nlm.nih.gov/pubmed/2305711

12. van Waateringe RP, Fokkens BT, Slagter SN, et al. Skin autofluorescence predicts incident type 2 diabetes, cardiovascular disease and mortality in the general population. Diabetologia 2019;62:269–80. 10.1007/s00125-018-4769-x

13. Chobanian AV, Bakris GL, Black HR, et al. The seventh report of the joint National Committee on prevention, detection, evaluation, and treatment of high blood pressure: the JNC 7 report. JAMA 2003;289:2560–72. 10.1001/jama.289.19.2560

14. English C, Healy GN, Olds T, et al. Reducing sitting time after stroke: a phase II safety and feasibility randomized controlled trial. Arch Phys Med Rehabil 2016;97:273–80. 10.1016/j.apmr.2015.10.094

15. Troiano RP, Berrigan D, Dodd KW, et al. Physical activity in the United States measured by accelerometer. Med Sci Sports Exerc 2008;40:181–8. 10.1249/mss.0b013e31815a51b3

16. Choi L, Ward SC, Schnelle JF, et al. Assessment of wear/nonwear time classification algorithms for triaxial accelerometer. Med Sci Sports Exerc 2012;44:2009–16. 10.1249/MSS.0b013e318258cb36

17. Migueles JH, Cadenas-Sanchez C, Ekelund U, et al. Accelerometer data collection and processing criteria to assess physical activity and other outcomes: a systematic review and practical considerations. Sports Med 2017;47:1821–45. 10.1007/s40279-017-0716-0

18. van Hees VT, Sabia S, Anderson KN, et al. A novel, open access method to assess sleep duration using a Wrist-Worn Accelerometer. PLoS One 2015;10:e.0142533. 10.1371/journal.pone.0142533

19. Mathias S, Nayak US, Isaacs B. Balance in elderly patients: the "get-up and go" test. Arch Phys Med Rehabil 1986;67:387–9.

20. Podsiadlo D, Richardson S. The timed "Up & Go": a test of basic functional mobility for frail elderly persons. J Am Geriatr Soc 1991;39:142–8. 10.1111/j.1532-5415.1991.tb01616.x

21. Shumway-Cook A, Brauer S, Woollacott M. Predicting the probability for falls in community-dwelling older adults using the Timed Up & Go Test. Phys Ther 2000;80:896–903.

22. Shim J-S, Oh K, Kim HC. Dietary assessment methods in epidemiologic studies. Epidemiol Health 2014;36:e2014009. 10.4178/epih/e2014009

23. Lawrence G, Muza R. Assessing the sleeping habits of patients in a sleep disorder centre: a review of sleep diary accuracy. J Thorac Dis 2018;10:S177–83. 10.21037/jtd.2017.12.127

24. Lovibond SH, Lovibond PF. Manual for the depression anxiety stress scales. Sydney, Australia: Sydney Psychology Foundation, 1995.

25. Richardson J, Iezzi A, Khan MA. Date used in the development of the AQol-8D (PsyQol) quality of life instrument. Monash University Melbourne: Centre for Health Economics, 2009.

26. Booth M. Assessment of physical activity: an international perspective. Res Q Exerc Sport 2000;71:s114–20.

27. Kaliterna L, Prizmić Z. Evaluation of the survey of shiftworkers (SOS) short version of the standard shiftwork index. Int J Ind Ergon 1998;21:259–65. 10.1016/S0169-8141(97)00052-8

28. Shahid A, Wilkinson K, Marcu S. Basic Nordic sleep questionnaire (BNSQ). stop, that and one hundred other sleep scales. New York, NY: Springer, 2011.

29. Barger LK, Ogeil RP, Drake CL, et al. Validation of a questionnaire to screen for shift work disorder. Sleep 2012;35:1693–703. 10.5665/sleep.2246

30. Smith CS, Reilly C, Midkiff K. Evaluation of three circadian rhythm questionnaires with suggestions for an improved measure of morningness. J Appl Psychol. 1989;74(5):728-38

31. McConnell S, Kolopack P, Davis AM. The Western Ontario and McMaster universities osteoarthritis index (WOMAC): a review of its utility and measurement properties. Arthritis Rheum 2001;45:453–61.

32. Morgan PJ, Collins CE, Plotnikoff RC, Cook AT, Berthon B, Mitchell S, Callister R. Efficacy of a workplace-based weight loss program for overweight male shift workers: the Workplace POWER (Preventing Obesity Without Eating like a Rabbit) randomized controlled trial. Prev Med. 2011 May;52(5):317-25. doi: 10.1016/j.ypmed.2011.01.031.

## SWIFt Study protocol

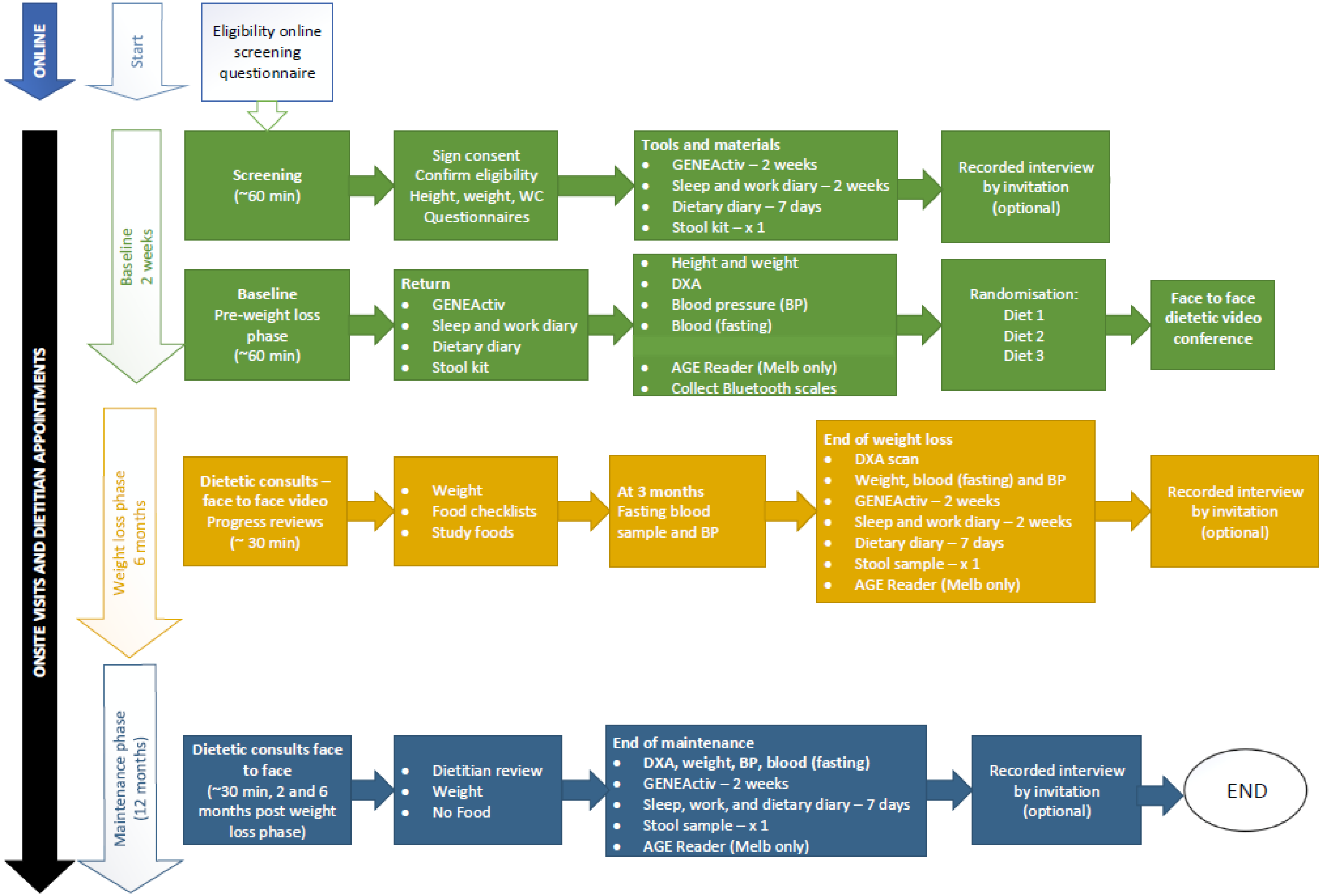

**Appendix 1**

# Healthy eating for adults

## EAT FOR HEALTH AND WELLBEING

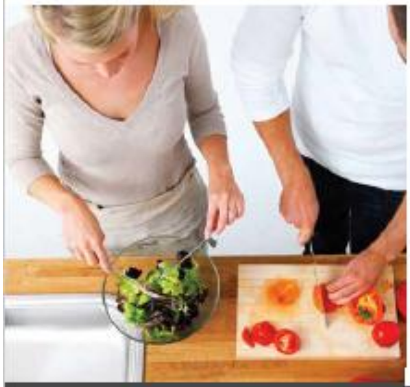

www.eatforhealth.gov.au

## WHAT ARE THE DIETARY GUIDELINES?

The *Australian Dietary Guidelines* provide up-to-date advice about the amount and kinds of foods that we need to eat for health and wellbeing. They are based on scientific evidence and research.

The *Australian Dietary Guidelines* of most relevance to adults are included below:

### GUIDELINE 1:

To achieve and maintain a healthy weight, be physically active and choose amounts of nutritious food and drinks to meet your energy needs.

- Older people should eat nutritious foods and keep physically active to help maintain muscle strength and a healthy weight.

### GUIDELINE 2:

Enjoy a wide variety of nutritious foods from these five food groups every day:

- Plenty of vegetables of different types and colours, and legumes/beans
- Fruit
- Grain (cereal) foods, mostly wholegrain and/or high cereal fibre varieties, such as breads, cereals, rice, pasta, noodles, polenta, couscous, oats, quinoa and barley
- Lean meats and poultry, fish, eggs, tofu, nuts and seeds, and legumes/beans
- Milk, yoghurt, cheese and/or their alternatives, mostly reduced fat

And drink plenty of water.

**Want more information about healthy eating?**

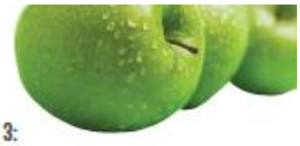

### GUIDELINE 3:

Limit intake of foods containing saturated fat, added salt, added sugars and alcohol.

a. Limit intake of foods high in saturated fat such as many biscuits, cakes, pastries, pies, processed meats, commercial burgers, pizza, fried foods, potato chips, crisps and other savoury snacks.
   - Replace high fat foods which contain predominately saturated fats such as butter, cream, cooking margarine, coconut and palm oil with foods which contain predominately polyunsaturated and monounsaturated fats such as oils, spreads, nut butters/pastes and avocado.

b. Limit intake of foods and drinks containing added salt.
   - Read labels to choose lower sodium options among similar foods.
   - Do not add salt to foods in cooking or at the table.

c. Limit intake of foods and drinks containing added sugars such as confectionery, sugar-sweetened soft drinks and cordials, fruit drinks, vitamin waters, energy and sports drinks.

d. If you choose to drink alcohol, limit intake. For women who are pregnant, planning a pregnancy or breastfeeding, not drinking alcohol is the safest option.

### GUIDELINE 4:

Encourage, support and promote breastfeeding.

### GUIDELINE 5:

Care for your food; prepare and store it safely.

www.eatforhealth.gov.au

## FOODS TO LIMIT: DISCRETIONARY CHOICES

'Discretionary choices' are called that because they are not an essential or necessary part of our dietary patterns. Discretionary foods are high in kilojoules, saturated fat, added sugars, added salt or alcohol. If chosen, they should be eaten only sometimes and in small amounts.

**Examples of discretionary choices include:**

- Sweet biscuits, cakes and desserts
- Processed meats and sausages
- Ice-cream, confectionery and chocolate
- Meat pies and other pastries
- Commercial burgers, hot chips, and fried foods
- Crisps and other fatty and/or salty snacks
- Cream and butter
- Sugar-sweetened cordials, soft drinks, fruit drinks and sports drinks
- Alcoholic drinks

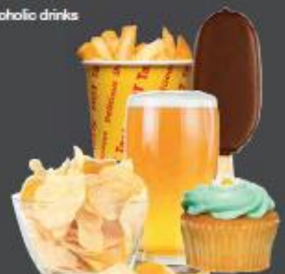

## TIPS FOR CHOOSING NUTRITIOUS FOODS AND DRINKS

**Eating for health and wellbeing is about choosing foods from the Five Food Groups every day, while limiting foods that are not essential to our health.**

- Plan ahead and stock up on basic nutritious foods like wholegrain cereals and other grain foods, reduced fat milk, lentils or other legumes, eggs, and frozen or canned foods without added sugars or added salt – this way you can eat at home more often and cook meals yourself by adding fresh ingredients.
- Choose a variety of types and colours of fresh vegetables and fruits that are in season.
- Try new ways of cooking with vegetables like roasting, baking, barbequing and stir-frying. Add extra vegetables and legumes to your recipes.
- Use fruit for snacks and desserts.
- Lean red meats are important, but a maximum of 455g a week is recommended.
- Include at least 1 or 2 meat-free meals each week – include eggs, legumes such as beans and tofu, and nuts and seeds.
- Choose reduced-fat varieties of milk, yoghurt and cheese.
- Include small amounts of foods rich in unsaturated fats such as oils, spreads, nut butters/pastes and avocado.
- Drink plenty of water instead of drinks with added sugars or alcohol.
- Choose carefully when eating out- limit creamy, commercially baked or fried foods.
- Store unused cooked food in the fridge.
- Older people who have trouble with their teeth, may prefer softer textured or cooked vegetables and fruit, finely milled wholegrain cereal foods and dishes like soups, casseroles or stews.

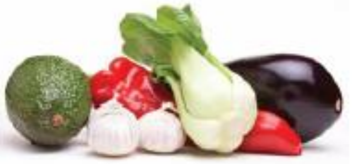

The *Australian Dietary Guidelines* provide up-to-date advice about the amount and kinds of foods that we need to eat for health and wellbeing.

**For more information visit:**
www.eatforhealth.gov.au

**or contact:**
National Health and Medical Research Council
GPO Box 1421
Canberra ACT 2601
13 000 NHMRC (13 000 64672)

**To order print copies contact:**
National Mailing and Marketing
Email: health@nationalmailing.com.au
Phone: 02 6269 1080

Publication Reference: N55g

Australian Government
National Health and Medical Research Council
Department of Health and Ageing

www.eatforhealth.gov.au

## SERVE SIZES

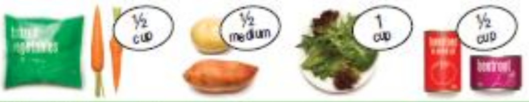


### Vegetables and legumes/beans

| Serves per day | 19-50 years | 51-70 years | 70+ years |
|---|---|---|---|
| Man | 6 | 5½ | 5 |
| Woman | 5 | 5 | 5 |

**A standard serve of vegetables is about 75g (100-350kJ) or:**

- ½ cup cooked green or orange vegetables (for example, broccoli, spinach, carrots or pumpkin)
- ½ cup cooked, dried or canned beans, peas or lentils*
- 1 cup green leafy or raw salad vegetables
- ½ cup sweet corn
- ½ medium potato or other starchy vegetables (sweet potato, taro or cassava)
- 1 medium tomato

*preferably with no added salt

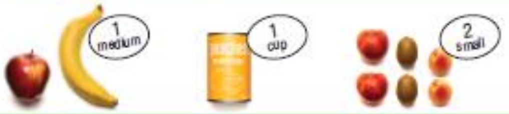


### Fruit

| Serves per day | 19-50 years | 51-70 years | 70+ years |
|---|---|---|---|
| Man | 2 | 2 | 2 |
| Woman | 2 | 2 | 2 |

**A standard serve of fruit is about 150g (350kJ) or:**

- 1 medium apple, banana, orange or pear
- 2 small apricots, kiwi fruits or plums
- 1 cup diced or canned fruit (with no added sugar)

Or only occasionally:

- 125ml (½ cup) fruit juice (with no added sugar)
- 30g dried fruit (for example, 4 dried apricot halves, 1½ tablespoons of sultanas)

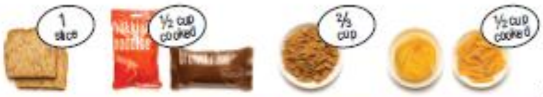


### Grain (cereal) foods, mostly wholegrain and/or high cereal fibre varieties

| Serves per day | 19-50 years | 51-70 years | 70+ years |
|---|---|---|---|
| Man | 6 | 6 | 4½ |
| Woman | 6 | 4 | 3 |

**A standard serve (500kJ) is:**

- 1 slice (40g) bread
- ½ medium (40g) roll or flat bread
- ½ cup (75–120g) cooked rice, pasta, noodles, barley, buckwheat, semolina, polenta, bulgur or quinoa
- ½ cup (120g) cooked porridge
- ⅔ cup (30g) wheat cereal flakes
- ¼ cup (30g) muesli
- 3 (35g) crispbreads
- 1 (60g) crumpet
- 1 small (35g) English muffin or scone

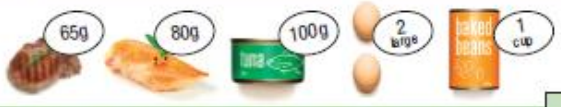


### Lean meat and poultry, fish, eggs, tofu, nuts and seeds, and legumes/beans

| Serves per day | 19-50 years | 51-70 years | 70+ years |
|---|---|---|---|
| Man | 3 | 2½ | 2½ |
| Woman | 2½ | 2 | 2 |

**A standard serve (500-600kJ) is:**

- 65g cooked lean meats such as beef, lamb, veal, pork, goat or kangaroo (about 90-100g raw)*
- 80g cooked lean poultry such as chicken or turkey (100g raw)
- 100g cooked fish fillet (about 115g raw weight) or one small can of fish
- 2 large (120g) eggs
- 1 cup (150g) cooked or canned legumes/beans such as lentils, chick peas or split peas (preferably with no added salt)
- 170g tofu
- 30g nuts, seeds, peanut or almond butter or tahini or other nut or seed paste (no added salt)

*weekly limit of 455g

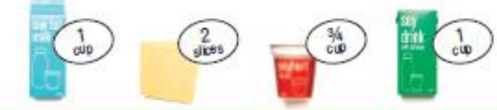


### Milk, yoghurt, cheese and/or alternatives, mostly reduced fat

| Serves per day | 19-50 years | 51-70 years | 70+ years |
|---|---|---|---|
| Man | 2½ | 2½ | 3½ |
| Woman | 2½ | 4 | 4 |

**A standard serve (500-600kJ) is:**

- 1 cup (250ml) fresh, UHT long life, reconstituted powdered milk or buttermilk
- ½ cup (120ml) evaporated milk
- 2 slices (40g) or 4 x 3 x 2cm cube (40g) of hard cheese, such as cheddar
- ½ cup (120g) ricotta cheese
- ¾ cup (200g) yoghurt
- 1 cup (250ml) soy, rice or other cereal drink with at least 100mg of added calcium per 100ml

- **To meet additional energy needs, extra serves from the Five Food Groups or unsaturated spreads and oils, or discretionary choices may be needed only by those adults who are taller or more active, but not overweight.**
- **An allowance for unsaturated spreads and oils for cooking, or nuts and seeds can be included in the following quantities: 28-40g per day for men less than 70 years of age, and 14-20g per day for women and older men.**
- **For meal ideas and advice on how to apply the serve sizes go to:**

**www.eatforhealth.gov.au**

**FOR FURTHER INFORMATION GO TO www.eatforhealth.gov.au**

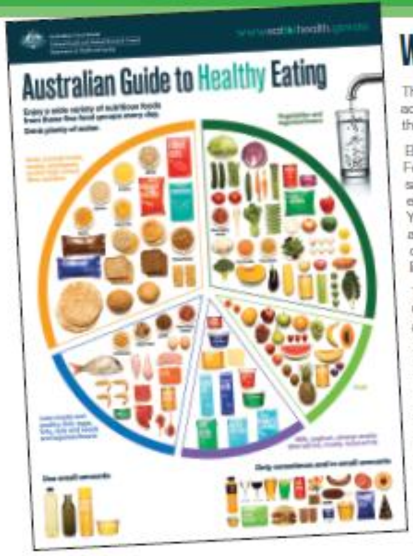


## WHICH FOODS SHOULD I EAT AND HOW MUCH?

The *Australian Dietary Guidelines* provide up-to-date advice about the amount and kinds of foods and drinks that we need regularly, for health and well-being.

By eating the recommended amounts from the Five Food Groups and limiting the foods that are high in saturated fat, added sugars and added salt, you get enough of the nutrients essential for good health. You may reduce your risk of chronic diseases such as heart disease, type 2 diabetes, obesity and some cancers. You may also feel better, look better, enjoy life more and live longer!

The amount of food you will need from the Five Food Groups depends on your age, gender, height, weight and physical activity levels, and also whether you are pregnant or breastfeeding. For example, a 43-year-old man should aim for 6 serves of vegetables a day, whereas a 43-year-old woman should aim for 5 serves a day. A 61-year-old man should aim for 6 serves of grain (cereal) foods a day, and a 61-year-old woman should aim for 4 serves a day. Those who are taller or more physically active (and not overweight or obese) may be able to have additional serves of the Five Food Groups or unsaturated spreads and oils or discretionary choices.

For further information go to www.eatforhealth.gov.au.

### HOW MUCH IS A SERVE?

It's helpful to get to know the recommended serving sizes and serves per day so that you eat and drink the right amount of the nutritious foods you need for health – as shown in the tables above. We've given you the serve size in grams too, so you can weigh foods to get an idea of what a serve looks like.

The 'serve size' is a set amount that doesn't change. It is used along with the 'serves per day', to work out the total amount of food required from each of the Five Food Groups. 'Portion size' is the amount you actually eat and this will depend on what your energy needs are. Some people's portion sizes are smaller than the 'serve size' and some are larger. This means some people may need to eat from the Five Food Groups more often than others.

### HOW MANY SERVES A DAY?

Few people eat exactly the same way each day and it is common to have a little more on some days than others. However, on average, the total of your portion sizes should end up being similar to the number of serves you need each day.

If you eat portions that are smaller than the 'serve size' you will need to eat from the Food Groups more often. If your portion size is larger than the 'serve size', then you will need to eat from the Food Groups less often.

**Appendix 2**

## Are you super hungry?

If you are feeling '*super*' hungry, these foods contain minimal kilojoules and can be eaten in addition to the food we have supplied.

Please make sure you record the name of the food, how much you ate and when you ate it on your checklist.

### *Vegetables / salad**

These may be eaten raw, boiled or steamed. For added flavour please see section 'For added flavour' below.

| | | | | |
|---|---|---|---|---|
| Asparagus | Brussel sprouts | Celery | Lettuce | Silver beet |
| Artichoke | Carrot | Cucumber | Mushrooms | Spinach |
| Bean sprouts | Cabbage | Eggplant | Onion | Squash |
| Bok-choy | Capsicum | Fennel | Rhubarb | Tomato |
| Broccoli | Cauliflower | Green (long) beans | Radish | Zucchini |

**Avoid: potato, sweet potato, pumpkin, peas, corn, beetroot, and parsnip.**

### *Drinks*

- Plain water
- Sparkling/mineral/soda water (no sugar added)
- Diet only cordial
- Diet only soft-drinks
- Herbal teas without milk and sugar
- Black tea/coffee without milk and sugar

### **For added flavour*

Artificial sweeteners may be added to cold or hot drinks.

For the meals provided or for the *extra* vegetables/salads eaten the following flavours may be added:

| | | |
|---|---|---|
| Chili | Herbs, dried | Salt |
| Curry powder | Herbs, fresh | Soy sauce |
| Fat-free & sugar-free dressing | Lemon | Spices (any) |
| Garlic | Lime | Vegemite |
| Ginger | Pepper | Vinegar |

**Avoid: tomato sauce, sweet chilli sauce, and BBQ sauce.**

# Statistical analysis plan

Shifting Weight using Intermittent Fasting in night shift workers (SWIFt) study: a three-arm randomised controlled trial comparing three weight loss strategies in night shift workers with obesity

**Statistical Analysis Plan**

Version 3: Final Version

Date: 13 November 2023

Author: Prof Robert Ware

**Study investigators:**

PRIMARY INVESTIGATORS

Prof Maxine Bonham

CO-INVESTIGATORS

A/Prof Catherine Huggins

Dr Nicole Kellow

Dr Tracey Sletten

Dr Gloria Leung

Dr Rochelle Davis

Ms Angela Clark

Ms Corinne Davis

Prof Siobhan Banks

Prof Alison Coates

Prof Jillian Dorrian

Dr Michelle Rogers

Ms Yan Yin Phoi

Statistical Analysis Plan – Version History

Version 1: was prepared by the investigator team as part of the initial application to the ethics committee, and was submitted 08 August 2019. It was finalised before any data had been collected

Version 2: was prepared by the investigator and statistical team prior to data analysis, and was finalised on 23 May 2023. It was finalised before any data was handled by the statistical team and before investigators were unmasked to group allocation.

Version 3: was prepared by the investigator and statistical team. It is a record of analyses included in the submitted manuscript. It was finalised on 13 November 2023.

See Section 5 of the report for detailed differences between the versions of the statistical analysis plans.

## Table of Contents


1. Administrative information ........ 47
    - 1.1 Purpose ........ 47
    - 1.2 Study identifiers ........ 47
2. Study synopsis ........ 47
    - 2.1 Study Hypotheses ........ 47
        - 2.1.1 Study hypotheses ........ 47
    - 2.2 Outcomes ........ 48
        - 2.2.1 Primary outcome per protocol ........ 48
        - 2.2.2 Secondary outcomes as per protocol ........ 48
    - 2.3 Interventions ........ 49
    - 2.4 Setting ........ 49
    - 2.5 Randomization ........ 49
    - 2.6 Data collection ........ 50
    - 2.7 Sample size ........ 50
3. Statistical analysis ........ 51
    - 3.1 General principles ........ 51
        - 3.1.1 Intention to treat and per protocol analysis ........ 51
        - 3.1.2 Missing Data ........ 51
        - 3.1.3 Sensitivity Analysis ........ 51
        - 3.1.4 Presentation of results ........ 52
        - 3.1.5 Level of significance ........ 52
        - 3.1.6 Stratified analysis ........ 52
        - 3.1.7 Statistical software ........ 52
    - 3.2 Evaluation of demographic and baseline characteristics ........ 53
        - 3.2.1 Participant characteristics ........ 53
    - 3.3 Planned analysis of the co-primary outcomes ........ 54
    - 3.4 Planned analysis of secondary outcomes ........ 55
        - 3.4.1 Table of Secondary Outcomes and Method of Analysis ........ 56
4. Additional Studies using SWIFt RCT Data ........ 58
5. Differences between versions of Statistical Analysis Plans ........ 58
    - 5.1 Differences between Protocol and Initial Analysis Plan ........ 58
    - 5.2 Differences between Initial and Final Analysis Plans ........ 58

5.3 Differences between initial and final versions of the study protocol................................58

6. Trial status..........................................................................................................................59

List of abbreviations

| | |
|---|---|
| IF:2D | 5:2 day |
| IF:2N | 5:2 night |
| AGEs | Advanced glycation end-products |
| AQOL | Assessment of quality of life |
| BMI | Body mass index |
| BP | Blood pressure |
| CER | Continuous energy restriction |
| CRF | Case report form |
| CVD | Cardiovascular disease |
| DASS-21 | Depression, anxiety and stress scale 21 |
| DXA | Dual x-ray absorptiometry |
| HOMA-IR | Homeostatic model assessment for insulin resistance |
| IPAQ | International physical activity questionnaires |
| SOS | Survey of shift workers |
| SWIFt | Shifting Weight using Intermittent Fasting in night shift workers |
| WC | Waist circumference |

- Administrative information

○ **Purpose**

The. Shifting Weight in night shift workers (SWIFt Study) aims is to identify a weight loss strategy for night shift workers that is feasible and flexible to their needs. The purpose for this document is to minimize bias and ensure transparency and internal validity for the findings of the trial, by defining and making publicly available the analysis approach prior to reviewing or analysing any trial data. This statistical analysis plan (SAP) will inform analysis and reporting of the main effectiveness findings of the trial. It provides a detailed description of the primary and secondary trial outcomes and the methods for statistical comparison.

○ **Study identifiers**

- Protocol: https://bmjopen.bmj.com/content/12/4/e060520
- ANZCTR registration number: ACTRN12619001035112

- Study synopsis

This study is a multi-site 18 month parallel randomised trial (six-month intervention with 12-month follow-up). Three weight loss approaches will be compared: daily energy restriction (ER); a 5:2 protocol with fasting during the day (IF:2D); and during the night shift (IF:2N). This study will evaluate the effect of the 3 dietary interventions on weight loss and maintenance, study retention and changes in markers of disease risk. The protocol of this paper has been published previously (1) This statistical analysis plan considers outcomes post-intervention phase, that is, to 6 months post-randomisation. Outcomes at 18 months will be considered in a subsequent analysis and publication (see Section 4)

## 1.1 Study Hypotheses

### 1.1.1 Study hypotheses

In this study of shift workers, the hypotheses are that

- Diet interventions will all result in significant weight loss across time

- Insulin resistance (HOMA-IR) will be lower in the IF:2D compared to ER, and the IF:2N compared to the IF:2D (IF:2N<IF:2D<ER)
- Study retention will be higher in the IF:2D and IF:2N compared to the ER (IF:2D,IF:2N>ER)

## 1.2 Outcomes

Outcomes of weight loss and maintenance, study retention and changes in markers of disease risk. (The definitions are provided in the SWIFT Protocol section 4.3)

### 1.2.1 Primary outcome per protocol

1. Body weight was collected in the clinics at baseline, 3 months, the end of weight loss phase (24 weeks) and the end of the follow-up phase (18 months), height was collected at baseline to calculate BMI (secondary outcome) at each of these time points. When the study began, participants were also to attend the clinic at 2, 4, 6, 8, 16 and 20 weeks of the intervention and 2 and 6 months of the follow-up to collect body weight measurements. However, the protocol was altered on 10/06/2023 due to the COVID-19 pandemic. After this protocol change, body weight was taken at home using Bluetooth scales at these timepoints (this data was emailed to the researcher who logged it in REDCap).
2. HOMA-IR: A fasting venous blood sample (approximately 40 ml) was collected for analysis of fasting blood glucose (Indiko clinical chemistry analyser, ThermoFisher) and fasting insulin (ELISA, Millipore). Samples were collected at baseline, 3, 6 and 18 months. HOMA-IR was calculated from the fasting blood glucose and blood insulin measures collected at baseline, 3, 6, and 18 months using the following formula: (fasting plasma insulin (μU/mL)×fasting plasma glucose (mmol/L))÷22.5 (2).

### 1.2.2 Secondary outcomes as per protocol

1. Biochemical measures: Fasting glucose, insulin, triglycerides, cholesterol (total and subsets) were assessed from venous blood samples collected at baseline, 3, 6 and 18 months
2. Body composition: fat and fat free mass were calculated from DXA measurement, and waist circumference was measured at baseline, 6 and 18 months.
3. Questionnaires: The International Physical Activity Questionnaire Long Form (IPAQ) the Assessment of Quality of Life (AQoL-8D) and the Survey of Shiftworkers (SOS)

short version of the Standard Shiftwork Index were assessed at baseline, 6 and 18 months. These measures were included to describe the population and are validated, reliable and freely available.

4. Dietary intake: 7-day food diaries were collected at baseline, 6, and 18 months.

Please refer to the study protocol for a full list of outcomes measured.

## 1.3 Interventions

- 1. IF:2D - 5:2 Day fasting diet: For five days of the week participants ate their usual diet, and on two days of the week (the two 'fast' days) they were required to restrict their energy intake to 2100 kJ/day for females and 2500 kJ/day for males during the day shift.
- 2. IF:2N - 5:2 Night fasting diet: For five days of the week participants ate their usual diet, and on two days of the week (the two 'fast' days) they were required to restrict their energy intake to approximately 2100 kJ/day for females and 2500 kJ/day for males during the night shift.
- 3. CER - 20% Continuous daily energy restriction (20%CER): Based on a participant's age, height and body weight, the Mifflin ST Jeor equation was used to calculate basal metabolic rate (BMR). Taking into consideration a participant's physical activity levels (PAL), these calculated energy requirements were reduced by 20%.

## 1.4 Setting

This study was set in the general community. Recruitment occurred either by word-of-mouth, promotion via media announcements, social media (i.e., Twitter, Facebook, Instagram), flyers or through contact with organisations that employ shift workers, including, but not limited to, those in the industries of; healthcare and/or clinical facilities, emergency services, manufacturing, facility maintenance and security, and transport. Participants were not recruited whilst attending a healthcare and/or clinical facility as a patient or client.

## 1.5 Randomization

Participants were randomly allocated to one of three intervention groups, using the NHMRC Clinical Trials Centre randomisation service.

## 1.6 Data collection

Measures of height, body weight, body mass index (BMI), waist circumference (WC), and blood pressure (BP) were recorded directly into the Case Report Form (CRF) on REDCap and this was considered the source data. A hardcopy of the Case Report Forms (CRF) was stored in a secure cabinet on each site. Electronic copies of results of blood and faecal analysis were stored on labArchives. Questionnaires were completed directly in REDCap and are source data. The source data collected from DXA scans was stored as electronic files on the DXA machine and on a secure electronic server and also in hard copy in a secure cabinet on each site. Hardcopy food diaries were stored in a secure cabinet on each site. Dietary information was stored with the CRF in labArchives.

## 1.7 Sample size

*A priori* sample size calculations were initially determined on identifying significant within-group changes in body weight and HOMA-IR. For weight loss, a small-medium within-subject effect (f=0.16) from a 14-week workplace weight loss program (3) was used as a minimum expected amount of weight loss in a given study group over a 6-month time period and required 93 participants per group (α=0.05, 1−β=0.8). We also proposed a differential effect of the diets across time on HOMA-IR. Effect sizes for differences in night and day shift workers in HOMA-IR ranged from small–medium (f=0.14) (4) to medium–large (f=0.33) (5) and powering for a small–medium interaction effect required 111 participants per group (α=0.05, 1−β=0.8). Since our primary hypotheses are different, and independent, to avoid unnecessarily increasing our type 2 error rates (6, 7) we argued that family-wise p-value adjustment for our main analyses was not appropriate. Allowing for 27% drop-out, our aim was to recruit 423 individuals to prove our hypotheses. When recruitment was affected by the COVID-19 pandemic the sample size calculations were revisited. A review of our original statistical power using newly available data was presented as an alternative to our original sample size calculations (1), and the requirements were for a total of 244 and 300 participants to be enrolled to detect a change in body weight and HOMA-IR respectively (after accounting for anticipated drop-out). When considering between-group differences, with 80% power and alpha=0.0125 (global alpha=0.05) these recruitment numbers allow identification of between-group differences of 0.5SDs or greater.

- Statistical analysis

## 1.8 General principles

### 1.8.1 Intention to treat and per protocol analysis

The key analysis principle is intention to treat analysis. All participants who were randomized to a study group and supplied baseline data, will be analysed in the group they were allocated to, regardless of intervention compliance.

Per protocol analysis will be performed to test the sensitivity of the results to noncompliance. All participants who were randomized and supplied baseline data and 6 month data will be included in the per protocol analysis. Note that in the manuscript this is called the 'completers analysis'.

### 1.8.2 Missing Data

The number of missing observations for each primary and secondary outcome will be reported by study group. If the proportion of missing observations is non-trivial (>5%), consideration will be given to the possible introduction of compliance and attrition bias. The cause of any missing data will be assessed.

Any outcome exhibiting >5% missingness at random will undergo imputation for the missing data. Data will be imputed using chained equation multiple imputation. Imputation variables will be determined by examining characteristics associated with participant drop-out.

### 1.8.3 Sensitivity Analysis

The sensitivity of the findings to missing data will be investigated through the presentation of different models. The primary analysis will be a 'full data' model, where all recorded observations for the outcome of interest are included in the analysis using a mixed-effects regression model. Following this a series of sensitivity analyses will be conducted for the

- Full data adjusting for stratification factors. A mixed effects model will include stratification factors as covariables.

- Full data adjusting for covariables affected by drop-out. A mixed effects model adjusting for variables that were associated with attrition.
- Multiple imputation will be conducted with missing data assumed to be missing at random unless otherwise indicated.
- Complete case. Individuals will be included if they provide data at baseline and 6 months.

### 1.8.4 Presentation of results

Continuous data will be summarized descriptively using either mean and standard deviation (SD) or median and interquartile range (IQR), depending on the distribution of the variable of interest. Categorical data will be presented as frequencies and percentages.

### 1.8.5 Level of significance

A significance level of alpha = 0.0125 will be used to evaluate statistical significance for the primary outcome (two comparisons, 20%CER vs 5:2 Day Fasting and 20%CER vs 5:2 Night Fasting, so global alpha=0.05). Secondary outcomes and subgroup analyses will not be compared using formal hypothesis testing procedures. Instead 95% confidence intervals will be presented without adjustment for multiplicity.

### 1.8.6 Stratified analysis

For the primary outcome we will be stratify by site, to investigate the level of variability across strata. We will test the heterogeneity between sites by running a model with time-point and site as main effects, and a time-by-site interaction term, and will report the level of interaction using the likelihood ratio test.

### 1.8.7 Statistical software

All estimates will be derived using Stata v14.1 (StataCorp, College Station, Texas, USA).

## 1.9 Evaluation of demographic and baseline characteristics

### 1.9.1 Participant characteristics

| Variable | Description | Data type | Method of presentation | Variable Name | Instrument |
|---|---|---|---|---|---|
| Age | Years | Continuous | Mean, SD and range | age_1 | Questionnaire |
| Sex | Male, Female, other | Nominal | Counts and percentages | gender_identity | Questionnaire |
| Body weight | kg | Continuous | Mean, SD and range | weight_kg_rounded | Clinic visit |
| Body Composition | kg | Continuous | Mean, SD and range | total_fat_mass_kg<br>total_fat_free_mass_kg | Clinic visit |
| BMI | kg/m2 | Continuous | Mean, SD and range | BMI | Clinic visit |
| Ethnicity,<br>- European<br>- Asian<br>- Oceania<br>- North African and Middle Eastern<br>- People of the Americas<br>- Other | Categorical | Nominal | Counts and percentages | Ethnicity<br>other_ethnicity | Questionnaire |
| Self-reported shift schedule<br>- Fixed night shift<br>- Rotating night shift<br>- Other | Categorical | Nominal | Counts and percentages | what_is_your_current_shift | Questionnaire |
| Years in shift work | Years | Continuous | Mean, SD and range | how_long_worked_altogether | Questionnaire |
| Blood pressure<br>- Systolic<br>- Diastolic | mmHg | Continuous | Mean, SD and range | - Blood Pressure Systolic _rounded<br>- Blood Pressure Diastolic_rounded | Clinic visit |
| Glucose | mg/dL | Continuous | Mean, SD and range | Glucose_meanofdup | Clinic visit |
| Insulin | mU/mL | Continuous | Mean, SD and range | Insulin_mean_μIU/mL | Clinic visit |
| HOMA-IR | (Glucose × insulin)/22.5 | Continuous | Mean, SD and range | HOMA_IR | Clinic visit |

| | | | | | |
|---|---|---|---|---|---|
| Triglycerides | mg/dL | Continuous | Mean, SD and range | TAG_dupmean | Clinic visit |
| Total Cholesterol | mg/dL | Continuous | Mean, SD and range | chol_dupmean | Clinic visit |
| LDL-Cholesterol | mg/dL | Continuous | Mean, SD and range | LDL_mean | Clinic visit |
| HDL-Cholesterol | mg/dL | Continuous | Mean, SD and range | HDL_dupmean | Clinic visit |
| Average daily energy intake | kJ / day | Continuous | Mean, SD and range | EnergyDF_kJ | Diet diaries |
| Physical activity<br>- Total MET mins/week | mins | Continuous | Median, Interquartile Range | totMETmins_wk | International Physical Activity Questionnaire - Long Form |
| Sleep episode duration on night shifts | Mins/24hr | Continuous | Mean, SD and range | Sleepduration_Between two successive night shifts | Survey of Shiftworkers |
| Sleep episode duration on days off | Mins/24hr | Continuous | Mean, SD and range | Sleep duration_Between two successive days off | Survey of Shiftworkers |
| Quality of life | Score | Continuous | Mean, SD and range | AQoL8DUtility | AQoL-8D |

### 1.10 Planned analysis of the co-primary outcomes

The first co-primary outcome, body weight measured in kilograms at 6 months, will be compared within and between groups as a continuous outcome. Summary statistics for each group will be presented as mean (SD) by time-point. The primary analysis will use data collected at 0, 3, and 6 months. A mixed-effects linear regression model will be constructed using intervention group and time as main effects, and include a group-by-time interaction term. Participant will be included as a random effect (random intercept). The first co-primary outcome, difference in body weight between-groups at 6 months, will be calculated using post-estimation commands from the mixed-effects model, and effect estimates will be reported as mean difference and 95% confidence intervals. The statistical significance of the within-group changes will be reported as a P-value.

The second co-primary outcome, HOMA-IR measured as fasting insulin (mIU/L) x fasting glucose (mmol/L)/22.5.in kilograms at 6 months, will be compared within and between groups as a continuous outcome. Summary statistics for each group will be presented as mean (SD) by time-point. The primary analysis will use data collected at 0, 3, and 6 months. A mixed-

effects linear regression model will be constructed using intervention group and time as main effects, and include a group-by-time interaction term. Participant will be included as a random effect (random intercept). The second co-primary outcome, difference in between-group HOMA-IR at 6 months, will be calculated using post-estimation commands from the mixed-effects model, and effect estimates will be reported as mean difference and 95% confidence intervals. The statistical significance of the within-group changes will be reported as a P-value.

### 1.11Planned analysis of secondary outcomes

The analysis of secondary outcomes will concentrate on the difference in outcomes between the CER strategy and the two IF strategies. In general, these between-group differences will be investigated by constructing mixed-effects regression models. The form of the model will depend on the distribution of the outcome being investigated. For outcomes measured on an interval scale, mixed-effects linear models will be constructed. For outcomes measured on a binary scale, mixed-effects logistic models will be constructed. For outcomes measured on a count scale, mixed-effects Poisson models will be constructed. The assumptions underlying each model will be tested, and a non-parametric model may be used for any outcome if appropriate. The models will contain two main effects; time-point, a three-level variable, and intervention group, a three-level variable. The models will contain one random effect; participant. By default the random effect will be a random intercept, but for each outcome we will compare findings from a model with a random intercept against findings from a model with a random intercept and slope using the likelihood ratio test. If the model with the random intercept and slope is significantly better at $P<0.05$ then this is how the intercept will be treated in the final model.

Secondary outcomes will be analysed as follows in Section 3.4.1.

### 1.11.1 Table of Secondary Outcomes and Method of Analysis

| **Measure** | **Data Source** | **Distribution of outcome** | **Regression model** | **Main effects** | **Random intercept** |
|---|---|---|---|---|---|
| Body mass index (kg/m2) | Measured weight (kg) and height (m) | Normal | Gaussian | Time-period Intervention Group | Participant |
| Fat mass (kg) | DXA | Normal | Gaussian | Time-period Intervention Group | Participant |
| Fat-free mass (kg) | DXA | Normal | Gaussian | Time-period Intervention Group | Participant |
| Waist circumference | Measured waist (cm) | Normal | Gaussian | Time-period Intervention Group | Participant |
| Blood pressure | Sphygmomanometer (mm Hg) | Normal | Gaussian | Time-period Intervention Group | Participant |
| Fasting insulin | ELISA (mU/mL) | Normal | Gaussian | Time-period Intervention Group | Participant |
| Fasting glucose | Indiko (mg/dL) | Normal | Gaussian | Time-period Intervention Group | Participant |
| Fasting Triglycerides | Indiko (mg/dL) | Normal | Gaussian | Time-period Intervention Group | Participant |

| | | | | | |
|---|---|---|---|---|---|
| Fasting cholesterol (total and subsets) | Indiko (mg/dL) | Normal | Gaussian | Time-period Intervention Group | Participant |
| Quality of life | AQoL-8D | Normal | Gaussian | Time-period Intervention Group | Participant |
| Physical activity (total MET minutes / week) | International Physical Activity Questionnaire - Long Form | Non-normal | Median | Time-period Intervention Group | Participant |
| Sleep episode duration on night shifts (hours) | Survey of Shift workers | Normal | Gaussian | Time-period Intervention Group | Participant |
| Sleep episode duration on days off (hours) | Survey of Shift workers | Normal | Gaussian | Time-period Intervention Group | Participant |
| Habitual dietary intake (kJ /Day) | 7-day food dairy | Normal | Gaussian | Time-period Intervention Group | Participant |
| Compliance and retention | Time to dropout | Normal | Logrank | Time-period Intervention Group | Participant |

- Additional Studies using SWIFt RCT Data

As part of the study design, outcome data is collected at baseline, 3 months, 6 months (end of the weight-loss phase) and 18 months (end of the follow-up phase). The analysis plan detailed in this document is for analysis up to the end of the weight-loss phase. When the follow-up phase is complete, an analysis of the retention effects of the three interventions will be performed. The primary outcomes will be body weight and HOMA-IR, and the primary comparisons of interest are between-arm differences. Analyses will follow similar principles to the weight-loss phase analysis.

- Differences between versions of Statistical Analysis Plans

### 1.12 Differences between Protocol and Initial Analysis Plan

**1.13** The study protocol was published in the journal BMJ Open (1). As is the nature of Protocol Papers, it was necessarily brief and contained detailed information on only the analysis of the primary outcomes only. Primary outcomes were to be compared with mixed-effects regression models containing 'time' and 'group' as main effects, with a 'time-by-group' interaction, which is the method retained in the initial and final analysis plans, however the focus in the protocol was on the within-group change, whereas in the subsequent analysis plans the focus of the analysis was on the between-group differences at 6 months. It was proposed 95%CI would be calculated using bootstrapping, but in the subsequent analysis plans we specified we would use Wald-based methods.Differences between Initial and Final Analysis Plans

- In the Final Analysis Plan more detail has been included regarding the sample size calculations, in particular the between-group detectable alternatives are included.
- There was a small change between the participant characteristics we specified we would report in the Initial and Final Plans. The variable listed in the Initial Plan not reported was emotional state measured by the Depression, Anxiety and Stress Scale 21 (DASS 21).
- There were a small number of changes in the secondary outcomes reported. Secondary Outcomes listed in the Initial Analysis Plan not included in the Final Plan were: HbA1c, DASS-21. Secondary outcomes reported in the Final, but not Initial, Plan were: Body weight assessed by Bluetooth scales data.

### 1.14 Differences between initial and final versions of the study protocol

- June 2020: Mode of dietetic consultation changed from face-to-face consultations that were held onsite at research centre clinics to face-to-face video conferencing using Zoom, to comply with restrictions imposed due to the Covid-19 pandemic.

- October 2020: Amendment to qualitative component of project to strengthen the methodology for the mixed-methods evaluation of the SWIFt study, participants will have the opportunity to participate in semi-structured interviews and longitudinal audio diaries.
- February 2022: amendment of inclusion/exclusion criteria as below;
  - Hyperlipidemia removed as inclusion/exclusion criteria as has never been included in any version of screening questions.
  - Waist circumference in isolation removed from exclusion criteria, as is not to be used in isolation as an exclusion criterion but should be taken into consideration if a borderline BMI is presented. <94 cm (Non-Asian men), <90 cm (Asian men) < 80 cm (women). Participants with borderline BMI (27.5 – 28.0 kg/m2 for non-Asian and 25.5 – 26 kg/m2 for Asian participants) and low waist circumference will be excluded. Borderline BMI and waist circumference above pre-determined cutoffs (as above) can be discussed as a team and considered eligible.
  - Included clarification of case-by-case assessment for exclusion due to taking medications known to alter body composition or metabolism; if they have indicated that this is has not been dose stable in the past 3-6 months.
  - Included "does not require drug therapy for diabetes" in addition to inclusion criteria of not diagnosed with Type 2 Diabetes.
- March 2022: addition of questionnaire to collect data on previous infection with Covid-19 (whilst participating in study)
- Jan 2023: Addition of an optional two-year follow-up visit for participants who have completed the SWIFt trial to enable us to look at the long-term impacts of the weight-loss intervention of our shift-work participants.
- the following updates to the Clinical Trials Registry 21/12/2021:
  - HbA1c added as secondary outcome
  - Secondary outcomes have been updated to indicate they are exploratory: Sleep outcomes, gut microbiome, diet intake, physical activity, questionnaires, timed up and go.

- Trial status

The SWIFT trial opened to recruitment on 10/10/2019. There were significant delays to recruitment and follow-up due to COVID-19. The last 6-month outcome was collected on 04/08/2022, and the last 18 month outcome on 23/08/2023. The initial SAP was finalized after data collection was

complete, but data cleaning was ongoing. This version of the SAP reports the analyses of primary and secondary outcomes that was conducted.

## References


1. Rogers M, Coates A, Huggins CE, Dorrian J, Clark AB, Davis C, et al. Study protocol for the Shifting Weight using Intermittent Fasting in night shift workers (SWIFt) study: a three-arm randomised controlled trial comparing three weight loss strategies in night shift workers with obesity. BMJ Open. 2022;12(4):e060520.
2. Matthews DR, Hosker JP, Rudenski AS, Naylor BA, Treacher DF, Turner RC. Homeostasis model assessment: insulin resistance and β-cell function from fasting plasma glucose and insulin concentrations in man. Diabetologia. 1985;28(7):412-9.
3. Morgan PJ, Collins CE, Plotnikoff RC, Cook AT, Berthon B, Mitchell S, et al. Efficacy of a workplace-based weight loss program for overweight male shift workers: the Workplace POWER (Preventing Obesity Without Eating like a Rabbit) randomized controlled trial. Prev Med. 2011;52(5):317-25.
4. Padilha HG, Crispim CA, Zimberg IZ, Folkard S, Tufik S, de Mello MT. Metabolic responses on the early shift. Chronobiol Int. 2010;27(5):1080-92.
5. Akour A, Farha RA, Alefishat E, Kasabri V, Bulatova N, Naffa R. Insulin resistance and levels of cardiovascular biomarkers in night-shift workers. Sleep and Biological Rhythms. 2017;15(4):283-90.
6. Cabin RJ MR. To Bonferroni or not to Bonferroni: when and how are the questions. Bulletin of the Ecological Society of America. 2000;81:246-8.
7. Perneger TV. What's wrong with Bonferroni adjustments. BMJ. 1998;316:1236-8.
